# Syntheses and crystal structures of 4-benzyl-1-ethyl-1,2,4-triazolium bromide and its corresponding NHC complexes of rhodium and iridium

**DOI:** 10.1107/S2056989025002671

**Published:** 2025-03-27

**Authors:** Timothy G. Lerch, Daniel R. Albert, Michael Gau, Edward Rajaseelan

**Affiliations:** a Department of Chemistry Millersville University,Millersville PA 17551 USA; b Department of Chemistry, University of Pennsylvania, Philadelphia, PA 19104, USA; University of Aberdeen, United Kingdom

**Keywords:** crystal structure, triazolium salt, N-heterocyclic carbene, iridium, rhodium, cationic complexes

## Abstract

The syntheses and crystal structures of a triazolium salt, 4-benzyl-1-ethyl-1,2,4-triazolium bromide, C_11_H_14_N_3_^+^·Br^−^ (**2**), and the corresponding N-heterocyclic carbene complexes, (4-benzyl-1-ethyl-1,2,4-triazol-5-yl­idene)chlorido­[(1,2,5,6-η)-cyclo­octa-1,5-diene]rhodium(I), [RhCl(C_8_H_12_)(C_11_H_13_N_3_)] (**3**), (4-benzyl-1-ethyl-1,2,4-triazol-5-yl­idene)[(1,2,5,6-η)-cyclo­octa-1,5-diene](tri­phenyl­phosphane)iridium(I) tetra­fluorido­borate, [Ir(C_8_H_12_)(C_11_H_13_N_3_)(C_18_H_15_P)]BF_4_ (**5**), and (4-benzyl-1-ethyl-1,2,4-triazol-5-yl­idene)[(1,2,5,6-η)-cyclo­octa-1,5-diene](tri­cyclo­hexyl­phosphane)iridium(I) tetra­fluorido­borate dicholoro­methane sesquisolvate, [Ir(C_8_H_12_)(C_11_H_13_N_3_)(C_18_H_33_P)]BF_4_·1.5CH_2_Cl_2_ (**6**), are presented. Complexes **2** and **6** crystallize in the monoclinic space group *P*2_1_/*c*, complex **3** in the triclinic space group *P*1 and complex **5** in the triclinic space group *P*1 with two mol­ecules in the asymmetric unit. The three metal complexes **3**, **5**, and **6** have a distorted square-planar geometry around the metal ions. The N1—C1—N3 bond angle in the triazolium salt **2** is 107.1 (2)° and is observed in the range of 102.2 (3) to 103.8 (5)° in the NHC ligands in complexes **3**, **5**, and **6**. The two substituent ‘wing tips’ in the NHC ligand (N-ethyl and N-benz­yl) are oriented in an *anti*-arrangement in compounds **2** and **3**, a *syn*-arrangement in compound **6**, and both *syn* and *anti*-arrangements in the two independent ion pairs in compound **5**. All structures exhibit non-classical hydrogen-bonding inter­actions with the most acidic hydrogen atoms in complexes **2** and **3** playing critical roles in the orientations of structural units.

## Abstract 2

The syntheses and crystal structures of a triazolium salt, 4-benzyl-1-ethyl-1,2,4-triazolium bromide, and the corresponding N-heterocyclic carbene complexes, (4-benzyl-1-ethyl-1,2,4-triazol-5-yl­idene)chlorido­[(1,2,5,6-η)-cyclo­octa-1,5-diene]rhodium(I), (4-benzyl-1-ethyl-1,2,4-triazol-5-yl­idene)[(1,2,5,6-η)-cyclo­octa-1,5-diene](tri­phenyl­phosphane)iridium(I) tetra­fluorido­borate and (4-benzyl-1-ethyl-1,2,4-triazol-5-yl­idene)[(1,2,5,6-η)-cyclo­octa-1,5-diene](tri­cyclo­hexyl­phosphane)iridium(I) tetra­fluorido­borate dicholoro­methane sesquisolvate, are presented. All structures exhibit non-classical hydrogen-bonding inter­actions with the most acidic hydrogen atoms in two of them playing critical roles in the orientations of structural units.

## 1. Chemical context

Asymmetric 1,2,4-triazolium cations are of inter­est due to their utility as cations in ionic liquids and as precursors to N-heterocyclic carbenes (NHCs) (Chianese *et al.*, 2004 ▸; Dwivedi *et al.*, 2014 ▸). The crystal structures of several triazolium salts have been reported (Albert *et al.*, 2025 ▸; Maynard *et al.*, 2023 ▸; Kumasaki *et al.*, 2021 ▸, El Bakri *et al.*, 2016 ▸; Guino-o *et al.*, 2015 ▸). NHCs have emerged as universal spectator ligands and as alternatives for phosphanes in transition-metal compounds (Herrmann & Köcher, 1997 ▸; Bourissou *et al.*, 2000 ▸; Weskamp *et al.*, 2000 ▸). They form strong bonds to metal centers (Bortenschlager *et al.*, 2005 ▸) and numerous and ever increasing applications of NHCs as supporting ligands in late-transition-metal catalysis have been reported (Díez-Gonzáles *et al.*, 2009 ▸; Cazin, 2013 ▸; Rovis & Nolan, 2013 ▸). Their catalytic activity in the transfer hydrogenation of unsaturated bonds is of great inter­est and it exemplifies some of the key aspects of green chemistry (Ruff *et al.*, 2016 ▸; Zuo *et al.*, 2014 ▸). Steric and electronic tuning of NHCs is possible by changing the ‘wing tip’ substituents on the nitro­gen atoms (Díez-Gonzáles & Nolan, 2007 ▸; Gusev, 2009 ▸; Mata *et al.*, 2004 ▸). Many imidazole- and triazole-based NHC rhodium and iridium complexes have been synthesized and structurally characterized (Herrmann *et al.*, 2006 ▸; Wang & Lin 1998 ▸; Nichol *et al.*, 2009 ▸, 2010 ▸, 2011 ▸, 2012 ▸; Idrees *et al.*, 2017*a* ▸,*b* ▸; Rood *et al.*, 2021 ▸; Rushlow *et al.*, 2021 ▸; Newman *et al.*, 2021 ▸; Castaldi *et al.*, 2021 ▸; Lerch *et al.*, 2024 ▸). Their catalytic activity in the transfer hydrogenation of ketones and imines has also been studied and reported (Hillier *et al.*, 2001 ▸; Albrecht *et al.*, 2002 ▸; Gnanamgari *et al.*, 2007 ▸). In this study we report the syntheses and crystal structures of a new triazolium salt and its corresponding NHC complexes of a neutral rhodium complex and two cationic iridium complexes with different ancillary phosphane ligands.
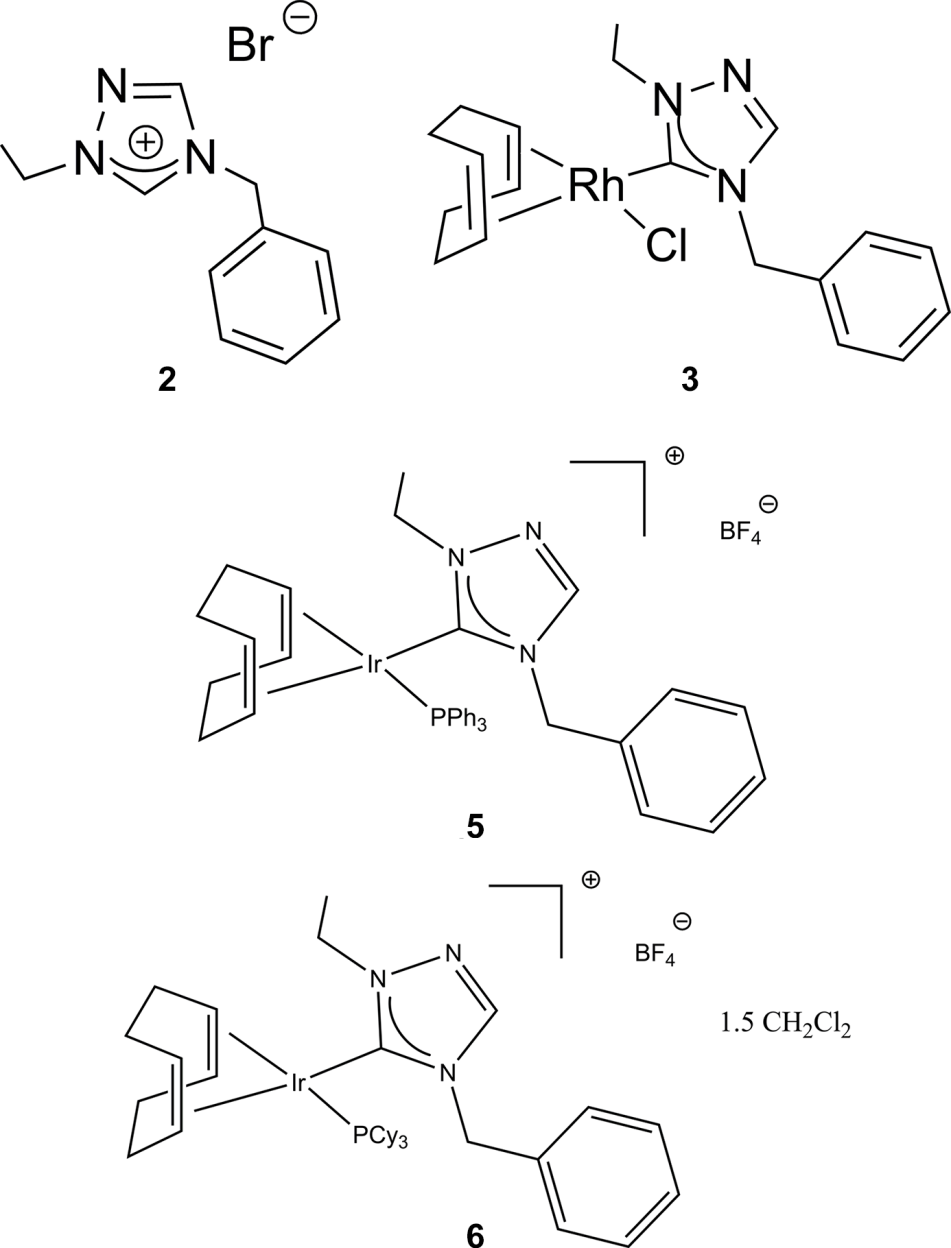


## 2. Structural commentary

**Figure 1:**
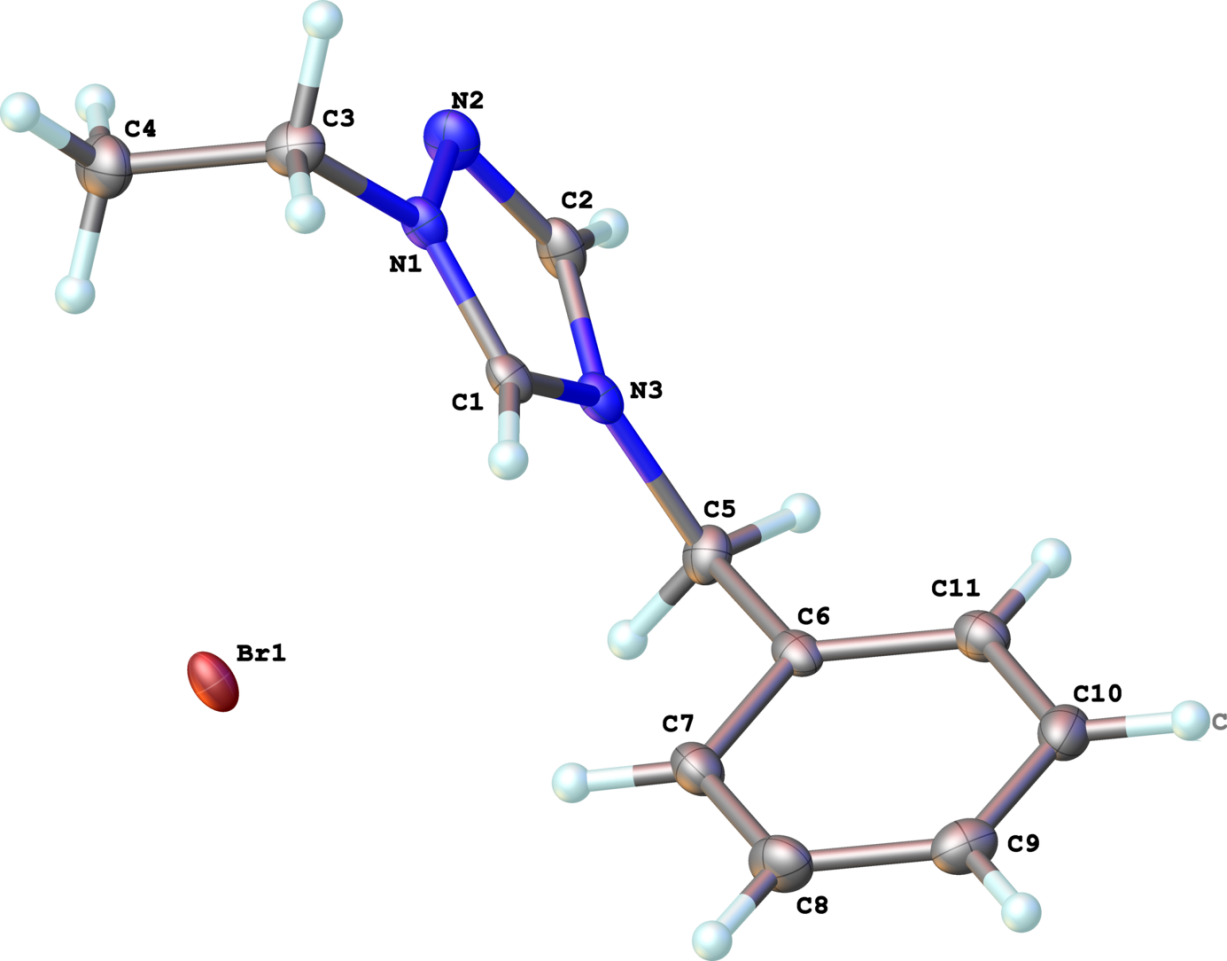
The mol­ecular structure of compound **2**. Ellipsoids represent 50% probability levels.

The triazolium salt (**2**), C_11_H_14_N_3_^+^·Br^−^, crystallizes in the monoclinic space group *P*2_1_/*c* as shown in Fig. 1 ▸. The bond lengths in the triazolium rings indicate aromaticity with C—N bonds exhibiting distances in the range 1.304 (4) to 1.365 (3) Å and an N—N bond distance of 1.379 (3) Å; the N—C—N bond angles in the triazolium ring range from 107.1 (2) to 112.1 (2)°.

**Figure 2:**
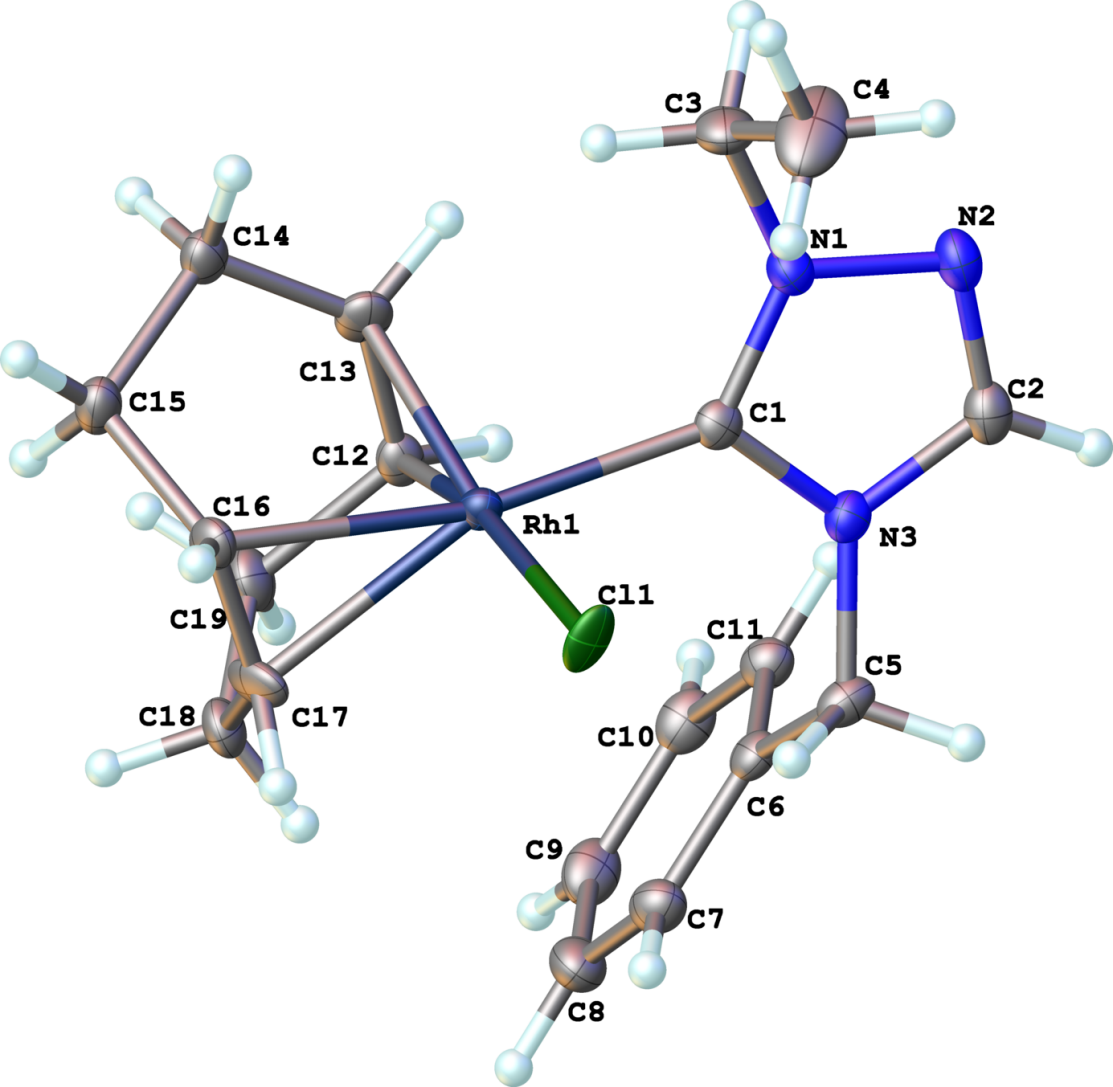
The mol­ecular structure of compound **3**. Ellipsoids represent 50% probability levels. Disordered atoms of the COD ligand (C12–C19) are not shown.

The neutral complex (**3**), Rh(η^2^,η^2^-C_8_H_12_)(C_11_H_13_N_3_)Cl, as illustrated in Fig. 2 ▸, crystallizes in the triclinic space group *P*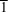. The coordination sphere around the Rh^I^ ion is formed by the bidentate COD ligand and the monodentate NHC and chloride ligands, resulting in a distorted square-planar geometry. The carbene atom, C1, deviates from the expected *sp*^2^ hybridization in that the N1—C1—N3 bond angle in the triazole-based carbene is 102.7 (2)°. Other key bond lengths and angle in the structure are: Rh1—C1(NHC) = 2.014 (3) Å, Rh1—Cl1 = 2.3960 (6) Å, and C1—Rh1—Cl1 is 89.14 (7)°.

**Figure 3:**
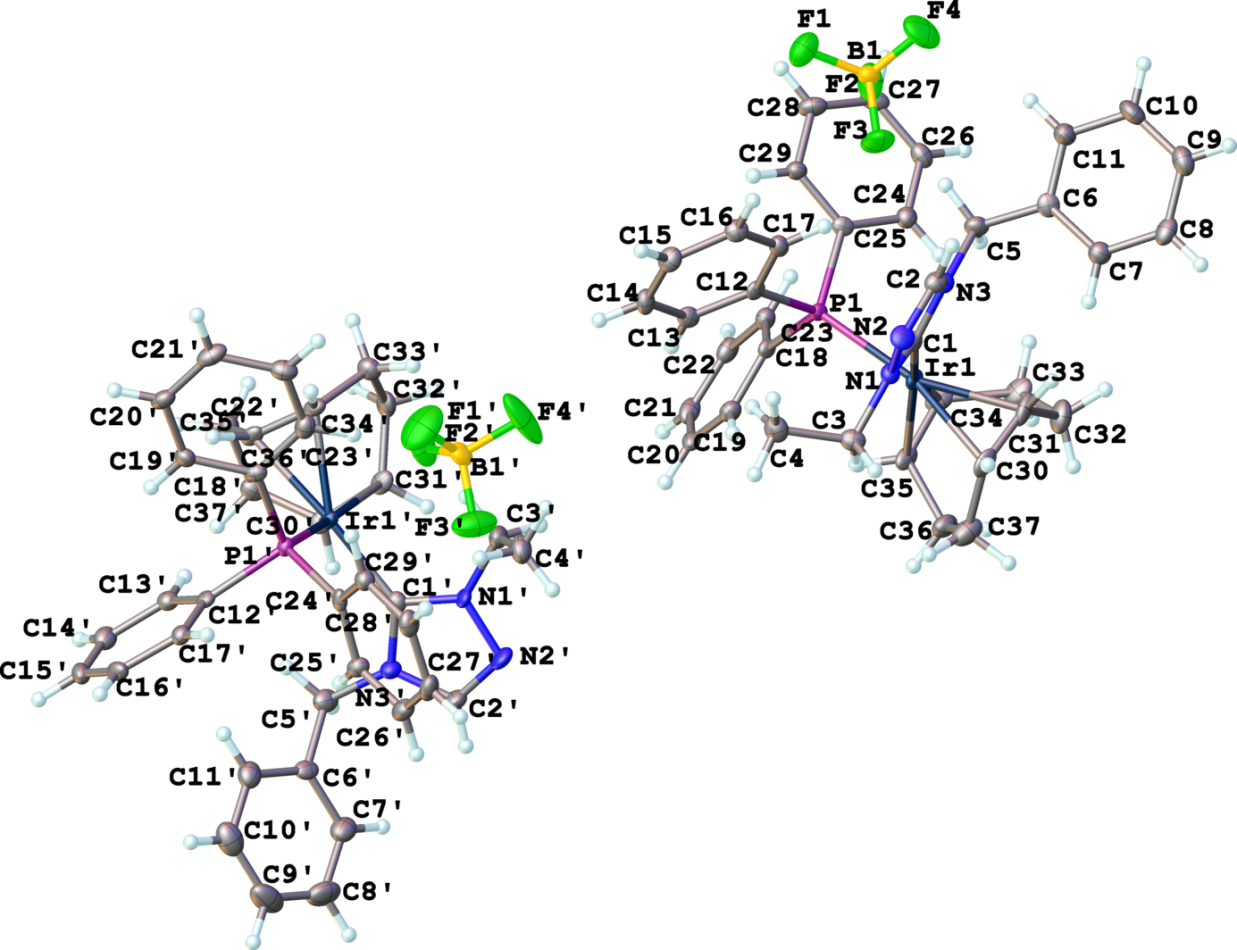
The mol­ecular structure of compound **5**. Ellipsoids represent 50% probability levels. The cation containing Ir1 is designated as **5A** and that containing Ir1′ is designated as **5B**.

Compound (**5**), [Ir(η^2^,η^2^-C_8_H_12_)(C_11_H_13_N_3_)(C_18_H_15_P)]^+^·BF_4_^−^, comprises a cationic iridium complex and a tetra­fluorido­borate counter-anion, as shown in Fig. 3 ▸. Two cations (A containing Ir1 and B containing Ir1′) and two anions are contained in the asymmetric unit, which crystallizes in the triclinic space group *P*1. The distorted square-planar geometry around the iridium ion arises from the bidentate (1,2,5,6-η)-cyclo­octa-1,5-diene (COD) ligand, and the monodentate NHC and tri­phenyl­phospane ligands. It is characterized by C1—Ir—P bond angles of 93.14 (17)° for cation A and 94.64 (18)° for cation B. The N—C—N bond angles of the NHC ligand are 103.8 (5) and 102.7 (5)° for cations A and B, respectively. The metal—phospho­rus bond lengths are 2.3302 (15) Å (cation A) and 2.3217 (15) Å (cation B) and the metal—carbene bond lengths are 2.039 (6) Å and 2.029 (6) Å for cations A and B, respectively.

**Figure 4:**
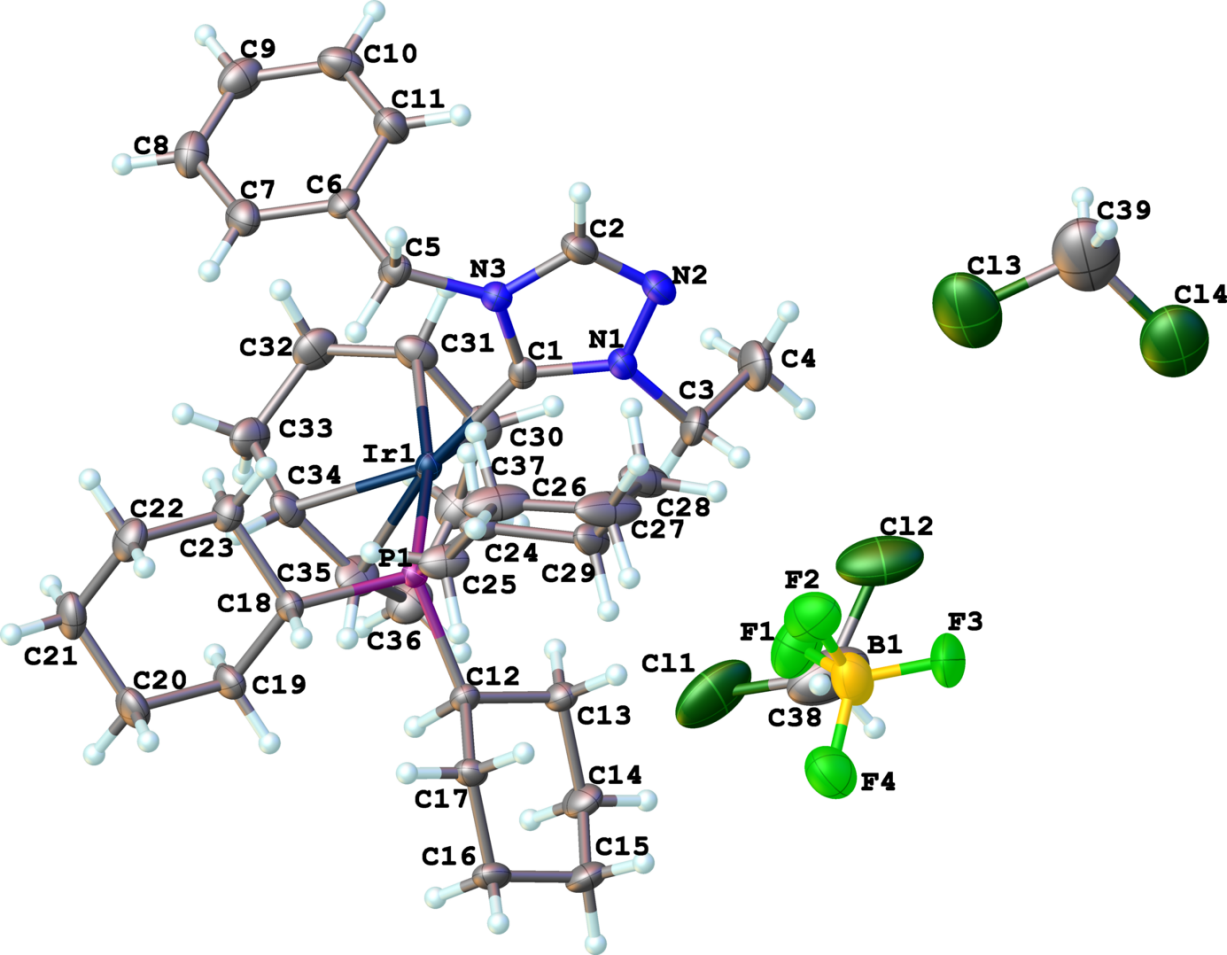
The mol­ecular structure of compound **6**. Ellipsoids represent 50% probability levels. Disordered atoms of the COD ligand, tetra­fluorido­borate anion, and di­chloro­methane solvent are not shown.

Compound (**6**), [Ir(η^2^,η^2^-C_8_H_12_)(C_11_H_13_N_3_)(C_18_H_33_P)]^+^·BF_4_^−^·1.5CH_2_Cl_2_, comprises a cationic iridium complex, a tetra­fluorido­borate counter-anion, and solvating di­chloro­methane (DCM), Fig. 4 ▸. The complex crystallizes in the monoclinic space group *P*2_1_/*c* with four formula units in the unit cell. The IrI center of the cationic complex has a distorted square-planar conformation, formed by a cyclo­octa-1,5-diene (COD) ligand, an N-heterocyclic carbene, and a tri­cyclo­hexyl­phosphane ligand. There are several disordered atoms/mol­ecules that were modeled appropriately: one DCM mol­ecule sits on a crystallographic center of symmetry and was modeled with statistical occupancy. Another DCM mol­ecule, COD and BF_4_ were modeled for positional disorder. The N1—C1—N3 bond angle in the carbene is 102.2 (3)°. Other selected bond lengths and angle in the structure are Ir1—C1 = 2.034 (4) Å, Ir1—P1 = 2.3707 (9) Å, and C1—Ir1—P1 = 93.42 (10)°.

A comparison of the triazolium salt (**2**) bond angles and bond lengths to its corresponding NHC ligands in complexes **3**, **5**,and **6**, show significant changes. Key bond lengths and angles for the structures are summarized in Tables 1 ▸–4 ▸ ▸ ▸. The N1—C1—N3 bond angle changes from 107.1 (2)° in **2** to a range of 102.2 (3)° to 103.8 (5)° in complexes **3**, **5**,and **6**. The C1—N1 and C1—N3 bond lengths change from 1.315 (3) and 1.339 (3) Å in compound **2** to a range from 1.336 (8) to 1.352 (5) Å in compounds **3**, **5**,and **6**, and 1.339 (3) to a range of 1.319 (5) and 1.380 (8) Å in compounds **3**, **5**, and **6** respectively.

**Figure 5:**
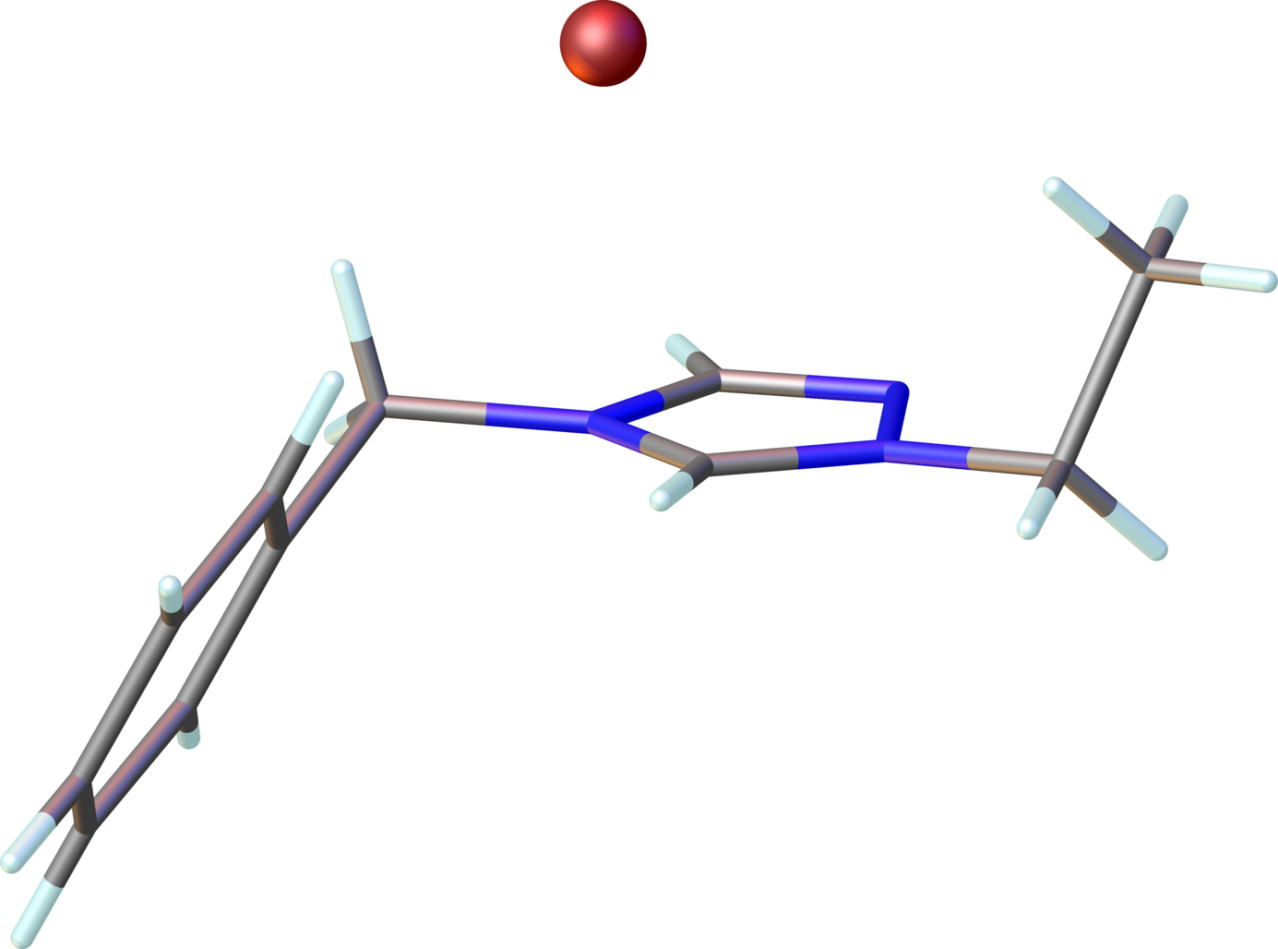
Compound **2** showing the *anti* configuration of the ethyl and benzyl wingtips relative to the N-heterocyclic ring.

**Figure 7:**
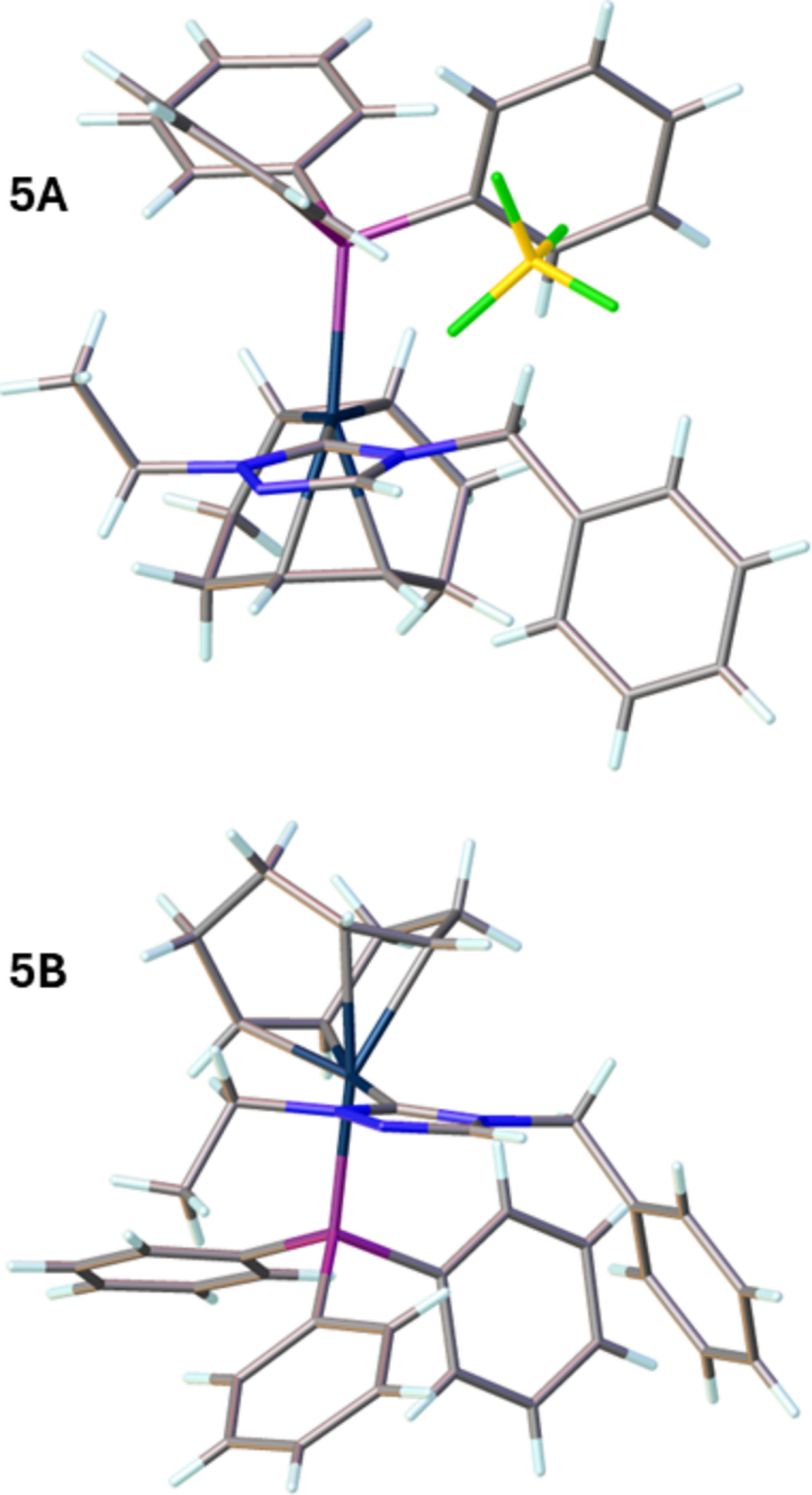
Compound **5** showing the two different cations (**5A** and **5B**) display different (*anti* (**5A**) and *syn* (**5B**) configurations of the ethyl and benzyl wingtips relative to the N-heterocyclic rings.

**Figure 8:**
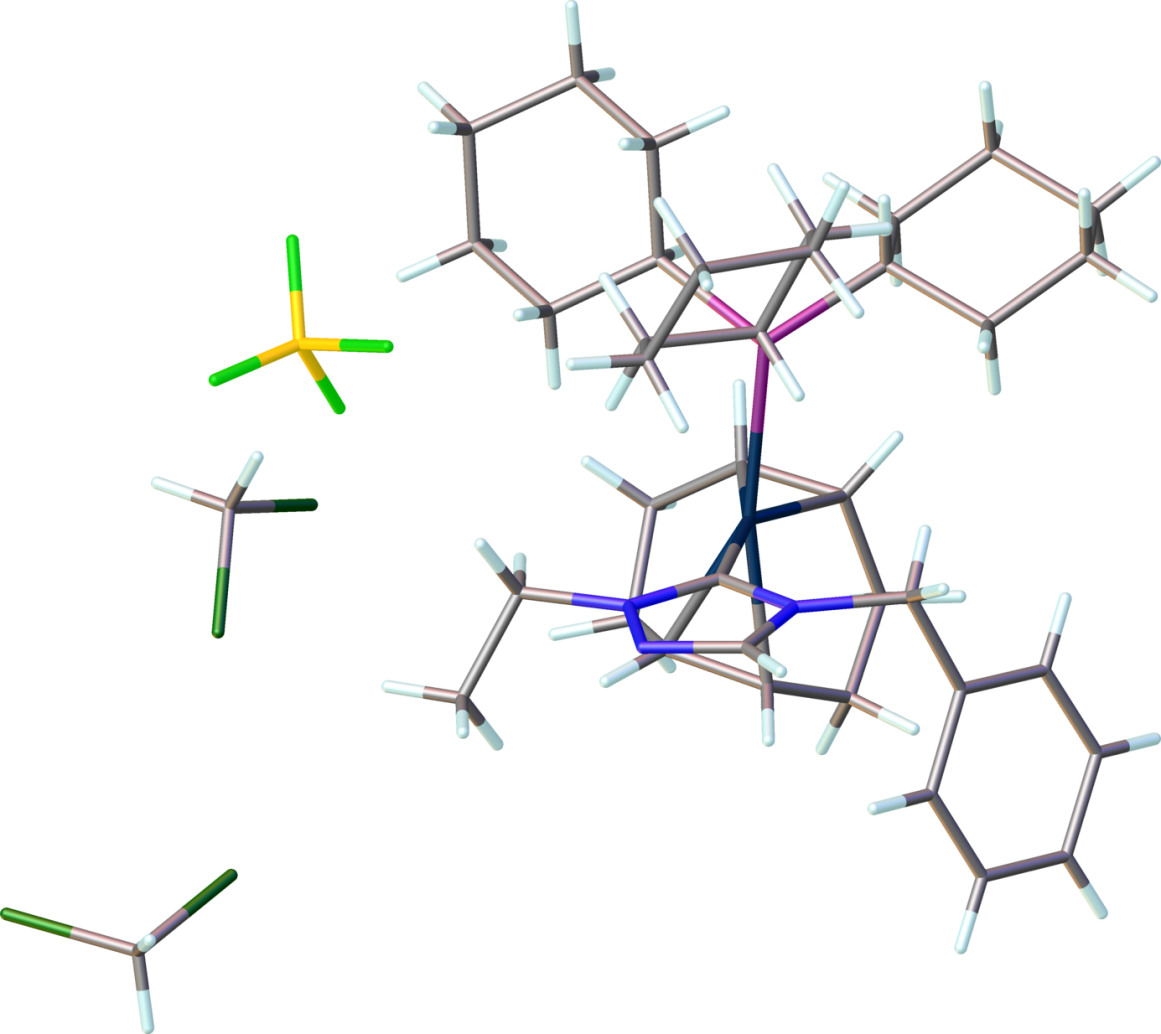
Compound **6** showing the *syn* configuration of the ethyl and benzyl wingtips relative to the N-heterocyclic ring.

In compound (**2**), the ethyl and the benzyl (wing tip) substituents on the nitro­gen atoms are in an *anti*-conformation and compound (**3**) also shows an *anti*-conformation with respect to the triazolium ring as shown in Figs. 5 ▸ and 6 ▸, respectively. In compound (**5**), the wing-tip substituents in the carbene ligands are *syn* in cation A and *anti* in cation B (Fig. 7 ▸). Fig. 7 ▸ illustrates the different conformations of the two cations. Unlike the tri­phenyl­phosphine analogue (**5**), in compound (**6**) only the *syn*-conformation of the wing tips is observed as shown in Fig. 8 ▸. The different conformations of the wingtips in various structures shows no strong preference for the *syn* or *anti* configuration of the wingtips. This is likely due to the ethyl wingtip being relatively small in size.

## 3. Supra­molecular features

**Figure 9:**
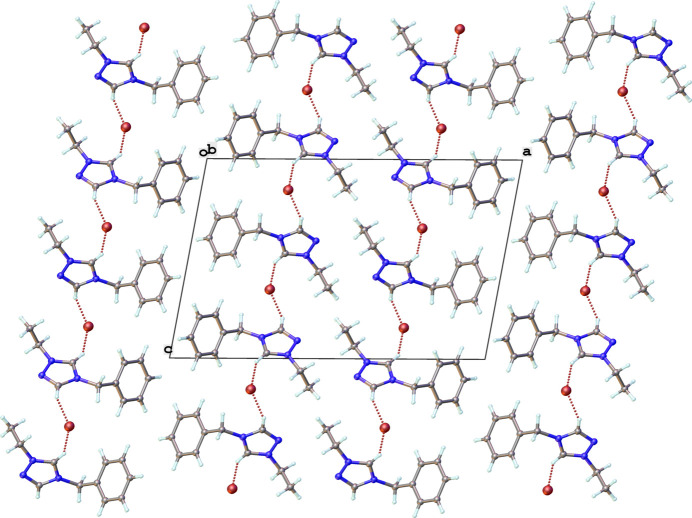
Packing diagram of **2** viewed along the *b*-axis direction. Hydrogen-bonding inter­actions are shown as dotted red lines.

**Figure 10:**
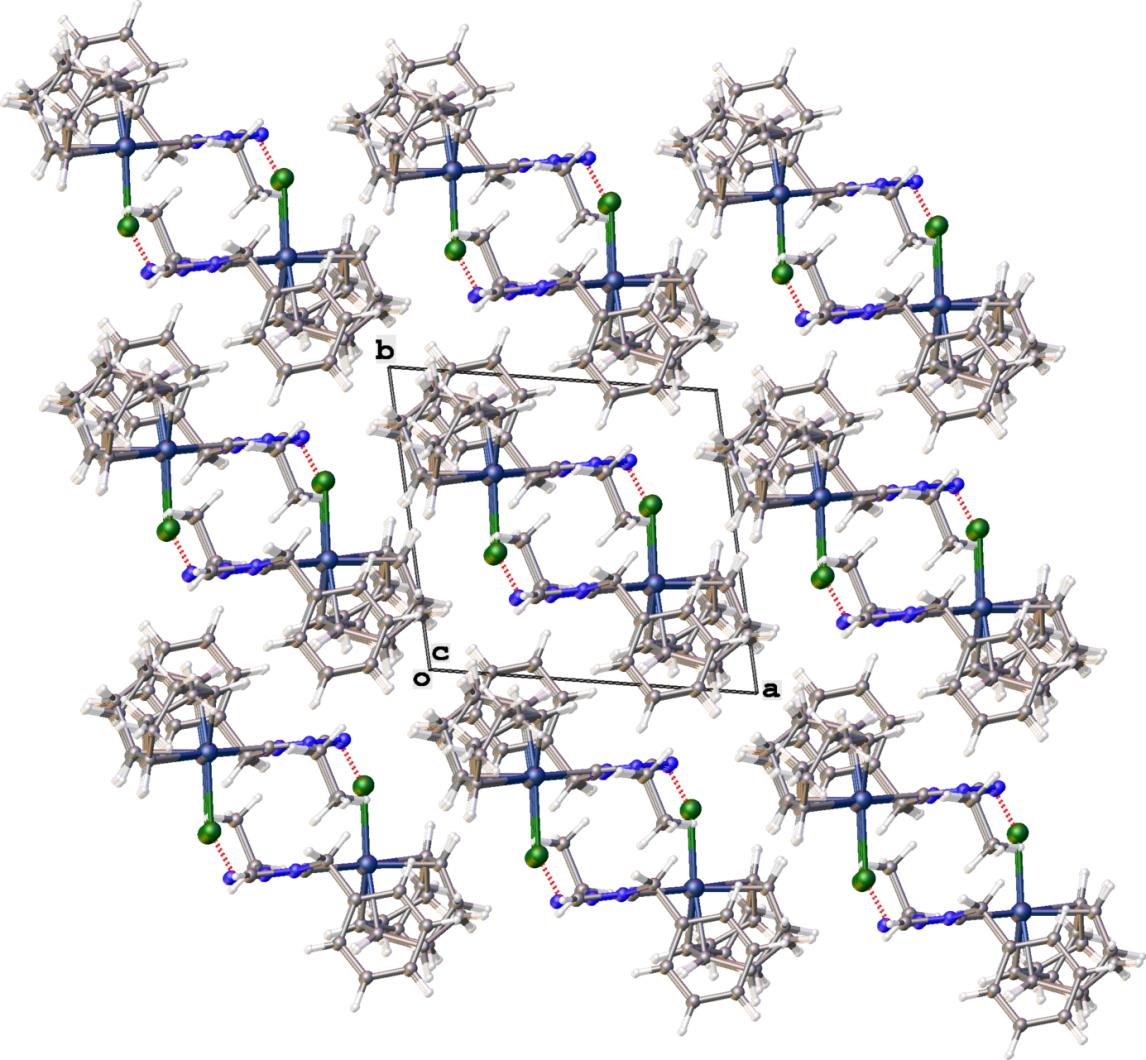
Packing diagram of **3** viewed along the *b*-axis direction. Hydrogen-bonding inter­actions are shown as dotted red lines.

**Figure 11:**
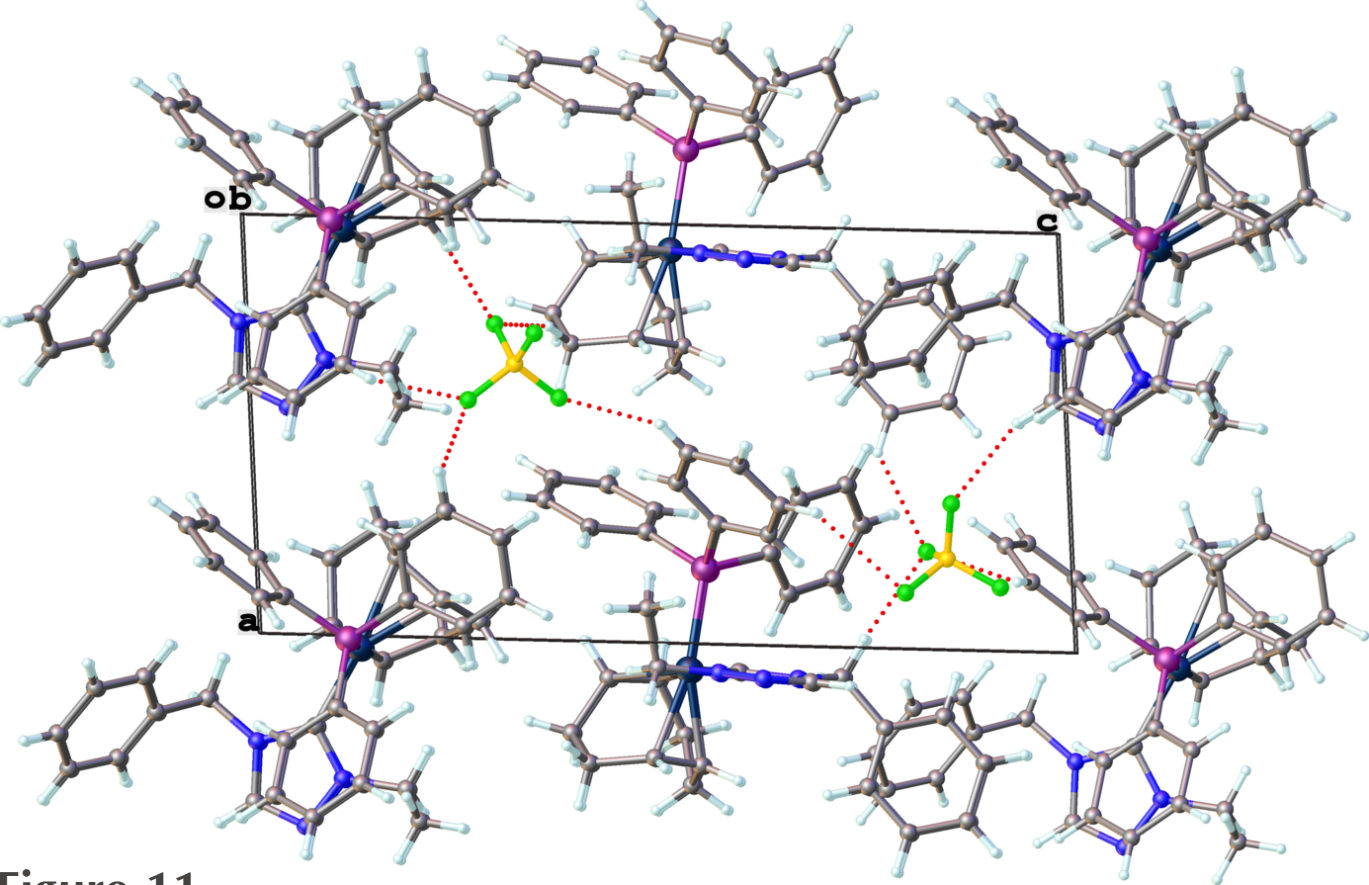
Packing diagram of **5** viewed along the *b*-axis direction. Hydrogen-bonding inter­actions are shown as dotted red lines.

Packing diagrams of the structures are shown in Figs. 9 ▸–12 ▸ ▸ ▸ with non-classical hydrogen-bonding inter­actions shown as red dotted lines and summarized in Tables 5 ▸–8 ▸ ▸ ▸. The triazolium salt (**2**), shown in Fig. 9 ▸, exhibits close contacts between the two most acidic hydrogen atoms in the structure (H1 and H2 of the triazolium ring) and the bromide anion. The C—H⋯Br^−^ hydrogen bonding inter­actions of **2**, summarized in Table 5 ▸, position the bromide ion between adjacent triazolium rings. This behavior is consistent with other observed crystal structures of 1, 2, 4-triazolium halide salts (Guino-o *et al.*, 2015 ▸; El Bakri *et al.*, 2016 ▸; Maynard *et al.*, 2023 ▸; Albert *et al.*, 2024). The neutral rhodium complex (**3**), shown in Fig. 10 ▸, crystallizes as dimer pairs with the acidic H atom of the NHC (H2) and chlorido ligand on adjacent structural units displaying a weak C—H⋯Cl hydrogen-bonding inter­action, summarized in Table 6 ▸. The ionic iridium complexes (**5** and **6**), shown in Figs. 11 ▸ and 12 ▸, respectively, display many non-classical hydrogen-bonding inter­actions, summarized in Tables 7 ▸ and 8 ▸, respectively. Most of the close contacts of the cationic complex are directed towards the tetra­fluorido­borate anion in **5**. The weak hydrogen bonds in **6** are exhibited between both between adjacent cations and between cations and the tetra­fluorido­borate anion. Potential weak hydrogen-bonding inter­actions between the di­chloro­methane solvate mol­ecule and the cation are all relatively long and are not included in Table 8 ▸. A few short non-standard hydrogen-bonding inter­actions likely occur due to disordered atoms.

## 4. Database survey

The Crystallography Open Database (Gražulis *et al.*, 2009 ▸) was queried for structures similar to those reported. A search for ‘triazolium’ and ‘salt’ yielded 54 entries in the database. A search for ‘triazol’ that included the element iridium yielded 139 entries. A search for ‘triazol’ that included the element rhodium yielded 83 entries.

## 5. Synthesis and crystallization

**Figure 13:**
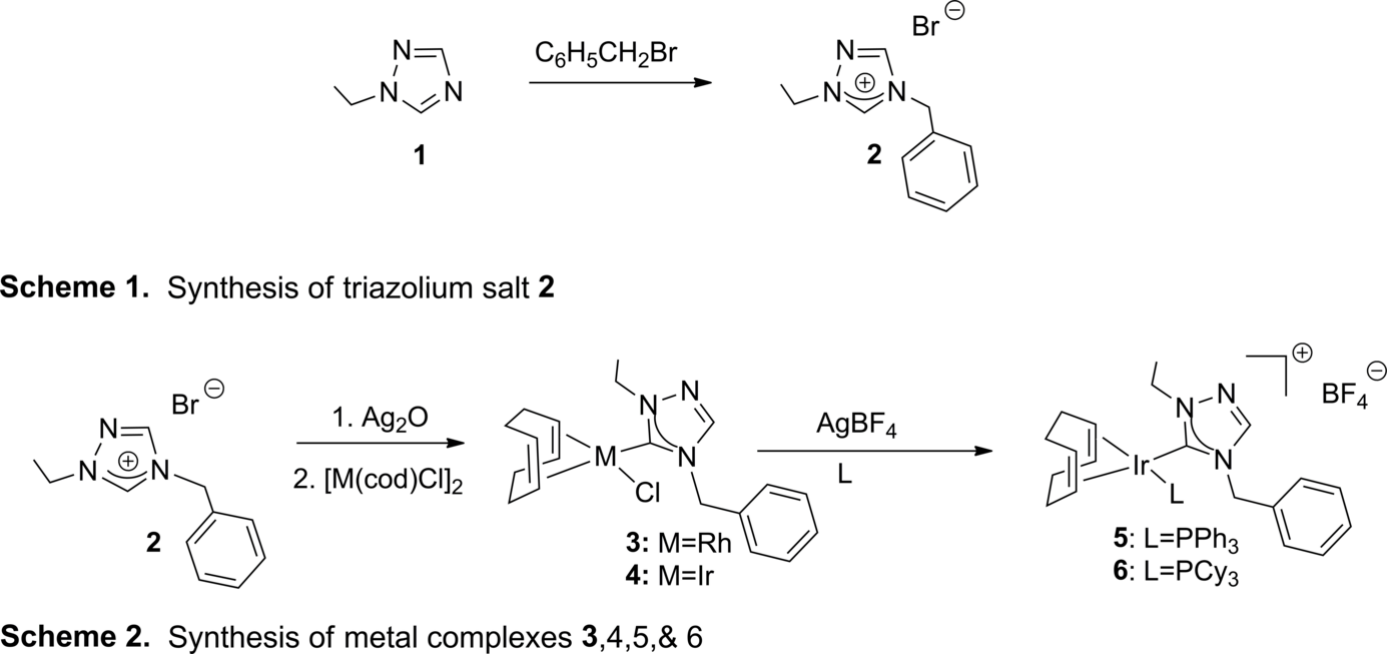
Reaction schemes for the syntheses of all compounds.

1-Ethyl-1,2,4-triazole (**1**) was purchased from Matrix Scientific. All other compounds used in the syntheses, detailed in Fig. 13 ▸, were obtained from Sigma–Aldrich and Strem and used as received; all syntheses were performed under a nitro­gen atmosphere. NMR spectra were recorded at room temperature in CDCl_3_ on a 400 MHz (operating at 100 MHz for ^13^C and 162 MHz for ^31^P) Varian spectrometer and referenced to the residual solvent peak (δ in p.p.m.). The titular series of compounds (**2**, **3**, **5**, and **6**) were crystallized by slow diffusion of pentane into a CH_2_Cl_2_ solution.

**4-Benzyl-1-ethyl-1,2,4-triazolium bromide (2):** 1-Ethyl-1,2,4-triazole (**1**) (0.410 g, 4.22 mmol) and excess α-bromo­toluene (5.000 g, 29.23 mmol) were added to toluene (15 ml), and the mixture was refluxed in the dark for 48 h. After the mixture was cooled, the white solid was filtered, washed with ether, and dried under vacuum. Yield: 0.910 g (80.4%). ^1^H NMR: CDCl_3_, δ (p.p.m.) 12.01 (*s*, 1 H, N—C_5_H—N), 8.23 (*s*, 1 H, N—C_3_—N), 7.56–7.54 (*m*, 2 H, H_arom_), 7.45–7.26 (*m*, 3 H, H_arom_), 5.78 (*s*, 2 H, CH_2_Ph), 4.56 (*q*, 2 H, CH_2_CH_3_), 1.67 (*t*, 3 H, CH_2_CH_3_). ^13^C NMR: δ 142.43 (N—CH—N), 142.24 (N—CH—N), 131.43, 130.19, 129.86, 129.45 (C_arom_), 52.41 (CH_2_Ph), 48.62 (CH_2_ of eth­yl), 14.08 (CH_3_).

**Chlorido­[(1,2,5,6-η)-cyclo­octa-1,5-diene](4-benzyl-1-ethyl-1,2,4-triazol-5-yl­idene)rhodium(I) (3):** Triazolium bromide (**2**) (0.109 g, 0.406 mmol) and Ag_2_O (0.047 g, 0.203 mmol) were stirred at room temperature in the dark for 1 h in CH_2_Cl_2_ (10 mL). The mixture was then filtered through Celite into [Rh(cod)Cl]_2_ (0.100 g, 0.203 mmol), and stirred again in the dark for 1.5 h. The resulting solution was filtered through Celite, and the solvent was removed under reduced pressure in a rotary evaporator. The yellow solid product (**3**) was dried under vacuum. Yield: 0.158 g (90%). ^1^H NMR: δ 7.68 (*s*, 1 H, N—C_3_H—N), 7.26*-*-7.38 (*m*, 5 H, H_arom_), 5.12 (*s*, 2 H, CH_2_Ph), 4.77 (*q*, 2 H, CH_2_CH_3_), 4.72 (*m*, 2 H, CH of COD), 4.64 (*m*, 2 H, CH of COD), 3.38, 3.20 (*m*, 4 H, CH_2_ of COD), 1.59 (*t*, 3 H, CH_3_ of eth­yl). ^13^C NMR: δ 185.32 (*d*, Rh—C, *J*_C—Rh_ = 50.9 Hz), 141.96 (N—C3H—N), 134.87, 129.24, 128.75, 128.50, 128.43 (C_arom_), 99.96,99.89, 99.56, 99.46 (CH of COD), 52.45 (CH_2_Ph), 47.90 (CH_2_ of eth­yl), 33.10, 32.65, 28.96, 28.64 (CH_2_ of COD), 15.44 (CH_3_ of eth­yl).

**Chlorido­[(1,2,5,6-η)-cyclo­octa-1,5-diene](4-benzyl-1-ethyl-1,2,4-triazol-5-yl­idene)iridium(I) (4):** Triazolium bromide (**2**) (0.080 g, 0.298 mmol) and Ag_2_O (0.035 g, 0.149 mmol) were stirred at room temperature in the dark for 1 h in CH_2_Cl_2_ (10 ml). The mixture was then filtered through Celite into [Ir(cod)Cl]_2_ (0.100 g, 0.149 mmol), and stirred again in the dark for 1.5 h. The resulting solution was filtered through Celite, and the solvent was removed under reduced pressure in a rotary evaporator. The bright-orange solid product (**4**) was dried under vacuum. Yield: 0.146 g (94%). ^1^H NMR: δ 7.70 (*s*, 1 H, N—C_3_H—N), 7.26–7.39 (*m*, 5 H, H_arom_), 5.72 (*s*, 2 H, CH_2_Ph), 4.74 (*q*, 2 H, CH_2_CH_3_), 4.71 (*m*, 2 H, CH of COD), 4.64 (*m*, 2 H, CH of COD), 3.10–2.81 (*m*, 4 H, CH_2_ of COD), 1.56 (*t*, 3 H, CH_3_ of eth­yl). ^13^C NMR: δ 182.61 (Ir—C), 141.75 (N—C_3_H—N), 134.72, 129.21, 128.73, 128.45 (C_arom_), 86.86,86.32 (CH of COD), 52.76 (CH_2_Ph), 47.68 (CH_2_ of eth­yl), 33.82, 33.14, 29.73, 29.10 (CH_2_ of COD), 15.41 (CH_3_ of eth­yl).

**[(1,2,5,6-η)-Cyclo­octa-1,5-diene](4-benzyl-1-ethyl-1,2,4-tri­a­zol-5-yl­idene)(tri­phenyl­phosphane)iridium(I) tetra­fluorido­borate (5):** Tri­phenyl­phosphane (0.052 g, 0.197 mmol) and AgBF_4_ (0.038 g, 0.197 mmol) were added to (**4**) (0.103 g, 0.197 mmol) in CH_2_Cl_2_ (15 mL). The solution was stirred in the dark for 1.5 h. The resulting mixture was filtered through Celite, and the solvent was removed under reduced pressure. The bright-red solid product (**5**) was dried under vacuum. Yield: 0.165 g (100%). ^1^H NMR: δ 7.91 (*s*, 1 H, N—C_3_H—N), 7.53–7.01 (*m*, 20 H, H_arom_), 5.53 (*s*, 2 H, CH_2_Ph), 4.74 (*q*, 2 H, CH_2_CH_3_), 4.71 (*m*, 2 H, CH of COD), 4.51 (*m*, 2 H, CH of COD), 2.43–2.01 (*m*, 4 H, CH_2_ of COD), 1.56 (*t*, 3 H, CH_3_ of Eth­yl). ^13^C NMR: δ 178.26 (Ir—C), 143.80 (N—C_3_H—N), 134.03-128.27 (C_arom_), 87.89, 87.76, 86.64, 86.53 (CH of COD), 52.07 (CH_2_Ph), 47.97 (CH_2_ of eth­yl), 31.56, 31.06, 30.60, 30.12 (CH_2_ of COD), 13.84 (CH_3_ of eth­yl). ^31^P NMR: δ 17.37.

**[(1,2,5,6-η)-Cyclo­octa-1,5-diene](4-benzyl-1-ethyl-1,2,4-tri­azol-5-yl­idene)(tri­cyclo­hexyl­phosphane)iridium(I) tetra­fluorido­borate (6):** Tri­cyclo­hexyl­phosphane (0.055 g, 0.197 mmol) and AgBF_4_ (0.038 g, 0.197 mmol) were added to (**4**) (0.103 g, 0.197 mmol) in CH_2_Cl_2_ (15 mL). The solution was stirred in the dark for 1.5 h. The resulting mixture was filtered through Celite, and the solvent was removed under reduced pressure. The bright-orange solid product (**6**) was dried under vacuum. Yield: 0.168 g (100%). ^1^H NMR: δ 8.32 (*s*, 1 H, N—C_3_H—N), 7.43–7.26 (*m*, 5 H, H_arom_), 5.54 (*s*, 2 H, CH_2_Ph), 4.57 (*q*, 2 H, CH_2_CH_3_), 4.71 (*m*, 2 H, CH of COD), 4.22 (*m*, 2 H, CH of COD), 2.25-2.19 (*m*, 4 H, CH_2_ of COD), 1.84–1.14 (*m*, 36 H, CH_3_ of ethyl and CH/CH_2_ of PCy_3_). ^13^C NMR: δ 179.54 (Ir—C), 144.58 (N—C_3_H—N), 129.42-127.75 (C_arom_), 82.04, 81.94, 79.27, 77.60 (CH of COD), 52.32 (CH_2_Ph), 48.17 (CH_2_ of eth­yl), 37.08, 36.85, 35.03 (CH of PCy_3_), 31.89, 31.86, 31.81, 31.77 (CH_2_ of COD), 29.95 – 25.76 (CH_2_ of PCy_3_), 14.23 (CH_3_ of eth­yl). ^31^P NMR: δ 15.51.

## 6. Refinement

Crystal data, data collection and structure refinement details are summarized in Table 9 ▸. The non-H atoms were refined anisotropically and hydrogen atoms were refined using a riding model. Refinement of **3** and **6** included several disordered atoms/mol­ecules (COD ligand in **3** and COD ligand, tetra­fluorido­borate anion, and di­chloro­methane solvate in **6**). In **6**, one di­chloro­methane solvate mol­ecule lies on a crystallographic center of symmetry and was modeled using a PART −1 card in *SHELXL* and as half occupancy.

## Supplementary Material

Crystal structure: contains datablock(s) 2, 3, 5, 6. DOI: 10.1107/S2056989025002671/hb8128sup1.cif

Structure factors: contains datablock(s) 2. DOI: 10.1107/S2056989025002671/hb81282sup6.hkl

Structure factors: contains datablock(s) 3. DOI: 10.1107/S2056989025002671/hb81283sup7.hkl

Structure factors: contains datablock(s) 5. DOI: 10.1107/S2056989025002671/hb81285sup8.hkl

Structure factors: contains datablock(s) 6. DOI: 10.1107/S2056989025002671/hb81286sup9.hkl

Supporting information file. DOI: 10.1107/S2056989025002671/hb81282sup6.cml

CCDC references: 2433608, 2433607, 2433606, 2433605

Additional supporting information:  crystallographic information; 3D view; checkCIF report

## Figures and Tables

**Figure 6:**
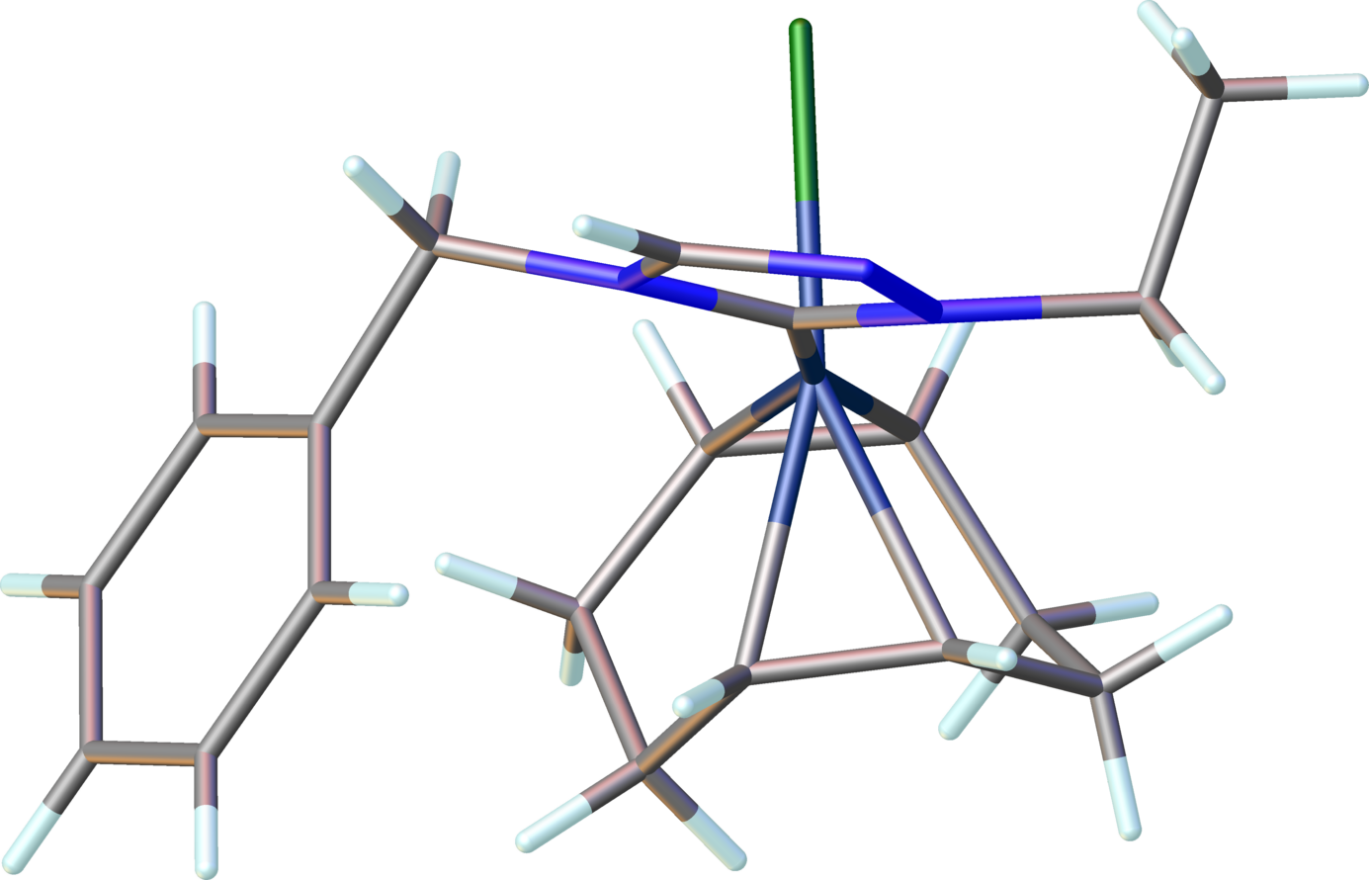
Compound **3** showing the *anti* configuration of the ethyl and benzyl wingtips relative to the N-heterocyclic ring.

**Figure 12:**
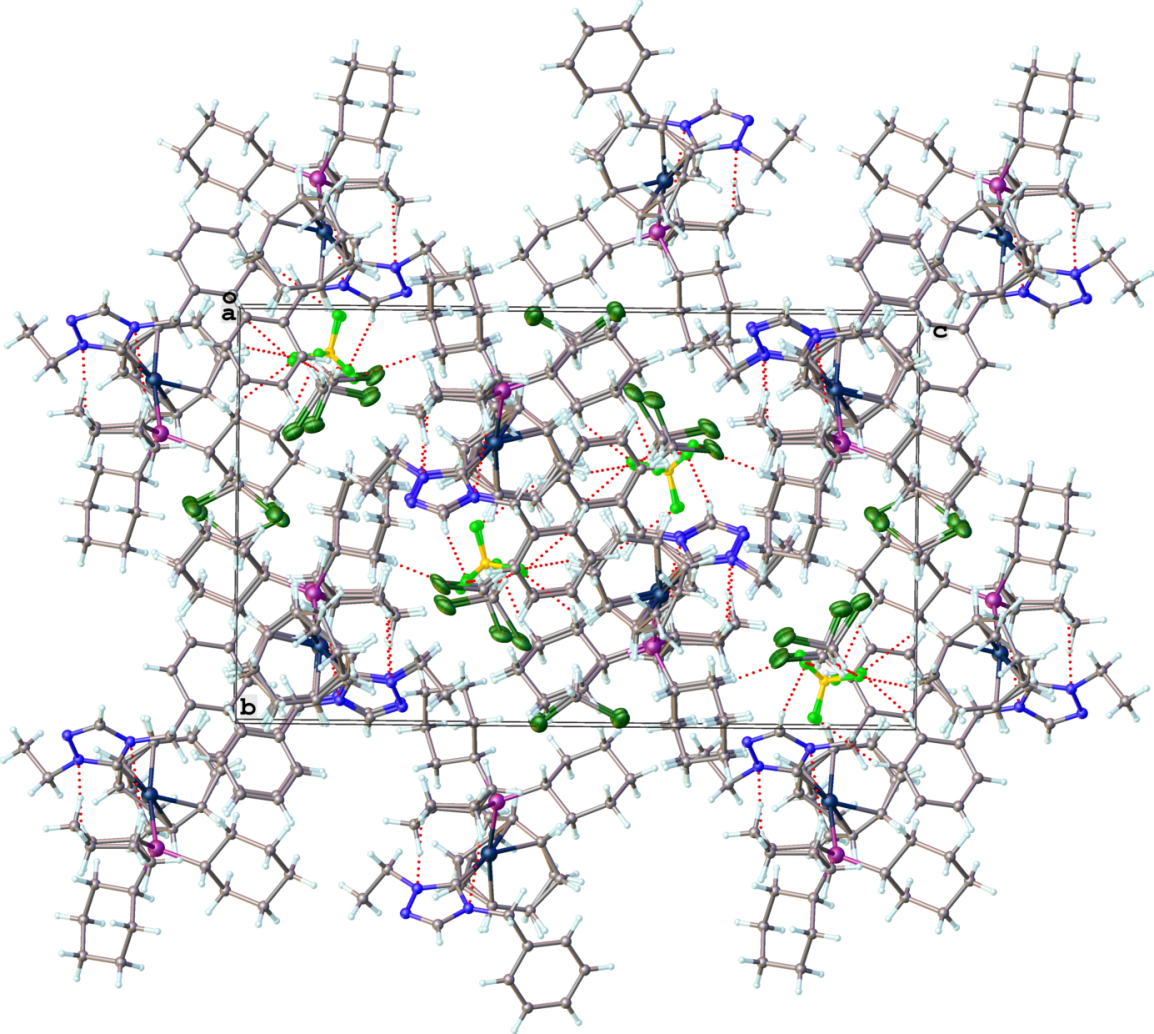
Packing diagram of **6** viewed along the *a*-axis direction. Hydrogen-bonding inter­actions are shown as dotted red lines.

**Table 1:** Selected geometric parameters (Å, °) for **2**[Chem scheme1]

N1—C1	1.315 (3)	N3—C1	1.339 (3)
			
N1—C1—N3	107.1 (2)		

**Table 2:** Selected geometric parameters (Å, °) for **3**[Chem scheme1]

N1—C1	1.343 (3)	Rh1—Cl1	2.3960 (6)
N3—C1	1.367 (3)		
			
N1—C1—N3	102.7 (2)	C1—Rh1—Cl1	89.14 (7)

**Table 3:** Selected geometric parameters (Å, °) for **5**[Chem scheme1]

N1—C1	1.336 (8)	N1′—C1′	1.340 (8)
N3—C1	1.354 (8)	N3′—C1′	1.380 (8)
Ir1—C1	2.039 (6)	Ir1′—C1′	2.029 (6)
			
N1—C1—N3	103.8 (5)	N1′—C1′—N3′	102.7 (5)
C1—Ir1—P1	93.14 (17)	C1′—Ir1′—P1′	94.64 (18)

**Table 4:** Selected geometric parameters (Å, °) for **6**[Chem scheme1]

N1—C1	1.352 (5)	Ir1—C1	2.034 (4)
N3—C1	1.369 (5)		
			
N1—C1—N3	102.2 (3)	C1—Ir1—P1	93.42 (10)

**Table 5:** Hydrogen-bond geometry (Å, °) for **2**[Chem scheme1]

*D*—H⋯*A*	*D*—H	H⋯*A*	*D*⋯*A*	*D*—H⋯*A*
C1—H1⋯Br1^i^	0.95	2.70	3.528 (3)	146
C2—H2⋯Br1^ii^	0.95	2.71	3.603 (3)	157

Symmetry codes: (i) 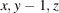; (ii) 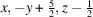.

**Table 6:** Hydrogen-bond geometry (Å, °) for **3**[Chem scheme1]

*D*—H⋯*A*	*D*—H	H⋯*A*	*D*⋯*A*	*D*—H⋯*A*
C2—H2⋯Cl1^i^	0.95	2.64	3.461 (3)	145

Symmetry code: (i) 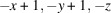.

**Table 7:** Hydrogen-bond geometry (Å, °) for **5**[Chem scheme1]

*D*—H⋯*A*	*D*—H	H⋯*A*	*D*⋯*A*	*D*—H⋯*A*
C5—H5*A*⋯F2	0.99	2.52	3.488 (8)	167
C8—H8⋯F2^i^	0.95	2.51	3.262 (10)	137
C14—H14⋯F4′	0.95	2.40	3.250 (8)	149
C16—H16⋯F3	0.95	2.74	3.487 (9)	136
C21—H21⋯F3′^ii^	0.95	2.52	3.413 (8)	157
C22—H22⋯F4′^ii^	0.95	2.62	3.396 (8)	139
C33—H33*A*⋯F1′^iii^	0.99	2.51	3.378 (9)	146
C37—H37*B*⋯F2′^i^	0.99	2.38	3.313 (9)	158
C2′—H2′⋯F1^iv^	0.95	2.37	3.274 (9)	160
C16′—H16′⋯F2^v^	0.95	2.59	3.388 (7)	142
C20′—H20′⋯F3′^vi^	0.95	2.71	3.496 (8)	141
C28′—H28′⋯F3′	0.95	2.45	3.335 (8)	154
C32′—H32*D*⋯F2′^ii^	0.99	2.44	3.425 (7)	176
C37′—H37*C*⋯F4^vii^	0.99	2.51	3.375 (8)	146

Symmetry codes: (i) 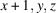; (ii) 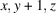; (iii) 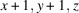; (iv) 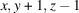; (v) 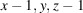; (vi) 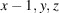; (vii) 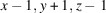.

**Table 8:** Hydrogen-bond geometry (Å, °) for **6**[Chem scheme1]

*D*—H⋯*A*	*D*—H	H⋯*A*	*D*⋯*A*	*D*—H⋯*A*
C2—H2⋯F2^i^	0.95	2.25	3.140 (6)	155
C7—H7⋯F3^ii^	0.95	2.39	3.182 (7)	141
C8—H8⋯F4^ii^	0.95	2.61	3.424 (9)	144
C13—H13*A*⋯F1*	0.99	2.47	3.263 (13)	137
C22—H22*B*⋯F2*^ii^	0.99	2.55	3.340 (11)	137
C23—H23*B*⋯F3^ii^	0.99	2.44	3.244 (6)	139
C24—H24⋯N3	1.00	2.67	3.497 (5)	140
C29—H29*B*⋯N1	0.99	2.55	3.383 (5)	142
C33*—H33*C*⋯F3*^ii^	0.99	2.06	2.907 (11)	143
C38—H38*A*⋯F1	0.99	1.98	2.93 (2)	158
C38*—H38*D*⋯F3*	0.99	2.44	3.37 (4)	155

Symmetry codes: (i) 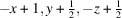; (ii) 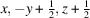.

**Table 9:** Experimental details

	**2**	**3**	**5**	**6**
Crystal data				
Chemical formula	C_11_H_14_N_3_^+^·Br^−^	[RhCl(C_8_H_12_)(C_11_H_13_N_3_)]	[Ir(C_8_H_12_)(C_11_H_13_N_3_)(C_18_H_15_P)]BF_4_	[Ir(C_8_H_12_)(C_11_H_13_N_3_)(C_18_H_33_P)]BF_4_·1.5CH_2_Cl_2_
*M*_r_	268.16	433.78	836.70	982.23
Crystal system, space group	Monoclinic, *P*2_1_/*c*	Triclinic, *P* 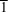	Triclinic, *P*1	Monoclinic, *P*2_1_/*c*
Temperature (K)	100	100	100	100
*a*, *b*, *c* (Å)	19.6908 (10), 4.7431 (2), 12.6482 (5)	10.1404 (2), 10.2958 (2), 10.3306 (2)	9.47197 (15), 9.50712 (15), 18.7104 (3)	12.1281 (2), 14.4399 (2), 23.7057 (3)
α, β, γ (°)	90, 100.400 (4), 90	116.818 (2), 103.489 (2), 93.997 (2)	79.8203 (14), 86.1222 (13), 89.3859 (13)	90, 92.016 (1), 90
*V* (Å^3^)	1161.88 (9)	916.78 (4)	1654.57 (5)	4148.98 (10)
*Z*	4	2	2	4
Radiation type	Mo *K*α	Mo *K*α	Mo *K*α	Mo *K*α
μ (mm^−1^)	3.51	1.08	4.14	3.50
Crystal size (mm)	0.38 × 0.19 × 0.02	0.5 × 0.34 × 0.23	0.31 × 0.27 × 0.23	0.27 × 0.1 × 0.01

Data collection				
Diffractometer	Rigaku XtaLAB Synergy-S	Rigaku XtaLAB Synergy-S	Rigaku XtaLAB Synergy-S	Rigaku XtaLAB Synergy-S
Absorption correction	Multi-scan (SCALE3 ABSPACK; Rigaku OD, 2024 ▸)	Multi-scan (SCALE3 ABSPACK; Rigaku OD, 2024 ▸)	Multi-scan (SCALE3 ABSPACK; Rigaku OD, 2024 ▸)	Multi-scan (SCALE3 ABSPACK; Rigaku OD, 2024 ▸)
*T*_min_, *T*_max_	0.641, 1.000	0.740, 1.000	0.857, 1.000	0.642, 1.000
No. of measured, independent and observed [*I* > 2σ(*I*)] reflections	22182, 2883, 2356	28369, 4565, 4281	50908, 15249, 14450	88352, 10279, 8797
*R*_int_	0.071	0.048	0.045	0.051
(sin θ/λ)_max_ (Å^−1^)	0.667	0.667	0.667	0.667

Refinement				
*R*[*F*^2^ > 2σ(*F*^2^)], *wR*(*F*^2^), *S*	0.038, 0.074, 1.05	0.031, 0.081, 1.04	0.027, 0.062, 1.05	0.036, 0.078, 1.05
No. of reflections	2883	4565	15249	10279
No. of parameters	137	255	849	575
No. of restraints	0	76	3	309
H-atom treatment	H-atom parameters constrained	H-atom parameters constrained	H-atom parameters constrained	H-atom parameters constrained
Δρ_max_, Δρ_min_ (e Å^−3^)	1.40, −0.58	1.52, −0.82	3.38, −1.12	1.90, −1.29
Absolute structure	–	–	Flack *x* determined using 6303 quotients [(*I*^+^)−(*I*^−^)]/[(*I*^+^)+(*I*^−^)] (Parsons *et al.*, 2013 ▸)	–
Absolute structure parameter	–	–	−0.008 (3)	–

Computer programs: *CrysAlis PRO* (Rigaku OD, 2024 ▸), SHELXT2018/2 (Sheldrick, 2015*a* ▸), *SHELXL2018/3* (Sheldrick, 2015*b* ▸), *OLEX2* (Dolomanov *et al.*, 2009 ▸) and *publCIF* (Westrip, 2010 ▸).

## supplementary crystallographic information

### 4-Benzyl-1-ethyl-1,2,4-triazolium bromide (2) Crystal data

**Table:**

C_11_H_14_N_3_^+^·Br^−^	*F*(000) = 544
*M**_r_* = 268.16	*D*_x_ = 1.533 Mg m^−^^3^
Monoclinic, *P*2_1_/*c*	Mo *K*α radiation, λ = 0.71073 Å
*a* = 19.6908 (10) Å	Cell parameters from 7642 reflections
*b* = 4.7431 (2) Å	θ = 2.1–28.2°
*c* = 12.6482 (5) Å	µ = 3.51 mm^−^^1^
β = 100.400 (4)°	*T* = 100 K
*V* = 1161.88 (9) Å^3^	Plate, colourless
*Z* = 4	0.38 × 0.19 × 0.02 mm

### 4-Benzyl-1-ethyl-1,2,4-triazolium bromide (2) Data collection

**Table:**

Rigaku XtaLAB Synergy-S diffractometer	2356 reflections with *I* > 2σ(*I*)
Detector resolution: 10.0 pixels mm^-1^	*R*_int_ = 0.071
ω scans	θ_max_ = 28.3°, θ_min_ = 2.1°
Absorption correction: multi-scan (SCALE3 ABSPACK; Rigaku OD, 2024)	*h* = −26→26
*T*_min_ = 0.641, *T*_max_ = 1.000	*k* = −5→6
22182 measured reflections	*l* = −16→16
2883 independent reflections	

### 4-Benzyl-1-ethyl-1,2,4-triazolium bromide (2) Refinement

**Table:**

Refinement on *F*^2^	0 restraints
Least-squares matrix: full	Hydrogen site location: inferred from neighbouring sites
*R*[*F*^2^ > 2σ(*F*^2^)] = 0.038	H-atom parameters constrained
*wR*(*F*^2^) = 0.074	*w* = 1/[σ^2^(*F*_o_^2^) + (0.0242*P*)^2^ + 1.9068*P*] where *P* = (*F*_o_^2^ + 2*F*_c_^2^)/3
*S* = 1.05	(Δ/σ)_max_ = 0.001
2883 reflections	Δρ_max_ = 1.40 e Å^−^^3^
137 parameters	Δρ_min_ = −0.58 e Å^−^^3^

### 4-Benzyl-1-ethyl-1,2,4-triazolium bromide (2) Special details

**Table:**

Geometry. All esds (except the esd in the dihedral angle between two l.s. planes) are estimated using the full covariance matrix. The cell esds are taken into account individually in the estimation of esds in distances, angles and torsion angles; correlations between esds in cell parameters are only used when they are defined by crystal symmetry. An approximate (isotropic) treatment of cell esds is used for estimating esds involving l.s. planes.

### 4-Benzyl-1-ethyl-1,2,4-triazolium bromide (2) Fractional atomic coordinates and isotropic or equivalent isotropic displacement parameters (Å^2^)

**Table:**

	*x*	*y*	*z*	*U*_iso_*/*U*_eq_	
Br1	0.27593 (2)	1.08223 (5)	0.65553 (2)	0.01623 (9)	
N1	0.37156 (11)	0.6097 (5)	0.48270 (16)	0.0137 (4)	
N2	0.39368 (12)	0.7913 (5)	0.41081 (18)	0.0187 (5)	
N3	0.27987 (11)	0.7978 (4)	0.39526 (17)	0.0133 (5)	
C1	0.30403 (14)	0.6171 (5)	0.4744 (2)	0.0135 (5)	
H1	0.277417	0.513698	0.516732	0.016*	
C2	0.33630 (14)	0.8980 (6)	0.3585 (2)	0.0174 (6)	
H2	0.334033	1.030051	0.301532	0.021*	
C3	0.42177 (14)	0.4623 (6)	0.5637 (2)	0.0177 (6)	
H3A	0.455642	0.362518	0.528078	0.021*	
H3B	0.397652	0.320393	0.600822	0.021*	
C4	0.45923 (16)	0.6703 (6)	0.6452 (2)	0.0228 (6)	
H4A	0.482324	0.812772	0.608167	0.034*	
H4B	0.493647	0.569756	0.697392	0.034*	
H4C	0.425887	0.762499	0.682781	0.034*	
C5	0.20775 (14)	0.8951 (6)	0.3593 (2)	0.0170 (6)	
H5A	0.201490	0.950344	0.282680	0.020*	
H5B	0.199664	1.064231	0.401177	0.020*	
C6	0.15510 (14)	0.6741 (5)	0.3724 (2)	0.0145 (5)	
C7	0.13942 (14)	0.6194 (6)	0.4741 (2)	0.0182 (6)	
H7	0.163016	0.718389	0.535103	0.022*	
C8	0.08942 (15)	0.4206 (7)	0.4862 (2)	0.0225 (6)	
H8	0.079519	0.382201	0.555547	0.027*	
C9	0.05407 (14)	0.2786 (6)	0.3975 (2)	0.0207 (6)	
H9	0.019573	0.144551	0.405767	0.025*	
C10	0.06910 (14)	0.3324 (6)	0.2964 (2)	0.0195 (6)	
H10	0.044887	0.234676	0.235544	0.023*	
C11	0.11936 (14)	0.5285 (6)	0.2838 (2)	0.0176 (6)	
H11	0.129458	0.563655	0.214295	0.021*	

### 4-Benzyl-1-ethyl-1,2,4-triazolium bromide (2) Atomic displacement parameters (Å^2^)

**Table:**

	*U*^11^	*U*^22^	*U*^33^	*U*^12^	*U*^13^	*U*^23^
Br1	0.02611 (15)	0.01224 (13)	0.01118 (12)	−0.00239 (12)	0.00559 (9)	−0.00042 (11)
N1	0.0198 (12)	0.0125 (11)	0.0091 (10)	−0.0007 (9)	0.0036 (9)	0.0015 (9)
N2	0.0229 (13)	0.0208 (12)	0.0131 (11)	−0.0032 (10)	0.0052 (10)	0.0042 (10)
N3	0.0194 (12)	0.0101 (11)	0.0107 (11)	0.0003 (9)	0.0036 (9)	−0.0002 (9)
C1	0.0198 (13)	0.0098 (12)	0.0114 (12)	0.0008 (11)	0.0041 (10)	−0.0013 (10)
C2	0.0255 (15)	0.0161 (13)	0.0106 (12)	−0.0040 (12)	0.0037 (11)	0.0018 (11)
C3	0.0193 (14)	0.0159 (14)	0.0178 (13)	0.0031 (11)	0.0032 (11)	0.0063 (11)
C4	0.0247 (16)	0.0265 (16)	0.0161 (14)	0.0026 (12)	0.0010 (12)	0.0029 (12)
C5	0.0181 (14)	0.0160 (13)	0.0155 (13)	0.0010 (11)	−0.0013 (10)	0.0020 (11)
C6	0.0169 (14)	0.0135 (13)	0.0130 (13)	0.0058 (10)	0.0028 (11)	0.0006 (10)
C7	0.0211 (14)	0.0189 (14)	0.0149 (13)	0.0032 (12)	0.0038 (11)	−0.0015 (11)
C8	0.0237 (15)	0.0284 (16)	0.0168 (13)	0.0026 (14)	0.0070 (11)	0.0044 (13)
C9	0.0178 (14)	0.0166 (14)	0.0282 (16)	0.0005 (12)	0.0054 (12)	0.0026 (12)
C10	0.0179 (14)	0.0183 (14)	0.0212 (14)	0.0020 (11)	0.0009 (12)	−0.0039 (11)
C11	0.0193 (14)	0.0187 (14)	0.0155 (13)	0.0033 (11)	0.0046 (11)	0.0016 (11)

### 4-Benzyl-1-ethyl-1,2,4-triazolium bromide (2) Geometric parameters (Å, º)

**Table:**

N1—N2	1.379 (3)	C5—H5A	0.9900
N1—C1	1.315 (3)	C5—H5B	0.9900
N1—C3	1.466 (3)	C5—C6	1.504 (4)
N2—C2	1.304 (4)	C6—C7	1.401 (4)
N3—C1	1.339 (3)	C6—C11	1.394 (4)
N3—C2	1.365 (3)	C7—H7	0.9500
N3—C5	1.484 (3)	C7—C8	1.391 (4)
C1—H1	0.9500	C8—H8	0.9500
C2—H2	0.9500	C8—C9	1.384 (4)
C3—H3A	0.9900	C9—H9	0.9500
C3—H3B	0.9900	C9—C10	1.387 (4)
C3—C4	1.519 (4)	C10—H10	0.9500
C4—H4A	0.9800	C10—C11	1.388 (4)
C4—H4B	0.9800	C11—H11	0.9500
C4—H4C	0.9800		
			
N2—N1—C3	120.3 (2)	N3—C5—H5A	109.0
C1—N1—N2	111.5 (2)	N3—C5—H5B	109.0
C1—N1—C3	127.8 (2)	N3—C5—C6	113.0 (2)
C2—N2—N1	103.2 (2)	H5A—C5—H5B	107.8
C1—N3—C2	106.0 (2)	C6—C5—H5A	109.0
C1—N3—C5	128.3 (2)	C6—C5—H5B	109.0
C2—N3—C5	125.5 (2)	C7—C6—C5	120.0 (2)
N1—C1—N3	107.1 (2)	C11—C6—C5	121.0 (2)
N1—C1—H1	126.4	C11—C6—C7	118.9 (3)
N3—C1—H1	126.4	C6—C7—H7	119.9
N2—C2—N3	112.1 (2)	C8—C7—C6	120.3 (3)
N2—C2—H2	123.9	C8—C7—H7	119.9
N3—C2—H2	123.9	C7—C8—H8	119.9
N1—C3—H3A	109.6	C9—C8—C7	120.2 (3)
N1—C3—H3B	109.6	C9—C8—H8	119.9
N1—C3—C4	110.4 (2)	C8—C9—H9	120.1
H3A—C3—H3B	108.1	C8—C9—C10	119.9 (3)
C4—C3—H3A	109.6	C10—C9—H9	120.1
C4—C3—H3B	109.6	C9—C10—H10	119.8
C3—C4—H4A	109.5	C9—C10—C11	120.3 (3)
C3—C4—H4B	109.5	C11—C10—H10	119.8
C3—C4—H4C	109.5	C6—C11—H11	119.8
H4A—C4—H4B	109.5	C10—C11—C6	120.4 (3)
H4A—C4—H4C	109.5	C10—C11—H11	119.8
H4B—C4—H4C	109.5		
			
N1—N2—C2—N3	1.3 (3)	C3—N1—C1—N3	174.2 (2)
N2—N1—C1—N3	1.6 (3)	C5—N3—C1—N1	−175.4 (2)
N2—N1—C3—C4	67.8 (3)	C5—N3—C2—N2	174.5 (2)
N3—C5—C6—C7	77.2 (3)	C5—C6—C7—C8	178.3 (3)
N3—C5—C6—C11	−105.3 (3)	C5—C6—C11—C10	−177.6 (2)
C1—N1—N2—C2	−1.8 (3)	C6—C7—C8—C9	−1.0 (4)
C1—N1—C3—C4	−104.3 (3)	C7—C6—C11—C10	0.0 (4)
C1—N3—C2—N2	−0.4 (3)	C7—C8—C9—C10	0.7 (4)
C1—N3—C5—C6	−32.3 (4)	C8—C9—C10—C11	−0.1 (4)
C2—N3—C1—N1	−0.7 (3)	C9—C10—C11—C6	−0.2 (4)
C2—N3—C5—C6	153.9 (2)	C11—C6—C7—C8	0.6 (4)
C3—N1—N2—C2	−175.0 (2)		

### 4-Benzyl-1-ethyl-1,2,4-triazolium bromide (2) Hydrogen-bond geometry (Å, º)

**Table:**

*D*—H···*A*	*D*—H	H···*A*	*D*···*A*	*D*—H···*A*
C1—H1···Br1^i^	0.95	2.70	3.528 (3)	146
C2—H2···Br1^ii^	0.95	2.71	3.603 (3)	157

Symmetry codes: (i) *x*, *y*−1, *z*; (ii) *x*, −*y*+5/2, *z*−1/2.

### (4-Benzyl-1-ethyl-1,2,4-triazol-5-ylidene)chlorido[(1,2,5,6-η)-cycloocta-1,5-diene]rhodium(I) (3) Crystal data

**Table:**

[RhCl(C_8_H_12_)(C_11_H_13_N_3_)]	*Z* = 2
*M**_r_* = 433.78	*F*(000) = 444
Triclinic, *P*1	*D*_x_ = 1.571 Mg m^−^^3^
*a* = 10.1404 (2) Å	Mo *K*α radiation, λ = 0.71073 Å
*b* = 10.2958 (2) Å	Cell parameters from 20452 reflections
*c* = 10.3306 (2) Å	θ = 2.2–28.2°
α = 116.818 (2)°	µ = 1.08 mm^−^^1^
β = 103.489 (2)°	*T* = 100 K
γ = 93.997 (2)°	Block, yellow
*V* = 916.78 (4) Å^3^	0.5 × 0.34 × 0.23 mm

### (4-Benzyl-1-ethyl-1,2,4-triazol-5-ylidene)chlorido[(1,2,5,6-η)-cycloocta-1,5-diene]rhodium(I) (3) Data collection

**Table:**

Rigaku XtaLAB Synergy-S diffractometer	4281 reflections with *I* > 2σ(*I*)
Detector resolution: 10.0 pixels mm^-1^	*R*_int_ = 0.048
ω scans	θ_max_ = 28.3°, θ_min_ = 2.1°
Absorption correction: multi-scan (SCALE3 ABSPACK; Rigaku OD, 2024)	*h* = −13→13
*T*_min_ = 0.740, *T*_max_ = 1.000	*k* = −13→13
28369 measured reflections	*l* = −13→13
4565 independent reflections	

### (4-Benzyl-1-ethyl-1,2,4-triazol-5-ylidene)chlorido[(1,2,5,6-η)-cycloocta-1,5-diene]rhodium(I) (3) Refinement

**Table:**

Refinement on *F*^2^	76 restraints
Least-squares matrix: full	Hydrogen site location: inferred from neighbouring sites
*R*[*F*^2^ > 2σ(*F*^2^)] = 0.031	H-atom parameters constrained
*wR*(*F*^2^) = 0.081	*w* = 1/[σ^2^(*F*_o_^2^) + (0.0358*P*)^2^ + 1.9752*P*] where *P* = (*F*_o_^2^ + 2*F*_c_^2^)/3
*S* = 1.04	(Δ/σ)_max_ = 0.001
4565 reflections	Δρ_max_ = 1.52 e Å^−^^3^
255 parameters	Δρ_min_ = −0.81 e Å^−^^3^

### (4-Benzyl-1-ethyl-1,2,4-triazol-5-ylidene)chlorido[(1,2,5,6-η)-cycloocta-1,5-diene]rhodium(I) (3) Special details

**Table:**

Geometry. All esds (except the esd in the dihedral angle between two l.s. planes) are estimated using the full covariance matrix. The cell esds are taken into account individually in the estimation of esds in distances, angles and torsion angles; correlations between esds in cell parameters are only used when they are defined by crystal symmetry. An approximate (isotropic) treatment of cell esds is used for estimating esds involving l.s. planes.

### (4-Benzyl-1-ethyl-1,2,4-triazol-5-ylidene)chlorido[(1,2,5,6-η)-cycloocta-1,5-diene]rhodium(I) (3) Fractional atomic coordinates and isotropic or equivalent isotropic displacement parameters (Å^2^)

**Table:**

	*x*	*y*	*z*	*U*_iso_*/*U*_eq_	Occ. (<1)
Rh1	0.27552 (2)	0.66593 (2)	0.29266 (2)	0.01327 (7)	
Cl1	0.25333 (7)	0.40413 (6)	0.13661 (7)	0.02028 (14)	
N1	0.5919 (2)	0.7218 (3)	0.3706 (3)	0.0171 (4)	
N2	0.7014 (2)	0.7409 (3)	0.3184 (3)	0.0211 (5)	
N3	0.4997 (2)	0.7033 (2)	0.1553 (2)	0.0163 (4)	
C1	0.4670 (3)	0.6983 (3)	0.2743 (3)	0.0149 (5)	
C2	0.6410 (3)	0.7304 (3)	0.1877 (3)	0.0204 (5)	
H2	0.688723	0.740200	0.122556	0.024*	
C3	0.6207 (3)	0.6978 (4)	0.5025 (3)	0.0250 (6)	
H3A	0.537674	0.701727	0.537995	0.030*	
H3B	0.697305	0.777285	0.586192	0.030*	
C4	0.6603 (4)	0.5475 (5)	0.4604 (5)	0.0444 (10)	
H4A	0.583654	0.469047	0.378617	0.067*	
H4B	0.679873	0.532091	0.549390	0.067*	
H4C	0.742862	0.544324	0.426036	0.067*	
C5	0.4011 (3)	0.6655 (3)	0.0089 (3)	0.0189 (5)	
H5A	0.330815	0.577181	−0.021708	0.023*	
H5B	0.451310	0.637606	−0.068294	0.023*	
C6	0.3275 (3)	0.7874 (3)	0.0073 (3)	0.0164 (5)	
C7	0.1891 (3)	0.7497 (3)	−0.0781 (3)	0.0219 (5)	
H7	0.141512	0.649021	−0.129910	0.026*	
C8	0.1199 (3)	0.8584 (4)	−0.0881 (3)	0.0263 (6)	
H8	0.025642	0.831520	−0.147425	0.032*	
C9	0.1876 (3)	1.0051 (3)	−0.0123 (3)	0.0262 (6)	
H9	0.140024	1.079229	−0.018807	0.031*	
C10	0.3256 (3)	1.0441 (3)	0.0737 (3)	0.0228 (6)	
H10	0.372497	1.145028	0.126042	0.027*	
C11	0.3955 (3)	0.9354 (3)	0.0833 (3)	0.0187 (5)	
H11	0.490020	0.962496	0.142054	0.022*	
C12	0.2713 (3)	0.8941 (3)	0.3739 (3)	0.0174 (5)	
H12	0.341722	0.951878	0.355949	0.021*	0.632 (9)
H12A	0.350314	0.944188	0.359239	0.021*	0.368 (9)
C13	0.3193 (3)	0.8668 (3)	0.4972 (3)	0.0205 (5)	
H13	0.421300	0.905069	0.546067	0.025*	0.632 (9)
H13A	0.418707	0.908997	0.558770	0.025*	0.368 (9)
C14	0.2509 (6)	0.8500 (6)	0.6014 (6)	0.0197 (10)	0.632 (9)
H14A	0.247315	0.949556	0.680132	0.024*	0.632 (9)
H14B	0.306440	0.802184	0.653096	0.024*	0.632 (9)
C14*	0.2072 (11)	0.8742 (9)	0.5888 (11)	0.0201 (14)	0.368 (9)
H14C	0.256923	0.910057	0.697132	0.024*	0.368 (9)
H14D	0.149372	0.946201	0.583349	0.024*	0.368 (9)
C15	0.1021 (7)	0.7556 (7)	0.5175 (9)	0.0208 (12)	0.632 (9)
H15A	0.073741	0.715589	0.580074	0.025*	0.632 (9)
H15B	0.037709	0.820011	0.505153	0.025*	0.632 (9)
C15*	0.1130 (13)	0.7216 (10)	0.5238 (17)	0.0196 (15)	0.368 (9)
H15C	0.154703	0.670123	0.579203	0.024*	0.368 (9)
H15D	0.022140	0.736906	0.541601	0.024*	0.368 (9)
C16	0.0908 (3)	0.6259 (3)	0.3604 (3)	0.0185 (5)	
H16A	0.064600	0.518789	0.331145	0.022*	0.368 (9)
H16	0.061778	0.523484	0.343927	0.022*	0.632 (9)
C17	0.0495 (3)	0.6474 (4)	0.2361 (3)	0.0251 (6)	
H17A	−0.008579	0.560589	0.138352	0.030*	0.368 (9)
H17	0.000262	0.552259	0.141993	0.030*	0.632 (9)
C18	0.0166 (5)	0.7740 (6)	0.2205 (7)	0.0217 (10)	0.632 (9)
H18A	−0.076189	0.786817	0.233287	0.026*	0.632 (9)
H18B	0.012217	0.754635	0.116418	0.026*	0.632 (9)
C18*	0.0107 (8)	0.8150 (11)	0.2781 (13)	0.0211 (13)	0.368 (9)
H18C	−0.065240	0.806315	0.192892	0.025*	0.368 (9)
H18D	−0.021354	0.852070	0.369642	0.025*	0.368 (9)
C19	0.1217 (7)	0.9192 (8)	0.3355 (8)	0.0220 (11)	0.632 (9)
H19A	0.094780	0.965922	0.430144	0.026*	0.632 (9)
H19B	0.118846	0.988764	0.293483	0.026*	0.632 (9)
C19*	0.1386 (12)	0.9258 (15)	0.3074 (15)	0.0213 (15)	0.368 (9)
H19C	0.139711	0.922242	0.210271	0.026*	0.368 (9)
H19D	0.130999	1.027518	0.377395	0.026*	0.368 (9)

### (4-Benzyl-1-ethyl-1,2,4-triazol-5-ylidene)chlorido[(1,2,5,6-η)-cycloocta-1,5-diene]rhodium(I) (3) Atomic displacement parameters (Å^2^)

**Table:**

	*U*^11^	*U*^22^	*U*^33^	*U*^12^	*U*^13^	*U*^23^
Rh1	0.01295 (10)	0.01225 (10)	0.01177 (10)	0.00009 (7)	0.00308 (7)	0.00407 (8)
Cl1	0.0327 (3)	0.0064 (2)	0.0182 (3)	0.0014 (2)	0.0158 (3)	−0.0005 (2)
N1	0.0146 (10)	0.0192 (11)	0.0184 (11)	0.0047 (8)	0.0052 (8)	0.0094 (9)
N2	0.0160 (10)	0.0222 (11)	0.0270 (12)	0.0051 (9)	0.0097 (9)	0.0117 (10)
N3	0.0182 (10)	0.0159 (10)	0.0166 (10)	0.0054 (8)	0.0076 (8)	0.0079 (9)
C1	0.0172 (11)	0.0117 (11)	0.0158 (11)	0.0042 (9)	0.0057 (9)	0.0060 (9)
C2	0.0197 (12)	0.0182 (12)	0.0255 (14)	0.0054 (10)	0.0110 (11)	0.0101 (11)
C3	0.0225 (13)	0.0359 (16)	0.0181 (13)	0.0074 (12)	0.0028 (11)	0.0155 (12)
C4	0.058 (2)	0.061 (2)	0.049 (2)	0.040 (2)	0.0296 (19)	0.045 (2)
C5	0.0259 (13)	0.0164 (12)	0.0140 (12)	0.0054 (10)	0.0060 (10)	0.0068 (10)
C6	0.0204 (12)	0.0182 (12)	0.0134 (11)	0.0046 (10)	0.0078 (10)	0.0086 (10)
C7	0.0212 (13)	0.0223 (13)	0.0192 (13)	0.0003 (10)	0.0053 (10)	0.0082 (11)
C8	0.0192 (13)	0.0371 (17)	0.0233 (14)	0.0090 (12)	0.0055 (11)	0.0152 (13)
C9	0.0321 (15)	0.0292 (15)	0.0274 (15)	0.0177 (12)	0.0141 (12)	0.0179 (13)
C10	0.0314 (15)	0.0183 (13)	0.0234 (14)	0.0078 (11)	0.0119 (12)	0.0118 (11)
C11	0.0204 (12)	0.0181 (12)	0.0170 (12)	0.0020 (10)	0.0059 (10)	0.0081 (10)
C12	0.0163 (12)	0.0130 (11)	0.0184 (12)	0.0026 (9)	0.0062 (10)	0.0035 (10)
C13	0.0213 (13)	0.0144 (12)	0.0153 (12)	−0.0025 (10)	0.0034 (10)	0.0003 (10)
C14	0.0183 (19)	0.0187 (18)	0.0183 (15)	0.0044 (16)	0.0076 (16)	0.0048 (14)
C14*	0.018 (2)	0.020 (2)	0.0180 (18)	0.003 (2)	0.010 (2)	0.0041 (19)
C15	0.0186 (17)	0.021 (2)	0.0196 (16)	0.0030 (18)	0.0090 (14)	0.0054 (18)
C15*	0.018 (2)	0.019 (2)	0.0195 (19)	0.003 (2)	0.0093 (17)	0.005 (2)
C16	0.0132 (11)	0.0210 (13)	0.0190 (12)	−0.0020 (10)	0.0054 (10)	0.0082 (11)
C17	0.0108 (11)	0.0365 (16)	0.0243 (14)	−0.0079 (11)	−0.0009 (10)	0.0163 (13)
C18	0.0141 (14)	0.0277 (19)	0.026 (2)	0.0091 (14)	0.0091 (16)	0.0131 (16)
C18*	0.0150 (18)	0.025 (2)	0.027 (3)	0.0103 (18)	0.009 (2)	0.0127 (19)
C19	0.0169 (17)	0.0252 (17)	0.028 (3)	0.0101 (14)	0.0104 (16)	0.0130 (17)
C19*	0.016 (2)	0.025 (2)	0.026 (3)	0.0094 (18)	0.011 (2)	0.012 (2)

### (4-Benzyl-1-ethyl-1,2,4-triazol-5-ylidene)chlorido[(1,2,5,6-η)-cycloocta-1,5-diene]rhodium(I) (3) Geometric parameters (Å, º)

**Table:**

N1—N2	1.382 (3)	C12—C13	1.411 (4)
N1—C1	1.343 (3)	C12—C19	1.546 (8)
N1—C3	1.459 (4)	C12—C19*	1.491 (15)
N2—C2	1.298 (4)	C13—H13	1.0000
N3—C1	1.367 (3)	C13—H13A	1.0000
N3—C2	1.371 (3)	C13—C14	1.473 (6)
N3—C5	1.465 (3)	C13—C14*	1.627 (10)
Rh1—Cl1	2.3960 (6)	C14—H14A	0.9900
Rh1—C1	2.014 (3)	C14—H14B	0.9900
Rh1—C12	2.116 (3)	C14—C15	1.549 (6)
Rh1—C13	2.105 (3)	C14*—H14C	0.9900
Rh1—C16	2.220 (2)	C14*—H14D	0.9900
Rh1—C17	2.201 (3)	C14*—C15*	1.539 (9)
C2—H2	0.9500	C15—H15A	0.9900
C3—H3A	0.9900	C15—H15B	0.9900
C3—H3B	0.9900	C15—C16	1.539 (8)
C3—C4	1.519 (5)	C15*—H15C	0.9900
C4—H4A	0.9800	C15*—H15D	0.9900
C4—H4B	0.9800	C15*—C16	1.471 (15)
C4—H4C	0.9800	C16—H16A	1.0000
C5—H5A	0.9900	C16—H16	1.0000
C5—H5B	0.9900	C16—C17	1.376 (4)
C5—C6	1.509 (4)	C17—H17A	1.0000
C6—C7	1.391 (4)	C17—H17	1.0000
C6—C11	1.391 (4)	C17—C18	1.435 (6)
C7—H7	0.9500	C17—C18*	1.678 (10)
C7—C8	1.392 (4)	C18—H18A	0.9900
C8—H8	0.9500	C18—H18B	0.9900
C8—C9	1.380 (4)	C18—C19	1.539 (6)
C9—H9	0.9500	C18*—H18C	0.9900
C9—C10	1.389 (4)	C18*—H18D	0.9900
C10—H10	0.9500	C18*—C19*	1.540 (8)
C10—C11	1.394 (4)	C19—H19A	0.9900
C11—H11	0.9500	C19—H19B	0.9900
C12—H12	1.0000	C19*—H19C	0.9900
C12—H12A	1.0000	C19*—H19D	0.9900
			
N1—C1—N3	102.7 (2)	C12—C13—H13	111.5
C1—Rh1—Cl1	89.14 (7)	C12—C13—H13A	117.2
C1—Rh1—C12	91.12 (10)	C12—C13—C14	132.2 (3)
C1—Rh1—C13	92.94 (10)	C12—C13—C14*	113.5 (5)
C1—Rh1—C16	166.45 (10)	C14—C13—Rh1	110.9 (2)
C1—Rh1—C17	157.29 (11)	C14—C13—H13	111.5
C12—Rh1—Cl1	164.69 (8)	C14*—C13—Rh1	112.7 (3)
C12—Rh1—C16	92.53 (10)	C14*—C13—H13A	117.2
C12—Rh1—C17	81.74 (11)	C13—C14—H14A	109.2
C13—Rh1—Cl1	156.20 (8)	C13—C14—H14B	109.2
C13—Rh1—C12	39.05 (11)	C13—C14—C15	112.1 (6)
C13—Rh1—C16	81.79 (10)	H14A—C14—H14B	107.9
C13—Rh1—C17	94.87 (11)	C15—C14—H14A	109.2
C16—Rh1—Cl1	90.76 (7)	C15—C14—H14B	109.2
C17—Rh1—Cl1	92.22 (9)	C13—C14*—H14C	109.2
C17—Rh1—C16	36.25 (10)	C13—C14*—H14D	109.2
N2—N1—C3	117.8 (2)	H14C—C14*—H14D	107.9
C1—N1—N2	113.9 (2)	C15*—C14*—C13	112.0 (9)
C1—N1—C3	126.8 (2)	C15*—C14*—H14C	109.2
C2—N2—N1	103.2 (2)	C15*—C14*—H14D	109.2
C1—N3—C2	108.6 (2)	C14—C15—H15A	109.2
C1—N3—C5	125.8 (2)	C14—C15—H15B	109.2
C2—N3—C5	125.1 (2)	H15A—C15—H15B	107.9
N1—C1—Rh1	130.73 (19)	C16—C15—C14	112.2 (6)
N3—C1—Rh1	126.53 (19)	C16—C15—H15A	109.2
N2—C2—N3	111.5 (2)	C16—C15—H15B	109.2
N2—C2—H2	124.3	C14*—C15*—H15C	108.9
N3—C2—H2	124.3	C14*—C15*—H15D	108.9
N1—C3—H3A	109.7	H15C—C15*—H15D	107.7
N1—C3—H3B	109.7	C16—C15*—C14*	113.4 (11)
N1—C3—C4	109.8 (3)	C16—C15*—H15C	108.9
H3A—C3—H3B	108.2	C16—C15*—H15D	108.9
C4—C3—H3A	109.7	Rh1—C16—H16A	110.9
C4—C3—H3B	109.7	Rh1—C16—H16	116.7
C3—C4—H4A	109.5	C15—C16—Rh1	109.1 (3)
C3—C4—H4B	109.5	C15—C16—H16	116.7
C3—C4—H4C	109.5	C15*—C16—Rh1	111.4 (5)
H4A—C4—H4B	109.5	C15*—C16—H16A	110.9
H4A—C4—H4C	109.5	C17—C16—Rh1	71.12 (15)
H4B—C4—H4C	109.5	C17—C16—C15	118.1 (4)
N3—C5—H5A	108.5	C17—C16—C15*	133.2 (5)
N3—C5—H5B	108.5	C17—C16—H16A	110.9
N3—C5—C6	115.0 (2)	C17—C16—H16	116.7
H5A—C5—H5B	107.5	Rh1—C17—H17A	117.5
C6—C5—H5A	108.5	Rh1—C17—H17	111.5
C6—C5—H5B	108.5	C16—C17—Rh1	72.63 (15)
C7—C6—C5	118.9 (2)	C16—C17—H17A	117.5
C11—C6—C5	121.9 (2)	C16—C17—H17	111.5
C11—C6—C7	119.1 (3)	C16—C17—C18	132.5 (4)
C6—C7—H7	119.8	C16—C17—C18*	113.6 (4)
C6—C7—C8	120.4 (3)	C18—C17—Rh1	108.7 (2)
C8—C7—H7	119.8	C18—C17—H17	111.5
C7—C8—H8	119.9	C18*—C17—Rh1	109.9 (3)
C9—C8—C7	120.3 (3)	C18*—C17—H17A	117.5
C9—C8—H8	119.9	C17—C18—H18A	108.9
C8—C9—H9	120.1	C17—C18—H18B	108.9
C8—C9—C10	119.8 (3)	C17—C18—C19	113.5 (5)
C10—C9—H9	120.1	H18A—C18—H18B	107.7
C9—C10—H10	119.9	C19—C18—H18A	108.9
C9—C10—C11	120.1 (3)	C19—C18—H18B	108.9
C11—C10—H10	119.9	C17—C18*—H18C	109.6
C6—C11—C10	120.3 (3)	C17—C18*—H18D	109.6
C6—C11—H11	119.9	H18C—C18*—H18D	108.1
C10—C11—H11	119.9	C19*—C18*—C17	110.4 (8)
Rh1—C12—H12	116.0	C19*—C18*—H18C	109.6
Rh1—C12—H12A	110.1	C19*—C18*—H18D	109.6
C13—C12—Rh1	70.06 (15)	C12—C19—H19A	109.1
C13—C12—H12	116.0	C12—C19—H19B	109.1
C13—C12—H12A	110.1	C18—C19—C12	112.6 (6)
C13—C12—C19	119.0 (3)	C18—C19—H19A	109.1
C13—C12—C19*	133.8 (5)	C18—C19—H19B	109.1
C19—C12—Rh1	111.6 (3)	H19A—C19—H19B	107.8
C19—C12—H12	116.0	C12—C19*—C18*	112.8 (10)
C19*—C12—Rh1	114.9 (5)	C12—C19*—H19C	109.0
C19*—C12—H12A	110.1	C12—C19*—H19D	109.0
Rh1—C13—H13	111.5	C18*—C19*—H19C	109.0
Rh1—C13—H13A	117.2	C18*—C19*—H19D	109.0
C12—C13—Rh1	70.88 (15)	H19C—C19*—H19D	107.8
			
Rh1—C12—C13—C14	−101.2 (4)	C5—C6—C11—C10	177.1 (2)
Rh1—C12—C13—C14*	−107.1 (3)	C6—C7—C8—C9	−0.6 (4)
Rh1—C12—C19—C18	−12.2 (6)	C7—C6—C11—C10	−0.1 (4)
Rh1—C12—C19*—C18*	36.1 (11)	C7—C8—C9—C10	0.4 (4)
Rh1—C13—C14—C15	−40.9 (6)	C8—C9—C10—C11	0.0 (4)
Rh1—C13—C14*—C15*	12.1 (11)	C9—C10—C11—C6	−0.2 (4)
Rh1—C16—C17—C18	−99.7 (4)	C11—C6—C7—C8	0.4 (4)
Rh1—C16—C17—C18*	−104.7 (3)	C12—C13—C14—C15	41.8 (7)
Rh1—C17—C18—C19	−37.7 (6)	C12—C13—C14*—C15*	90.2 (10)
Rh1—C17—C18*—C19*	13.6 (10)	C13—C12—C19—C18	−90.6 (6)
N1—N2—C2—N3	−1.0 (3)	C13—C12—C19*—C18*	−49.5 (13)
N2—N1—C1—Rh1	−179.34 (18)	C13—C14—C15—C16	37.7 (8)
N2—N1—C1—N3	−0.2 (3)	C13—C14*—C15*—C16	−29.5 (14)
N2—N1—C3—C4	−68.3 (3)	C14—C15—C16—Rh1	−16.1 (7)
N3—C5—C6—C7	−142.6 (2)	C14—C15—C16—C17	−94.4 (6)
N3—C5—C6—C11	40.2 (3)	C14*—C15*—C16—Rh1	32.5 (12)
C1—N1—N2—C2	0.7 (3)	C14*—C15*—C16—C17	−51.3 (13)
C1—N1—C3—C4	97.3 (3)	C15—C16—C17—Rh1	102.1 (3)
C1—N3—C2—N2	1.0 (3)	C15—C16—C17—C18	2.4 (5)
C1—N3—C5—C6	81.8 (3)	C15*—C16—C17—Rh1	102.0 (7)
C2—N3—C1—Rh1	178.78 (18)	C15*—C16—C17—C18*	−2.6 (8)
C2—N3—C1—N1	−0.5 (3)	C16—C17—C18—C19	45.5 (7)
C2—N3—C5—C6	−107.3 (3)	C16—C17—C18*—C19*	92.7 (9)
C3—N1—N2—C2	168.1 (2)	C17—C18—C19—C12	34.1 (8)
C3—N1—C1—Rh1	14.6 (4)	C17—C18*—C19*—C12	−31.3 (13)
C3—N1—C1—N3	−166.2 (3)	C19—C12—C13—Rh1	104.3 (3)
C5—N3—C1—Rh1	−9.1 (4)	C19—C12—C13—C14	3.1 (6)
C5—N3—C1—N1	171.7 (2)	C19*—C12—C13—Rh1	105.9 (7)
C5—N3—C2—N2	−171.2 (2)	C19*—C12—C13—C14*	−1.2 (8)
C5—C6—C7—C8	−176.9 (2)		

### (4-Benzyl-1-ethyl-1,2,4-triazol-5-ylidene)chlorido[(1,2,5,6-η)-cycloocta-1,5-diene]rhodium(I) (3) Hydrogen-bond geometry (Å, º)

**Table:**

*D*—H···*A*	*D*—H	H···*A*	*D*···*A*	*D*—H···*A*
C2—H2···Cl1^i^	0.95	2.64	3.461 (3)	145

Symmetry code: (i) −*x*+1, −*y*+1, −*z*.

### (4-Benzyl-1-ethyl-1,2,4-triazol-5-ylidene)[(1,2,5,6-η)-cycloocta-1,5-diene](triphenylphosphane)iridium(I) tetrafluoridoborate (5) Crystal data

**Table:**

[Ir(C_8_H_12_)(C_11_H_13_N_3_)(C_18_H_15_P)]BF_4_	*Z* = 2
*M**_r_* = 836.70	*F*(000) = 832
Triclinic, *P*1	*D*_x_ = 1.679 Mg m^−^^3^
*a* = 9.47197 (15) Å	Mo *K*α radiation, λ = 0.71073 Å
*b* = 9.50712 (15) Å	Cell parameters from 35729 reflections
*c* = 18.7104 (3) Å	θ = 2.2–28.3°
α = 79.8203 (14)°	µ = 4.14 mm^−^^1^
β = 86.1222 (13)°	*T* = 100 K
γ = 89.3859 (13)°	Block, red
*V* = 1654.57 (5) Å^3^	0.31 × 0.27 × 0.23 mm

### (4-Benzyl-1-ethyl-1,2,4-triazol-5-ylidene)[(1,2,5,6-η)-cycloocta-1,5-diene](triphenylphosphane)iridium(I) tetrafluoridoborate (5) Data collection

**Table:**

Rigaku XtaLAB Synergy-S diffractometer	14450 reflections with *I* > 2σ(*I*)
Detector resolution: 10.0 pixels mm^-1^	*R*_int_ = 0.045
ω scans	θ_max_ = 28.3°, θ_min_ = 2.2°
Absorption correction: multi-scan (SCALE3 ABSPACK; Rigaku OD, 2024)	*h* = −12→12
*T*_min_ = 0.857, *T*_max_ = 1.000	*k* = −12→12
50908 measured reflections	*l* = −24→24
15249 independent reflections	

### (4-Benzyl-1-ethyl-1,2,4-triazol-5-ylidene)[(1,2,5,6-η)-cycloocta-1,5-diene](triphenylphosphane)iridium(I) tetrafluoridoborate (5) Refinement

**Table:**

Refinement on *F*^2^	Hydrogen site location: inferred from neighbouring sites
Least-squares matrix: full	H-atom parameters constrained
*R*[*F*^2^ > 2σ(*F*^2^)] = 0.027	*w* = 1/[σ^2^(*F*_o_^2^) + (0.0302*P*)^2^ + 0.5824*P*] where *P* = (*F*_o_^2^ + 2*F*_c_^2^)/3
*wR*(*F*^2^) = 0.062	(Δ/σ)_max_ = 0.001
*S* = 1.05	Δρ_max_ = 3.38 e Å^−^^3^
15249 reflections	Δρ_min_ = −1.12 e Å^−^^3^
849 parameters	Absolute structure: Flack *x* determined using 6303 quotients [(*I*^+^)-(*I*^-^)]/[(*I*^+^)+(*I*^-^)] (Parsons *et al.*, 2013)
3 restraints	Absolute structure parameter: −0.008 (3)

### (4-Benzyl-1-ethyl-1,2,4-triazol-5-ylidene)[(1,2,5,6-η)-cycloocta-1,5-diene](triphenylphosphane)iridium(I) tetrafluoridoborate (5) Special details

**Table:**

Geometry. All esds (except the esd in the dihedral angle between two l.s. planes) are estimated using the full covariance matrix. The cell esds are taken into account individually in the estimation of esds in distances, angles and torsion angles; correlations between esds in cell parameters are only used when they are defined by crystal symmetry. An approximate (isotropic) treatment of cell esds is used for estimating esds involving l.s. planes.

### (4-Benzyl-1-ethyl-1,2,4-triazol-5-ylidene)[(1,2,5,6-η)-cycloocta-1,5-diene](triphenylphosphane)iridium(I) tetrafluoridoborate (5) Fractional atomic coordinates and isotropic or equivalent isotropic displacement parameters (Å^2^)

**Table:**

	*x*	*y*	*z*	*U*_iso_*/*U*_eq_	
Ir1	1.06227 (2)	0.49154 (2)	0.52321 (2)	0.01293 (7)	
P1	0.82029 (17)	0.52037 (17)	0.54790 (8)	0.0123 (3)	
N1	1.0682 (5)	0.1685 (5)	0.5614 (3)	0.0148 (10)	
N2	1.0802 (6)	0.0502 (6)	0.6159 (3)	0.0200 (11)	
N3	1.0778 (5)	0.2541 (5)	0.6579 (3)	0.0151 (10)	
C1	1.0645 (6)	0.2927 (6)	0.5855 (3)	0.0131 (11)	
C2	1.0853 (6)	0.1068 (7)	0.6741 (3)	0.0185 (12)	
H2	1.093215	0.053613	0.721706	0.022*	
C3	1.0589 (7)	0.1455 (7)	0.4868 (3)	0.0197 (13)	
H3A	1.137162	0.082368	0.474358	0.024*	
H3B	1.069566	0.238075	0.453121	0.024*	
C4	0.9186 (7)	0.0781 (7)	0.4770 (4)	0.0240 (14)	
H4A	0.921859	0.048676	0.429345	0.036*	
H4B	0.842463	0.147830	0.480110	0.036*	
H4C	0.900957	−0.005572	0.515361	0.036*	
C5	1.0712 (7)	0.3508 (7)	0.7115 (3)	0.0202 (13)	
H5A	0.980042	0.336045	0.740945	0.024*	
H5B	1.073661	0.450853	0.685236	0.024*	
C6	1.1909 (7)	0.3285 (6)	0.7619 (3)	0.0173 (12)	
C7	1.3315 (7)	0.3085 (7)	0.7379 (4)	0.0213 (13)	
H7	1.353850	0.301092	0.688397	0.026*	
C8	1.4385 (9)	0.2995 (10)	0.7852 (5)	0.0241 (19)	
H8	1.533525	0.285973	0.768007	0.029*	
C9	1.4079 (7)	0.3100 (8)	0.8576 (4)	0.0277 (15)	
H9	1.481452	0.304428	0.890112	0.033*	
C10	1.2684 (8)	0.3286 (9)	0.8821 (4)	0.0303 (16)	
H10	1.246672	0.335398	0.931684	0.036*	
C11	1.1596 (8)	0.3375 (8)	0.8345 (4)	0.0236 (15)	
H11	1.064424	0.349771	0.851864	0.028*	
C12	0.7122 (6)	0.3569 (6)	0.5659 (3)	0.0136 (11)	
C13	0.6161 (6)	0.3216 (7)	0.5193 (3)	0.0176 (12)	
H13	0.598096	0.387348	0.476456	0.021*	
C14	0.5455 (6)	0.1911 (7)	0.5346 (4)	0.0205 (13)	
H14	0.479066	0.168770	0.502435	0.025*	
C15	0.5718 (7)	0.0936 (7)	0.5967 (4)	0.0217 (13)	
H15	0.526135	0.003126	0.606045	0.026*	
C16	0.6649 (7)	0.1284 (7)	0.6450 (3)	0.0199 (12)	
H16	0.680775	0.063197	0.688376	0.024*	
C17	0.7349 (6)	0.2592 (7)	0.6296 (3)	0.0173 (12)	
H17	0.798851	0.282698	0.662632	0.021*	
C18	0.7342 (6)	0.6427 (6)	0.4775 (3)	0.0137 (11)	
C19	0.7264 (6)	0.6045 (7)	0.4088 (3)	0.0187 (12)	
H19	0.764770	0.515951	0.399976	0.022*	
C20	0.6634 (6)	0.6944 (7)	0.3542 (3)	0.0191 (12)	
H20	0.655143	0.665661	0.308508	0.023*	
C21	0.6117 (6)	0.8270 (7)	0.3652 (4)	0.0240 (14)	
H21	0.568635	0.888692	0.327169	0.029*	
C22	0.6235 (6)	0.8686 (7)	0.4318 (4)	0.0209 (13)	
H22	0.590858	0.960253	0.438959	0.025*	
C23	0.6826 (6)	0.7773 (7)	0.4882 (3)	0.0183 (12)	
H23	0.688213	0.805738	0.534100	0.022*	
C24	0.7786 (6)	0.5997 (6)	0.6288 (3)	0.0143 (11)	
C25	0.8732 (7)	0.6968 (7)	0.6488 (3)	0.0185 (12)	
H25	0.964033	0.711639	0.623850	0.022*	
C26	0.8352 (7)	0.7706 (7)	0.7044 (3)	0.0202 (13)	
H26	0.898471	0.838174	0.716560	0.024*	
C27	0.7046 (7)	0.7461 (7)	0.7423 (3)	0.0226 (13)	
H27	0.678303	0.797595	0.780251	0.027*	
C28	0.6112 (7)	0.6464 (7)	0.7253 (4)	0.0243 (14)	
H28	0.523277	0.626991	0.752643	0.029*	
C29	0.6484 (6)	0.5753 (7)	0.6675 (3)	0.0174 (12)	
H29	0.583957	0.509648	0.654621	0.021*	
C30	1.2744 (8)	0.4394 (8)	0.4772 (5)	0.0220 (18)	
H30	1.299439	0.335927	0.489618	0.026*	
C31	1.2894 (6)	0.5147 (7)	0.5337 (4)	0.0255 (15)	
H31	1.324965	0.455527	0.578512	0.031*	
C32	1.3368 (7)	0.6695 (8)	0.5206 (4)	0.0314 (16)	
H32A	1.393426	0.684670	0.561144	0.038*	
H32B	1.399176	0.688202	0.475299	0.038*	
C33	1.2170 (7)	0.7765 (7)	0.5141 (4)	0.0266 (15)	
H33A	1.250461	0.865495	0.481870	0.032*	
H33B	1.190865	0.800035	0.562772	0.032*	
C34	1.0860 (7)	0.7236 (7)	0.4843 (4)	0.0239 (14)	
H34	0.998520	0.779965	0.492535	0.029*	
C35	1.0857 (7)	0.6576 (7)	0.4240 (4)	0.0211 (13)	
H35	0.997800	0.674724	0.396984	0.025*	
C36	1.2186 (7)	0.6403 (8)	0.3757 (4)	0.0280 (15)	
H36A	1.193020	0.650640	0.324556	0.034*	
H36B	1.285983	0.717749	0.378446	0.034*	
C37	1.2903 (8)	0.4984 (8)	0.3967 (4)	0.0307 (16)	
H37A	1.392272	0.509165	0.381450	0.037*	
H37B	1.250388	0.428787	0.369945	0.037*	
Ir1'	0.02978 (2)	0.84457 (2)	0.12244 (2)	0.01259 (7)	
P1'	0.01122 (15)	0.60664 (16)	0.11049 (9)	0.0126 (3)	
N1'	0.3434 (5)	0.8664 (6)	0.0955 (3)	0.0176 (11)	
N2'	0.4595 (5)	0.9040 (6)	0.0474 (3)	0.0241 (12)	
N3'	0.2588 (5)	0.9244 (6)	−0.0078 (3)	0.0176 (11)	
C1'	0.2193 (7)	0.8757 (7)	0.0646 (3)	0.0181 (12)	
C2'	0.4031 (7)	0.9408 (7)	−0.0143 (4)	0.0227 (14)	
H2'	0.455658	0.975271	−0.058944	0.027*	
C3'	0.3643 (7)	0.8194 (8)	0.1730 (4)	0.0252 (14)	
H3'A	0.270935	0.810124	0.200583	0.030*	
H3'B	0.418892	0.893255	0.190583	0.030*	
C4'	0.4411 (8)	0.6793 (8)	0.1881 (4)	0.0290 (15)	
H4'A	0.384655	0.604396	0.173638	0.044*	
H4'B	0.455714	0.654774	0.240132	0.044*	
H4'C	0.532918	0.687197	0.160188	0.044*	
C5'	0.1583 (7)	0.9568 (8)	−0.0664 (3)	0.0234 (14)	
H5'A	0.156755	1.061372	−0.083625	0.028*	
H5'B	0.062005	0.927213	−0.045648	0.028*	
C6'	0.1942 (7)	0.8839 (7)	−0.1305 (3)	0.0192 (12)	
C7'	0.3062 (7)	0.9334 (8)	−0.1798 (4)	0.0284 (15)	
H7'	0.363049	1.010488	−0.172178	0.034*	
C8'	0.3347 (8)	0.8695 (10)	−0.2404 (4)	0.0379 (19)	
H8'	0.411496	0.902811	−0.274284	0.045*	
C9'	0.2520 (9)	0.7577 (10)	−0.2518 (5)	0.041 (2)	
H9'	0.272101	0.714016	−0.293314	0.049*	
C10'	0.1409 (8)	0.7099 (9)	−0.2031 (4)	0.0357 (18)	
H10'	0.083751	0.633266	−0.210954	0.043*	
C11'	0.1115 (7)	0.7732 (8)	−0.1424 (4)	0.0264 (14)	
H11'	0.034042	0.740070	−0.108909	0.032*	
C12'	−0.0890 (6)	0.5903 (6)	0.0324 (3)	0.0142 (11)	
C13'	−0.1776 (6)	0.6992 (7)	0.0024 (3)	0.0171 (12)	
H13'	−0.182564	0.786125	0.020848	0.021*	
C14'	−0.2591 (7)	0.6816 (7)	−0.0547 (3)	0.0229 (14)	
H14'	−0.319133	0.756430	−0.075362	0.028*	
C15'	−0.2520 (7)	0.5535 (8)	−0.0811 (3)	0.0221 (14)	
H15'	−0.306577	0.541596	−0.120333	0.026*	
C16'	−0.1666 (6)	0.4439 (7)	−0.0508 (3)	0.0208 (13)	
H16'	−0.164880	0.355723	−0.068064	0.025*	
C17'	−0.0828 (6)	0.4623 (7)	0.0051 (3)	0.0161 (12)	
H17'	−0.021349	0.387948	0.024705	0.019*	
C18'	−0.0861 (6)	0.4891 (6)	0.1852 (3)	0.0146 (11)	
C19'	−0.2243 (6)	0.4439 (7)	0.1788 (3)	0.0175 (12)	
H19'	−0.266815	0.471279	0.133886	0.021*	
C20'	−0.3003 (7)	0.3602 (7)	0.2366 (3)	0.0206 (13)	
H20'	−0.393801	0.331003	0.230721	0.025*	
C21'	−0.2407 (8)	0.3187 (8)	0.3029 (4)	0.0245 (16)	
H21'	−0.292301	0.261387	0.342693	0.029*	
C22'	−0.1008 (7)	0.3643 (7)	0.3098 (4)	0.0221 (13)	
H22'	−0.057185	0.334991	0.354219	0.026*	
C23'	−0.0277 (7)	0.4504 (7)	0.2529 (3)	0.0203 (13)	
H23'	0.063700	0.484213	0.259408	0.024*	
C24'	0.1752 (6)	0.5112 (6)	0.0924 (3)	0.0154 (12)	
C25'	0.2574 (6)	0.5680 (7)	0.0280 (3)	0.0185 (12)	
H25'	0.222770	0.647059	−0.004511	0.022*	
C26'	0.3881 (7)	0.5096 (7)	0.0118 (4)	0.0231 (14)	
H26'	0.443617	0.550626	−0.030789	0.028*	
C27'	0.4383 (7)	0.3905 (8)	0.0580 (4)	0.0245 (14)	
H27'	0.528142	0.350912	0.047275	0.029*	
C28'	0.3558 (7)	0.3308 (7)	0.1197 (4)	0.0221 (13)	
H28'	0.388319	0.248358	0.150609	0.027*	
C29'	0.2254 (6)	0.3904 (7)	0.1369 (3)	0.0183 (12)	
H29'	0.170206	0.348135	0.179371	0.022*	
C30'	−0.0218 (7)	1.0714 (7)	0.0894 (4)	0.0211 (13)	
H30'	0.028961	1.117903	0.042786	0.025*	
C31'	0.0578 (7)	1.0618 (6)	0.1499 (4)	0.0197 (13)	
H31'	0.155892	1.101009	0.138636	0.024*	
C32'	−0.0029 (7)	1.0715 (7)	0.2251 (4)	0.0225 (13)	
H32C	−0.089674	1.130620	0.221337	0.027*	
H32D	0.066128	1.119975	0.249943	0.027*	
C33'	−0.0393 (7)	0.9251 (7)	0.2712 (3)	0.0219 (13)	
H33C	0.045621	0.886355	0.295979	0.026*	
H33D	−0.113904	0.937363	0.309316	0.026*	
C34'	−0.0901 (6)	0.8174 (7)	0.2273 (3)	0.0173 (12)	
H34'	−0.093226	0.716701	0.254271	0.021*	
C35'	−0.1867 (8)	0.8462 (8)	0.1730 (4)	0.0178 (16)	
H35'	−0.244981	0.761717	0.168051	0.021*	
C36'	−0.2619 (6)	0.9891 (7)	0.1534 (4)	0.0227 (14)	
H36C	−0.272006	1.035565	0.196866	0.027*	
H36D	−0.357987	0.972107	0.138944	0.027*	
C37'	−0.1811 (7)	1.0895 (7)	0.0912 (4)	0.0229 (13)	
H37C	−0.214320	1.072644	0.044343	0.027*	
H37D	−0.204334	1.189464	0.095616	0.027*	
F1	0.6525 (6)	0.0303 (7)	0.8562 (3)	0.0384 (15)	
F2	0.7677 (5)	0.2422 (5)	0.8229 (3)	0.0377 (10)	
F3	0.8675 (5)	0.0396 (6)	0.7953 (3)	0.0449 (13)	
F4	0.8493 (6)	0.0753 (6)	0.9118 (3)	0.0507 (13)	
B1	0.7835 (7)	0.0963 (8)	0.8479 (4)	0.0196 (14)	
F1'	0.2691 (7)	0.0025 (6)	0.3537 (4)	0.0625 (18)	
F2'	0.2486 (5)	0.2355 (5)	0.3050 (2)	0.0384 (11)	
F3'	0.4319 (6)	0.1021 (6)	0.2688 (3)	0.0507 (13)	
F4'	0.4216 (7)	0.1615 (7)	0.3801 (3)	0.076 (2)	
B1'	0.3431 (8)	0.1248 (9)	0.3276 (4)	0.0247 (16)	

### (4-Benzyl-1-ethyl-1,2,4-triazol-5-ylidene)[(1,2,5,6-η)-cycloocta-1,5-diene](triphenylphosphane)iridium(I) tetrafluoridoborate (5) Atomic displacement parameters (Å^2^)

**Table:**

	*U*^11^	*U*^22^	*U*^33^	*U*^12^	*U*^13^	*U*^23^
Ir1	0.00866 (13)	0.01089 (15)	0.01797 (15)	0.00256 (10)	−0.00083 (10)	0.00079 (11)
P1	0.0109 (7)	0.0130 (7)	0.0130 (7)	0.0034 (6)	−0.0011 (6)	−0.0024 (6)
N1	0.015 (2)	0.014 (3)	0.015 (2)	0.001 (2)	−0.0027 (18)	0.0006 (19)
N2	0.023 (3)	0.014 (3)	0.020 (3)	0.003 (2)	−0.004 (2)	0.006 (2)
N3	0.013 (2)	0.015 (2)	0.015 (2)	−0.0007 (19)	−0.0018 (18)	0.0006 (19)
C1	0.008 (3)	0.013 (3)	0.018 (3)	0.004 (2)	−0.003 (2)	−0.003 (2)
C2	0.018 (3)	0.016 (3)	0.019 (3)	0.002 (2)	0.000 (2)	0.001 (2)
C3	0.026 (3)	0.015 (3)	0.018 (3)	0.003 (2)	−0.001 (2)	−0.001 (2)
C4	0.026 (3)	0.020 (3)	0.028 (3)	0.002 (3)	−0.005 (3)	−0.008 (3)
C5	0.022 (3)	0.021 (3)	0.018 (3)	0.004 (2)	0.001 (2)	−0.006 (2)
C6	0.021 (3)	0.008 (3)	0.023 (3)	0.004 (2)	−0.004 (2)	−0.002 (2)
C7	0.021 (3)	0.022 (3)	0.021 (3)	0.002 (3)	−0.002 (2)	−0.004 (3)
C8	0.017 (4)	0.021 (5)	0.031 (5)	−0.003 (3)	−0.001 (3)	0.004 (4)
C9	0.029 (4)	0.026 (4)	0.029 (4)	−0.004 (3)	−0.011 (3)	−0.003 (3)
C10	0.036 (4)	0.040 (4)	0.018 (3)	−0.002 (3)	−0.008 (3)	−0.009 (3)
C11	0.021 (4)	0.032 (4)	0.020 (3)	0.000 (3)	−0.002 (3)	−0.008 (3)
C12	0.010 (3)	0.012 (3)	0.018 (3)	0.005 (2)	0.000 (2)	−0.003 (2)
C13	0.018 (3)	0.016 (3)	0.018 (3)	0.006 (2)	−0.002 (2)	−0.003 (2)
C14	0.015 (3)	0.021 (3)	0.027 (3)	0.002 (2)	−0.006 (2)	−0.007 (3)
C15	0.022 (3)	0.015 (3)	0.028 (3)	−0.002 (2)	0.000 (3)	−0.004 (3)
C16	0.022 (3)	0.015 (3)	0.021 (3)	0.002 (2)	−0.001 (2)	−0.001 (2)
C17	0.016 (3)	0.019 (3)	0.019 (3)	0.002 (2)	−0.003 (2)	−0.006 (2)
C18	0.010 (3)	0.016 (3)	0.015 (3)	0.004 (2)	−0.002 (2)	0.000 (2)
C19	0.016 (3)	0.019 (3)	0.021 (3)	0.002 (2)	0.001 (2)	−0.004 (2)
C20	0.014 (3)	0.027 (3)	0.014 (3)	−0.004 (2)	−0.001 (2)	0.001 (2)
C21	0.016 (3)	0.028 (4)	0.023 (3)	−0.001 (3)	−0.004 (2)	0.010 (3)
C22	0.015 (3)	0.018 (3)	0.028 (3)	0.002 (2)	0.000 (2)	0.002 (3)
C23	0.015 (3)	0.019 (3)	0.021 (3)	0.001 (2)	−0.002 (2)	−0.003 (2)
C24	0.018 (3)	0.009 (3)	0.015 (3)	0.005 (2)	−0.002 (2)	−0.001 (2)
C25	0.018 (3)	0.019 (3)	0.018 (3)	0.000 (2)	−0.002 (2)	−0.002 (3)
C26	0.025 (3)	0.017 (3)	0.021 (3)	0.002 (3)	−0.008 (2)	−0.005 (2)
C27	0.034 (4)	0.018 (3)	0.016 (3)	0.006 (3)	0.000 (3)	−0.003 (2)
C28	0.029 (3)	0.022 (3)	0.021 (3)	0.004 (3)	0.007 (3)	−0.005 (3)
C29	0.017 (3)	0.020 (3)	0.015 (3)	0.002 (2)	0.000 (2)	−0.005 (2)
C30	0.011 (3)	0.015 (4)	0.037 (4)	0.007 (3)	0.000 (3)	0.002 (3)
C31	0.009 (3)	0.020 (3)	0.044 (4)	0.000 (2)	−0.005 (3)	0.006 (3)
C32	0.022 (3)	0.026 (4)	0.046 (4)	−0.006 (3)	−0.007 (3)	−0.004 (3)
C33	0.026 (3)	0.013 (3)	0.039 (4)	−0.006 (3)	0.006 (3)	0.000 (3)
C34	0.024 (3)	0.017 (3)	0.027 (4)	0.005 (3)	0.005 (3)	0.006 (3)
C35	0.013 (3)	0.024 (3)	0.022 (3)	0.006 (2)	−0.002 (2)	0.007 (3)
C36	0.018 (3)	0.040 (4)	0.025 (3)	0.005 (3)	0.002 (3)	−0.004 (3)
C37	0.024 (4)	0.028 (4)	0.036 (4)	0.005 (3)	0.006 (3)	0.004 (3)
Ir1'	0.01161 (13)	0.01094 (15)	0.01562 (14)	−0.00217 (11)	0.00095 (10)	−0.00402 (11)
P1'	0.0108 (7)	0.0112 (7)	0.0159 (8)	−0.0010 (5)	−0.0011 (5)	−0.0027 (6)
N1'	0.011 (2)	0.025 (3)	0.017 (3)	−0.008 (2)	0.0012 (19)	−0.005 (2)
N2'	0.015 (3)	0.030 (3)	0.025 (3)	−0.008 (2)	0.006 (2)	−0.003 (2)
N3'	0.017 (2)	0.020 (3)	0.014 (2)	−0.001 (2)	0.0010 (19)	0.000 (2)
C1'	0.019 (3)	0.015 (3)	0.021 (3)	−0.001 (2)	−0.001 (2)	−0.005 (3)
C2'	0.018 (3)	0.030 (4)	0.019 (3)	−0.005 (3)	0.004 (2)	−0.003 (3)
C3'	0.015 (3)	0.041 (4)	0.021 (3)	−0.002 (3)	−0.003 (2)	−0.006 (3)
C4'	0.024 (4)	0.034 (4)	0.028 (4)	−0.005 (3)	−0.009 (3)	−0.001 (3)
C5'	0.022 (3)	0.026 (4)	0.021 (3)	0.006 (3)	−0.002 (2)	0.000 (3)
C6'	0.021 (3)	0.022 (3)	0.013 (3)	0.006 (2)	−0.001 (2)	0.003 (2)
C7'	0.025 (3)	0.034 (4)	0.023 (3)	0.000 (3)	−0.001 (3)	0.004 (3)
C8'	0.030 (4)	0.049 (5)	0.032 (4)	0.015 (4)	0.007 (3)	−0.004 (4)
C9'	0.043 (5)	0.052 (5)	0.034 (4)	0.025 (4)	−0.012 (4)	−0.022 (4)
C10'	0.038 (4)	0.031 (4)	0.041 (4)	0.009 (3)	−0.016 (3)	−0.011 (3)
C11'	0.024 (3)	0.029 (4)	0.025 (3)	−0.001 (3)	−0.006 (3)	0.002 (3)
C12'	0.013 (3)	0.015 (3)	0.013 (3)	−0.004 (2)	0.002 (2)	0.000 (2)
C13'	0.018 (3)	0.017 (3)	0.016 (3)	−0.002 (2)	0.001 (2)	−0.002 (2)
C14'	0.020 (3)	0.027 (4)	0.020 (3)	0.000 (3)	−0.002 (2)	0.002 (3)
C15'	0.017 (3)	0.034 (4)	0.016 (3)	−0.006 (3)	−0.003 (2)	−0.003 (3)
C16'	0.021 (3)	0.025 (3)	0.019 (3)	−0.008 (3)	0.003 (2)	−0.012 (3)
C17'	0.014 (3)	0.017 (3)	0.017 (3)	−0.002 (2)	0.000 (2)	−0.002 (2)
C18'	0.018 (3)	0.011 (3)	0.015 (3)	0.000 (2)	0.002 (2)	−0.003 (2)
C19'	0.020 (3)	0.019 (3)	0.015 (3)	−0.001 (2)	−0.001 (2)	−0.006 (2)
C20'	0.019 (3)	0.021 (3)	0.022 (3)	−0.003 (2)	0.007 (2)	−0.007 (3)
C21'	0.025 (4)	0.016 (4)	0.029 (4)	−0.001 (3)	0.011 (3)	0.000 (3)
C22'	0.030 (4)	0.019 (3)	0.017 (3)	0.001 (3)	0.001 (3)	−0.002 (2)
C23'	0.017 (3)	0.019 (3)	0.026 (3)	0.001 (2)	−0.002 (2)	−0.007 (3)
C24'	0.013 (3)	0.014 (3)	0.021 (3)	−0.001 (2)	−0.005 (2)	−0.008 (2)
C25'	0.018 (3)	0.018 (3)	0.019 (3)	0.000 (2)	0.002 (2)	−0.004 (3)
C26'	0.019 (3)	0.027 (4)	0.025 (3)	−0.006 (3)	0.004 (2)	−0.008 (3)
C27'	0.013 (3)	0.029 (4)	0.033 (4)	0.001 (3)	−0.002 (3)	−0.013 (3)
C28'	0.018 (3)	0.021 (3)	0.029 (3)	0.005 (2)	−0.008 (3)	−0.006 (3)
C29'	0.017 (3)	0.019 (3)	0.019 (3)	−0.001 (2)	−0.002 (2)	−0.003 (2)
C30'	0.020 (3)	0.010 (3)	0.031 (4)	0.003 (3)	0.002 (3)	−0.001 (3)
C31'	0.019 (3)	0.011 (3)	0.029 (3)	−0.004 (2)	0.003 (2)	−0.007 (3)
C32'	0.020 (3)	0.023 (3)	0.029 (3)	−0.003 (2)	−0.005 (3)	−0.013 (3)
C33'	0.025 (3)	0.021 (3)	0.021 (3)	0.000 (3)	−0.002 (3)	−0.007 (3)
C34'	0.018 (3)	0.015 (3)	0.019 (3)	−0.005 (2)	0.006 (2)	−0.005 (2)
C35'	0.017 (3)	0.015 (4)	0.024 (4)	−0.002 (3)	0.006 (3)	−0.012 (3)
C36'	0.016 (3)	0.025 (4)	0.029 (4)	0.001 (3)	−0.003 (2)	−0.010 (3)
C37'	0.025 (3)	0.020 (3)	0.023 (3)	0.006 (3)	−0.002 (3)	−0.005 (3)
F1	0.025 (3)	0.035 (3)	0.051 (4)	0.001 (3)	0.001 (3)	0.002 (3)
F2	0.033 (2)	0.027 (2)	0.053 (3)	−0.0017 (19)	−0.016 (2)	−0.003 (2)
F3	0.037 (3)	0.062 (4)	0.041 (3)	−0.003 (2)	0.015 (2)	−0.031 (3)
F4	0.060 (3)	0.064 (4)	0.029 (3)	0.021 (3)	−0.021 (2)	−0.005 (2)
B1	0.019 (3)	0.024 (4)	0.015 (3)	0.003 (3)	0.000 (3)	−0.005 (3)
F1'	0.050 (4)	0.039 (3)	0.085 (5)	−0.008 (3)	0.011 (3)	0.022 (3)
F2'	0.037 (2)	0.037 (3)	0.044 (3)	0.014 (2)	−0.023 (2)	−0.008 (2)
F3'	0.060 (3)	0.048 (3)	0.035 (3)	0.016 (2)	0.015 (2)	0.006 (2)
F4'	0.097 (5)	0.078 (4)	0.071 (4)	0.050 (4)	−0.068 (4)	−0.045 (3)
B1'	0.025 (4)	0.032 (5)	0.018 (4)	0.005 (3)	−0.008 (3)	−0.004 (3)

### (4-Benzyl-1-ethyl-1,2,4-triazol-5-ylidene)[(1,2,5,6-η)-cycloocta-1,5-diene](triphenylphosphane)iridium(I) tetrafluoridoborate (5) Geometric parameters (Å, º)

**Table:**

Ir1—P1	2.3302 (15)	Ir1'—C35'	2.201 (7)
Ir1—C30	2.218 (7)	P1'—C12'	1.825 (6)
Ir1—C31	2.191 (6)	P1'—C18'	1.830 (6)
Ir1—C34	2.206 (7)	P1'—C24'	1.832 (6)
Ir1—C35	2.215 (6)	N1'—N2'	1.382 (7)
P1—C12	1.839 (6)	N1'—C1'	1.340 (8)
P1—C18	1.827 (6)	N1'—C3'	1.466 (8)
P1—C24	1.826 (6)	N2'—C2'	1.295 (9)
N1—N2	1.388 (7)	N3'—C1'	1.380 (8)
N1—C1	1.336 (8)	N3'—C2'	1.373 (8)
N1—C3	1.459 (8)	N3'—C5'	1.489 (8)
N2—C2	1.301 (8)	Ir1'—C1'	2.029 (6)
N3—C1	1.354 (8)	C2'—H2'	0.9500
N3—C2	1.382 (8)	C3'—H3'A	0.9900
N3—C5	1.473 (8)	C3'—H3'B	0.9900
Ir1—C1	2.039 (6)	C3'—C4'	1.503 (10)
C2—H2	0.9500	C4'—H4'A	0.9800
C3—H3A	0.9900	C4'—H4'B	0.9800
C3—H3B	0.9900	C4'—H4'C	0.9800
C3—C4	1.517 (9)	C5'—H5'A	0.9900
C4—H4A	0.9800	C5'—H5'B	0.9900
C4—H4B	0.9800	C5'—C6'	1.506 (9)
C4—H4C	0.9800	C6'—C7'	1.387 (9)
C5—H5A	0.9900	C6'—C11'	1.376 (10)
C5—H5B	0.9900	C7'—H7'	0.9500
C5—C6	1.512 (9)	C7'—C8'	1.388 (11)
C6—C7	1.399 (9)	C8'—H8'	0.9500
C6—C11	1.389 (9)	C8'—C9'	1.382 (13)
C7—H7	0.9500	C9'—H9'	0.9500
C7—C8	1.382 (11)	C9'—C10'	1.371 (13)
C8—H8	0.9500	C10'—H10'	0.9500
C8—C9	1.388 (12)	C10'—C11'	1.388 (10)
C9—H9	0.9500	C11'—H11'	0.9500
C9—C10	1.388 (10)	C12'—C13'	1.389 (9)
C10—H10	0.9500	C12'—C17'	1.401 (8)
C10—C11	1.400 (10)	C13'—H13'	0.9500
C11—H11	0.9500	C13'—C14'	1.393 (9)
C12—C13	1.384 (8)	C14'—H14'	0.9500
C12—C17	1.403 (9)	C14'—C15'	1.392 (10)
C13—H13	0.9500	C15'—H15'	0.9500
C13—C14	1.391 (9)	C15'—C16'	1.377 (10)
C14—H14	0.9500	C16'—H16'	0.9500
C14—C15	1.387 (9)	C16'—C17'	1.389 (8)
C15—H15	0.9500	C17'—H17'	0.9500
C15—C16	1.387 (9)	C18'—C19'	1.401 (8)
C16—H16	0.9500	C18'—C23'	1.404 (9)
C16—C17	1.390 (9)	C19'—H19'	0.9500
C17—H17	0.9500	C19'—C20'	1.387 (8)
C18—C19	1.402 (8)	C20'—H20'	0.9500
C18—C23	1.407 (8)	C20'—C21'	1.391 (10)
C19—H19	0.9500	C21'—H21'	0.9500
C19—C20	1.377 (9)	C21'—C22'	1.420 (10)
C20—H20	0.9500	C22'—H22'	0.9500
C20—C21	1.392 (10)	C22'—C23'	1.373 (9)
C21—H21	0.9500	C23'—H23'	0.9500
C21—C22	1.384 (9)	C24'—C25'	1.415 (8)
C22—H22	0.9500	C24'—C29'	1.394 (9)
C22—C23	1.388 (9)	C25'—H25'	0.9500
C23—H23	0.9500	C25'—C26'	1.389 (9)
C24—C25	1.407 (8)	C26'—H26'	0.9500
C24—C29	1.387 (8)	C26'—C27'	1.397 (10)
C25—H25	0.9500	C27'—H27'	0.9500
C25—C26	1.380 (9)	C27'—C28'	1.386 (9)
C26—H26	0.9500	C28'—H28'	0.9500
C26—C27	1.385 (9)	C28'—C29'	1.394 (9)
C27—H27	0.9500	C29'—H29'	0.9500
C27—C28	1.395 (10)	C30'—H30'	1.0000
C28—H28	0.9500	C30'—C31'	1.391 (10)
C28—C29	1.396 (8)	C30'—C37'	1.516 (9)
C29—H29	0.9500	C31'—H31'	1.0000
C30—H30	1.0000	C31'—C32'	1.501 (9)
C30—C31	1.393 (11)	C32'—H32C	0.9900
C30—C37	1.509 (11)	C32'—H32D	0.9900
C31—H31	1.0000	C32'—C33'	1.532 (9)
C31—C32	1.517 (10)	C33'—H33C	0.9900
C32—H32A	0.9900	C33'—H33D	0.9900
C32—H32B	0.9900	C33'—C34'	1.522 (8)
C32—C33	1.512 (10)	C34'—H34'	1.0000
C33—H33A	0.9900	C34'—C35'	1.403 (10)
C33—H33B	0.9900	C35'—H35'	1.0000
C33—C34	1.520 (10)	C35'—C36'	1.525 (10)
C34—H34	1.0000	C36'—H36C	0.9900
C34—C35	1.384 (10)	C36'—H36D	0.9900
C35—H35	1.0000	C36'—C37'	1.535 (9)
C35—C36	1.524 (9)	C37'—H37C	0.9900
C36—H36A	0.9900	C37'—H37D	0.9900
C36—H36B	0.9900	F1—B1	1.383 (9)
C36—C37	1.505 (10)	F2—B1	1.394 (9)
C37—H37A	0.9900	F3—B1	1.401 (8)
C37—H37B	0.9900	F4—B1	1.369 (8)
Ir1'—P1'	2.3217 (15)	F1'—B1'	1.360 (10)
Ir1'—C30'	2.195 (6)	F2'—B1'	1.401 (9)
Ir1'—C31'	2.238 (6)	F3'—B1'	1.385 (9)
Ir1'—C34'	2.175 (6)	F4'—B1'	1.366 (9)
			
N1—C1—N3	103.8 (5)	C30'—Ir1'—P1'	151.29 (18)
C1—Ir1—P1	93.14 (17)	C30'—Ir1'—C31'	36.6 (2)
C1—Ir1—C30	86.5 (3)	C30'—Ir1'—C35'	80.3 (3)
C1—Ir1—C31	90.4 (2)	C31'—Ir1'—P1'	171.78 (18)
C1—Ir1—C34	163.1 (3)	C34'—Ir1'—P1'	94.63 (16)
C1—Ir1—C35	158.0 (2)	C34'—Ir1'—C30'	95.8 (2)
C30—Ir1—P1	165.6 (2)	C34'—Ir1'—C31'	80.1 (2)
C31—Ir1—P1	157.5 (2)	C34'—Ir1'—C35'	37.4 (3)
C31—Ir1—C30	36.8 (3)	C35'—Ir1'—P1'	92.18 (19)
C31—Ir1—C34	80.2 (2)	C35'—Ir1'—C31'	87.4 (2)
C31—Ir1—C35	87.5 (2)	C12'—P1'—Ir1'	110.6 (2)
C34—Ir1—P1	90.39 (17)	C12'—P1'—C18'	102.6 (3)
C34—Ir1—C30	94.1 (3)	C12'—P1'—C24'	102.5 (3)
C34—Ir1—C35	36.5 (3)	C18'—P1'—Ir1'	116.35 (19)
C35—Ir1—P1	97.11 (16)	C18'—P1'—C24'	105.4 (3)
C35—Ir1—C30	78.8 (3)	C24'—P1'—Ir1'	117.59 (19)
C12—P1—Ir1	116.58 (18)	N2'—N1'—C3'	119.4 (5)
C18—P1—Ir1	114.38 (19)	C1'—N1'—N2'	114.4 (5)
C18—P1—C12	105.7 (3)	C1'—N1'—C3'	126.2 (5)
C24—P1—Ir1	113.3 (2)	C2'—N2'—N1'	102.9 (5)
C24—P1—C12	102.8 (3)	C1'—N3'—C5'	124.5 (5)
C24—P1—C18	102.5 (3)	C2'—N3'—C1'	107.8 (5)
N2—N1—C3	118.5 (5)	C2'—N3'—C5'	127.7 (5)
C1—N1—N2	113.7 (5)	N1'—C1'—Ir1'	123.3 (5)
C1—N1—C3	127.8 (5)	N3'—C1'—Ir1'	133.6 (5)
C2—N2—N1	102.9 (5)	N2'—C2'—N3'	112.3 (5)
C1—N3—C2	108.2 (5)	N2'—C2'—H2'	123.9
C1—N3—C5	126.0 (5)	N3'—C2'—H2'	123.9
C2—N3—C5	125.5 (5)	N1'—C3'—H3'A	109.0
N1—C1—Ir1	126.4 (4)	N1'—C3'—H3'B	109.0
N3—C1—Ir1	129.5 (4)	N1'—C3'—C4'	112.8 (6)
N2—C2—N3	111.4 (5)	H3'A—C3'—H3'B	107.8
N2—C2—H2	124.3	C4'—C3'—H3'A	109.0
N3—C2—H2	124.3	C4'—C3'—H3'B	109.0
N1—C3—H3A	109.3	C3'—C4'—H4'A	109.5
N1—C3—H3B	109.3	C3'—C4'—H4'B	109.5
N1—C3—C4	111.4 (5)	C3'—C4'—H4'C	109.5
H3A—C3—H3B	108.0	H4'A—C4'—H4'B	109.5
C4—C3—H3A	109.3	H4'A—C4'—H4'C	109.5
C4—C3—H3B	109.3	H4'B—C4'—H4'C	109.5
C3—C4—H4A	109.5	N3'—C5'—H5'A	108.8
C3—C4—H4B	109.5	N3'—C5'—H5'B	108.8
C3—C4—H4C	109.5	N3'—C5'—C6'	113.9 (5)
H4A—C4—H4B	109.5	H5'A—C5'—H5'B	107.7
H4A—C4—H4C	109.5	C6'—C5'—H5'A	108.8
H4B—C4—H4C	109.5	C6'—C5'—H5'B	108.8
N3—C5—H5A	108.9	C7'—C6'—C5'	119.9 (6)
N3—C5—H5B	108.9	C11'—C6'—C5'	120.1 (6)
N3—C5—C6	113.2 (5)	C11'—C6'—C7'	119.9 (6)
H5A—C5—H5B	107.7	C6'—C7'—H7'	120.3
C6—C5—H5A	108.9	C6'—C7'—C8'	119.5 (7)
C6—C5—H5B	108.9	C8'—C7'—H7'	120.3
C7—C6—C5	123.2 (6)	C7'—C8'—H8'	119.8
C11—C6—C5	117.7 (6)	C9'—C8'—C7'	120.4 (7)
C11—C6—C7	119.0 (6)	C9'—C8'—H8'	119.8
C6—C7—H7	119.6	C8'—C9'—H9'	120.1
C8—C7—C6	120.9 (7)	C10'—C9'—C8'	119.8 (7)
C8—C7—H7	119.6	C10'—C9'—H9'	120.1
C7—C8—H8	119.8	C9'—C10'—H10'	119.9
C7—C8—C9	120.4 (7)	C9'—C10'—C11'	120.3 (7)
C9—C8—H8	119.8	C11'—C10'—H10'	119.9
C8—C9—H9	120.4	C6'—C11'—C10'	120.2 (7)
C8—C9—C10	119.2 (7)	C6'—C11'—H11'	119.9
C10—C9—H9	120.4	C10'—C11'—H11'	119.9
C9—C10—H10	119.6	C13'—C12'—P1'	121.2 (4)
C9—C10—C11	120.7 (7)	C13'—C12'—C17'	119.4 (5)
C11—C10—H10	119.6	C17'—C12'—P1'	119.2 (5)
C6—C11—C10	119.9 (7)	C12'—C13'—H13'	119.8
C6—C11—H11	120.1	C12'—C13'—C14'	120.4 (6)
C10—C11—H11	120.1	C14'—C13'—H13'	119.8
C13—C12—P1	124.4 (5)	C13'—C14'—H14'	120.3
C13—C12—C17	118.5 (5)	C15'—C14'—C13'	119.4 (6)
C17—C12—P1	117.0 (4)	C15'—C14'—H14'	120.3
C12—C13—H13	119.6	C14'—C15'—H15'	119.7
C12—C13—C14	120.8 (6)	C16'—C15'—C14'	120.7 (6)
C14—C13—H13	119.6	C16'—C15'—H15'	119.7
C13—C14—H14	119.9	C15'—C16'—H16'	120.0
C15—C14—C13	120.2 (6)	C15'—C16'—C17'	120.0 (6)
C15—C14—H14	119.9	C17'—C16'—H16'	120.0
C14—C15—H15	120.0	C12'—C17'—H17'	120.0
C14—C15—C16	119.9 (6)	C16'—C17'—C12'	120.1 (6)
C16—C15—H15	120.0	C16'—C17'—H17'	120.0
C15—C16—H16	120.2	C19'—C18'—P1'	121.5 (5)
C15—C16—C17	119.6 (6)	C19'—C18'—C23'	117.9 (5)
C17—C16—H16	120.2	C23'—C18'—P1'	120.4 (4)
C12—C17—H17	119.6	C18'—C19'—H19'	119.3
C16—C17—C12	120.9 (6)	C20'—C19'—C18'	121.5 (6)
C16—C17—H17	119.6	C20'—C19'—H19'	119.3
C19—C18—P1	118.7 (4)	C19'—C20'—H20'	119.8
C19—C18—C23	118.7 (6)	C19'—C20'—C21'	120.5 (6)
C23—C18—P1	122.5 (4)	C21'—C20'—H20'	119.8
C18—C19—H19	119.8	C20'—C21'—H21'	120.9
C20—C19—C18	120.4 (6)	C20'—C21'—C22'	118.3 (6)
C20—C19—H19	119.8	C22'—C21'—H21'	120.9
C19—C20—H20	119.7	C21'—C22'—H22'	119.6
C19—C20—C21	120.6 (6)	C23'—C22'—C21'	120.8 (6)
C21—C20—H20	119.7	C23'—C22'—H22'	119.6
C20—C21—H21	120.2	C18'—C23'—H23'	119.5
C22—C21—C20	119.6 (6)	C22'—C23'—C18'	121.0 (6)
C22—C21—H21	120.2	C22'—C23'—H23'	119.5
C21—C22—H22	119.8	C25'—C24'—P1'	116.2 (5)
C21—C22—C23	120.4 (6)	C29'—C24'—P1'	125.6 (5)
C23—C22—H22	119.8	C29'—C24'—C25'	118.2 (5)
C18—C23—H23	119.9	C24'—C25'—H25'	119.6
C22—C23—C18	120.1 (6)	C26'—C25'—C24'	120.7 (6)
C22—C23—H23	119.9	C26'—C25'—H25'	119.6
C25—C24—P1	120.1 (4)	C25'—C26'—H26'	119.9
C29—C24—P1	120.6 (4)	C25'—C26'—C27'	120.2 (6)
C29—C24—C25	119.1 (5)	C27'—C26'—H26'	119.9
C24—C25—H25	119.8	C26'—C27'—H27'	120.3
C26—C25—C24	120.5 (6)	C28'—C27'—C26'	119.3 (6)
C26—C25—H25	119.8	C28'—C27'—H27'	120.3
C25—C26—H26	120.1	C27'—C28'—H28'	119.6
C25—C26—C27	119.9 (6)	C27'—C28'—C29'	120.7 (6)
C27—C26—H26	120.1	C29'—C28'—H28'	119.6
C26—C27—H27	119.7	C24'—C29'—C28'	120.8 (6)
C26—C27—C28	120.6 (6)	C24'—C29'—H29'	119.6
C28—C27—H27	119.7	C28'—C29'—H29'	119.6
C27—C28—H28	120.4	Ir1'—C30'—H30'	114.1
C27—C28—C29	119.3 (6)	C31'—C30'—Ir1'	73.4 (4)
C29—C28—H28	120.4	C31'—C30'—H30'	114.1
C24—C29—C28	120.6 (6)	C31'—C30'—C37'	124.5 (6)
C24—C29—H29	119.7	C37'—C30'—Ir1'	109.5 (4)
C28—C29—H29	119.7	C37'—C30'—H30'	114.1
Ir1—C30—H30	113.6	Ir1'—C31'—H31'	114.1
C31—C30—Ir1	70.5 (4)	C30'—C31'—Ir1'	70.0 (4)
C31—C30—H30	113.6	C30'—C31'—H31'	114.1
C31—C30—C37	126.7 (7)	C30'—C31'—C32'	124.2 (6)
C37—C30—Ir1	110.4 (5)	C32'—C31'—Ir1'	112.3 (4)
C37—C30—H30	113.6	C32'—C31'—H31'	114.1
Ir1—C31—H31	114.0	C31'—C32'—H32C	109.0
C30—C31—Ir1	72.6 (4)	C31'—C32'—H32D	109.0
C30—C31—H31	114.0	C31'—C32'—C33'	112.9 (5)
C30—C31—C32	122.6 (7)	H32C—C32'—H32D	107.8
C32—C31—Ir1	112.8 (4)	C33'—C32'—H32C	109.0
C32—C31—H31	114.0	C33'—C32'—H32D	109.0
C31—C32—H32A	108.7	C32'—C33'—H33C	108.9
C31—C32—H32B	108.7	C32'—C33'—H33D	108.9
H32A—C32—H32B	107.6	H33C—C33'—H33D	107.7
C33—C32—C31	114.3 (6)	C34'—C33'—C32'	113.5 (5)
C33—C32—H32A	108.7	C34'—C33'—H33C	108.9
C33—C32—H32B	108.7	C34'—C33'—H33D	108.9
C32—C33—H33A	108.8	Ir1'—C34'—H34'	114.0
C32—C33—H33B	108.8	C33'—C34'—Ir1'	109.5 (4)
C32—C33—C34	113.6 (6)	C33'—C34'—H34'	114.0
H33A—C33—H33B	107.7	C35'—C34'—Ir1'	72.3 (4)
C34—C33—H33A	108.8	C35'—C34'—C33'	125.3 (6)
C34—C33—H33B	108.8	C35'—C34'—H34'	114.0
Ir1—C34—H34	114.1	Ir1'—C35'—H35'	113.9
C33—C34—Ir1	109.4 (4)	C34'—C35'—Ir1'	70.3 (4)
C33—C34—H34	114.1	C34'—C35'—H35'	113.9
C35—C34—Ir1	72.1 (4)	C34'—C35'—C36'	123.8 (6)
C35—C34—C33	125.2 (6)	C36'—C35'—Ir1'	113.5 (5)
C35—C34—H34	114.1	C36'—C35'—H35'	113.9
Ir1—C35—H35	113.9	C35'—C36'—H36C	109.2
C34—C35—Ir1	71.4 (4)	C35'—C36'—H36D	109.2
C34—C35—H35	113.9	C35'—C36'—C37'	112.0 (5)
C34—C35—C36	123.0 (6)	H36C—C36'—H36D	107.9
C36—C35—Ir1	113.6 (4)	C37'—C36'—H36C	109.2
C36—C35—H35	113.9	C37'—C36'—H36D	109.2
C35—C36—H36A	109.0	C30'—C37'—C36'	114.1 (5)
C35—C36—H36B	109.0	C30'—C37'—H37C	108.7
H36A—C36—H36B	107.8	C30'—C37'—H37D	108.7
C37—C36—C35	113.0 (6)	C36'—C37'—H37C	108.7
C37—C36—H36A	109.0	C36'—C37'—H37D	108.7
C37—C36—H36B	109.0	H37C—C37'—H37D	107.6
C30—C37—H37A	108.8	F1—B1—F2	109.6 (6)
C30—C37—H37B	108.8	F1—B1—F3	108.5 (6)
C36—C37—C30	113.7 (6)	F2—B1—F3	108.1 (6)
C36—C37—H37A	108.8	F4—B1—F1	111.6 (6)
C36—C37—H37B	108.8	F4—B1—F2	109.6 (6)
H37A—C37—H37B	107.7	F4—B1—F3	109.3 (6)
N1'—C1'—N3'	102.7 (5)	F1'—B1'—F2'	109.3 (6)
C1'—Ir1'—P1'	94.64 (18)	F1'—B1'—F3'	108.5 (7)
C1'—Ir1'—C30'	90.2 (3)	F1'—B1'—F4'	110.6 (7)
C1'—Ir1'—C31'	86.9 (2)	F3'—B1'—F2'	109.5 (6)
C1'—Ir1'—C34'	148.7 (2)	F4'—B1'—F2'	109.3 (6)
C1'—Ir1'—C35'	169.9 (3)	F4'—B1'—F3'	109.6 (6)
			
Ir1—P1—C12—C13	−110.1 (5)	Ir1'—P1'—C12'—C13'	−19.5 (5)
Ir1—P1—C12—C17	66.7 (5)	Ir1'—P1'—C12'—C17'	164.4 (4)
Ir1—P1—C18—C19	66.1 (5)	Ir1'—P1'—C18'—C19'	103.8 (5)
Ir1—P1—C18—C23	−110.2 (5)	Ir1'—P1'—C18'—C23'	−71.3 (5)
Ir1—P1—C24—C25	30.9 (5)	Ir1'—P1'—C24'—C25'	−59.7 (5)
Ir1—P1—C24—C29	−154.9 (4)	Ir1'—P1'—C24'—C29'	119.4 (5)
Ir1—C30—C31—C32	−106.4 (6)	Ir1'—C30'—C31'—C32'	−103.9 (6)
Ir1—C30—C37—C36	36.2 (8)	Ir1'—C30'—C37'—C36'	36.4 (7)
Ir1—C31—C32—C33	8.6 (8)	Ir1'—C31'—C32'—C33'	12.9 (7)
Ir1—C34—C35—C36	−106.6 (6)	Ir1'—C34'—C35'—C36'	−105.6 (7)
Ir1—C35—C36—C37	12.0 (8)	Ir1'—C35'—C36'—C37'	11.5 (7)
P1—C12—C13—C14	175.5 (5)	P1'—C12'—C13'—C14'	−176.5 (4)
P1—C12—C17—C16	−175.6 (5)	P1'—C12'—C17'—C16'	175.2 (4)
P1—C18—C19—C20	−179.5 (5)	P1'—C18'—C19'—C20'	−177.0 (5)
P1—C18—C23—C22	177.3 (5)	P1'—C18'—C23'—C22'	178.8 (5)
P1—C24—C25—C26	172.0 (5)	P1'—C24'—C25'—C26'	175.6 (5)
P1—C24—C29—C28	−174.0 (5)	P1'—C24'—C29'—C28'	−176.5 (5)
N1—N2—C2—N3	−0.3 (6)	N1'—N2'—C2'—N3'	−1.8 (7)
N2—N1—C1—Ir1	175.2 (4)	N2'—N1'—C1'—Ir1'	−174.4 (4)
N2—N1—C1—N3	1.4 (6)	N2'—N1'—C1'—N3'	−0.9 (7)
N2—N1—C3—C4	69.9 (7)	N2'—N1'—C3'—C4'	−66.8 (8)
N3—C5—C6—C7	43.3 (8)	N3'—C5'—C6'—C7'	−74.6 (8)
N3—C5—C6—C11	−140.9 (6)	N3'—C5'—C6'—C11'	108.8 (7)
C1—N1—N2—C2	−0.7 (6)	C1'—N1'—N2'—C2'	1.7 (7)
C1—N1—C3—C4	−109.0 (7)	C1'—N1'—C3'—C4'	112.3 (7)
C1—N3—C2—N2	1.2 (7)	C1'—N3'—C2'—N2'	1.4 (8)
C1—N3—C5—C6	−132.0 (6)	C1'—N3'—C5'—C6'	−127.9 (6)
C2—N3—C1—Ir1	−175.0 (4)	C2'—N3'—C1'—Ir1'	172.2 (5)
C2—N3—C1—N1	−1.5 (6)	C2'—N3'—C1'—N1'	−0.2 (7)
C2—N3—C5—C6	54.5 (8)	C2'—N3'—C5'—C6'	54.4 (9)
C3—N1—N2—C2	−179.7 (5)	C3'—N1'—N2'—C2'	−179.1 (6)
C3—N1—C1—Ir1	−5.9 (9)	C3'—N1'—C1'—Ir1'	6.5 (9)
C3—N1—C1—N3	−179.7 (5)	C3'—N1'—C1'—N3'	−180.0 (6)
C5—N3—C1—Ir1	10.5 (8)	C5'—N3'—C1'—Ir1'	−5.9 (10)
C5—N3—C1—N1	−175.9 (5)	C5'—N3'—C1'—N1'	−178.3 (6)
C5—N3—C2—N2	175.7 (5)	C5'—N3'—C2'—N2'	179.4 (6)
C5—C6—C7—C8	175.0 (7)	C5'—C6'—C7'—C8'	−177.3 (6)
C5—C6—C11—C10	−175.1 (7)	C5'—C6'—C11'—C10'	177.3 (6)
C6—C7—C8—C9	0.1 (12)	C6'—C7'—C8'—C9'	0.2 (11)
C7—C6—C11—C10	0.9 (10)	C7'—C6'—C11'—C10'	0.7 (10)
C7—C8—C9—C10	0.5 (12)	C7'—C8'—C9'—C10'	0.2 (12)
C8—C9—C10—C11	−0.3 (12)	C8'—C9'—C10'—C11'	−0.2 (12)
C9—C10—C11—C6	−0.4 (11)	C9'—C10'—C11'—C6'	−0.3 (11)
C11—C6—C7—C8	−0.7 (10)	C11'—C6'—C7'—C8'	−0.7 (10)
C12—P1—C18—C19	−63.5 (5)	C12'—P1'—C18'—C19'	−17.1 (5)
C12—P1—C18—C23	120.1 (5)	C12'—P1'—C18'—C23'	167.9 (5)
C12—P1—C24—C25	157.6 (5)	C12'—P1'—C24'—C25'	61.7 (5)
C12—P1—C24—C29	−28.2 (5)	C12'—P1'—C24'—C29'	−119.2 (5)
C12—C13—C14—C15	−0.6 (9)	C12'—C13'—C14'—C15'	0.5 (9)
C13—C12—C17—C16	1.4 (8)	C13'—C12'—C17'—C16'	−1.1 (8)
C13—C14—C15—C16	2.4 (9)	C13'—C14'—C15'—C16'	0.8 (9)
C14—C15—C16—C17	−2.1 (9)	C14'—C15'—C16'—C17'	−2.2 (9)
C15—C16—C17—C12	0.2 (9)	C15'—C16'—C17'—C12'	2.3 (9)
C17—C12—C13—C14	−1.2 (8)	C17'—C12'—C13'—C14'	−0.3 (9)
C18—P1—C12—C13	18.2 (5)	C18'—P1'—C12'—C13'	105.3 (5)
C18—P1—C12—C17	−165.0 (4)	C18'—P1'—C12'—C17'	−70.9 (5)
C18—P1—C24—C25	−92.8 (5)	C18'—P1'—C24'—C25'	168.8 (4)
C18—P1—C24—C29	81.4 (5)	C18'—P1'—C24'—C29'	−12.2 (6)
C18—C19—C20—C21	2.6 (9)	C18'—C19'—C20'—C21'	0.1 (9)
C19—C18—C23—C22	0.9 (9)	C19'—C18'—C23'—C22'	3.6 (9)
C19—C20—C21—C22	−0.2 (9)	C19'—C20'—C21'—C22'	0.0 (10)
C20—C21—C22—C23	−1.9 (9)	C20'—C21'—C22'—C23'	1.7 (10)
C21—C22—C23—C18	1.5 (9)	C21'—C22'—C23'—C18'	−3.6 (10)
C23—C18—C19—C20	−3.0 (9)	C23'—C18'—C19'—C20'	−1.8 (9)
C24—P1—C12—C13	125.3 (5)	C24'—P1'—C12'—C13'	−145.6 (5)
C24—P1—C12—C17	−57.9 (5)	C24'—P1'—C12'—C17'	38.2 (5)
C24—P1—C18—C19	−170.9 (5)	C24'—P1'—C18'—C19'	−124.0 (5)
C24—P1—C18—C23	12.7 (5)	C24'—P1'—C18'—C23'	61.0 (5)
C24—C25—C26—C27	1.9 (10)	C24'—C25'—C26'—C27'	2.0 (9)
C25—C24—C29—C28	0.3 (9)	C25'—C24'—C29'—C28'	2.5 (9)
C25—C26—C27—C28	0.5 (10)	C25'—C26'—C27'—C28'	0.6 (9)
C26—C27—C28—C29	−2.5 (10)	C26'—C27'—C28'—C29'	−1.6 (9)
C27—C28—C29—C24	2.1 (10)	C27'—C28'—C29'—C24'	0.0 (9)
C29—C24—C25—C26	−2.3 (9)	C29'—C24'—C25'—C26'	−3.5 (9)
C30—C31—C32—C33	92.0 (8)	C30'—C31'—C32'—C33'	93.2 (8)
C31—C30—C37—C36	−44.2 (10)	C31'—C30'—C37'—C36'	−46.5 (9)
C31—C32—C33—C34	−29.2 (9)	C31'—C32'—C33'—C34'	−33.8 (8)
C32—C33—C34—Ir1	34.7 (7)	C32'—C33'—C34'—Ir1'	37.9 (6)
C32—C33—C34—C35	−46.6 (9)	C32'—C33'—C34'—C35'	−43.7 (8)
C33—C34—C35—Ir1	101.4 (6)	C33'—C34'—C35'—Ir1'	101.8 (6)
C33—C34—C35—C36	−5.1 (10)	C33'—C34'—C35'—C36'	−3.9 (10)
C34—C35—C36—C37	94.5 (8)	C34'—C35'—C36'—C37'	92.8 (8)
C35—C36—C37—C30	−32.0 (9)	C35'—C36'—C37'—C30'	−31.9 (8)
C37—C30—C31—Ir1	101.4 (7)	C37'—C30'—C31'—Ir1'	102.5 (6)
C37—C30—C31—C32	−5.0 (11)	C37'—C30'—C31'—C32'	−1.4 (10)

### (4-Benzyl-1-ethyl-1,2,4-triazol-5-ylidene)[(1,2,5,6-η)-cycloocta-1,5-diene](triphenylphosphane)iridium(I) tetrafluoridoborate (5) Hydrogen-bond geometry (Å, º)

**Table:**

*D*—H···*A*	*D*—H	H···*A*	*D*···*A*	*D*—H···*A*
C5—H5*A*···F2	0.99	2.52	3.488 (8)	167
C8—H8···F2^i^	0.95	2.51	3.262 (10)	137
C14—H14···F4′	0.95	2.40	3.250 (8)	149
C16—H16···F3	0.95	2.74	3.487 (9)	136
C21—H21···F3′^ii^	0.95	2.52	3.413 (8)	157
C22—H22···F4′^ii^	0.95	2.62	3.396 (8)	139
C33—H33*A*···F1′^iii^	0.99	2.51	3.378 (9)	146
C37—H37*B*···F2′^i^	0.99	2.38	3.313 (9)	158
C2′—H2′···F1^iv^	0.95	2.37	3.274 (9)	160
C16′—H16′···F2^v^	0.95	2.59	3.388 (7)	142
C20′—H20′···F3′^vi^	0.95	2.71	3.496 (8)	141
C28′—H28′···F3′	0.95	2.45	3.335 (8)	154
C32′—H32*D*···F2′^ii^	0.99	2.44	3.425 (7)	176
C37′—H37*C*···F4^vii^	0.99	2.51	3.375 (8)	146

Symmetry codes: (i) *x*+1, *y*, *z*; (ii) *x*, *y*+1, *z*; (iii) *x*+1, *y*+1, *z*; (iv) *x*, *y*+1, *z*−1; (v) *x*−1, *y*, *z*−1; (vi) *x*−1, *y*, *z*; (vii) *x*−1, *y*+1, *z*−1.

### (4-Benzyl-1-ethyl-1,2,4-triazol-5-ylidene)[(1,2,5,6-η)-cycloocta-1,5-diene](tricyclohexylphosphane)iridium(I) tetrafluoridoborate dicholoromethane sesquisolvate (6) Crystal data

**Table:**

[Ir(C_8_H_12_)(C_11_H_13_N_3_)(C_18_H_33_P)]BF_4_·1.5CH_2_Cl_2_	*F*(000) = 1988
*M**_r_* = 982.23	*D*_x_ = 1.572 Mg m^−^^3^
Monoclinic, *P*2_1_/*c*	Mo *K*α radiation, λ = 0.71073 Å
*a* = 12.1281 (2) Å	Cell parameters from 35970 reflections
*b* = 14.4399 (2) Å	θ = 2.2–28.2°
*c* = 23.7057 (3) Å	µ = 3.50 mm^−^^1^
β = 92.016 (1)°	*T* = 100 K
*V* = 4148.98 (10) Å^3^	Plate, orange
*Z* = 4	0.27 × 0.1 × 0.01 mm

### (4-Benzyl-1-ethyl-1,2,4-triazol-5-ylidene)[(1,2,5,6-η)-cycloocta-1,5-diene](tricyclohexylphosphane)iridium(I) tetrafluoridoborate dicholoromethane sesquisolvate (6) Data collection

**Table:**

Rigaku XtaLAB Synergy-S diffractometer	8797 reflections with *I* > 2σ(*I*)
Detector resolution: 10.0000 pixels mm^-1^	*R*_int_ = 0.051
ω scans	θ_max_ = 28.3°, θ_min_ = 1.7°
Absorption correction: multi-scan (SCALE3 ABSPACK; Rigaku OD, 2024)	*h* = −16→16
*T*_min_ = 0.642, *T*_max_ = 1.000	*k* = −19→19
88352 measured reflections	*l* = −31→31
10279 independent reflections	

### (4-Benzyl-1-ethyl-1,2,4-triazol-5-ylidene)[(1,2,5,6-η)-cycloocta-1,5-diene](tricyclohexylphosphane)iridium(I) tetrafluoridoborate dicholoromethane sesquisolvate (6) Refinement

**Table:**

Refinement on *F*^2^	309 restraints
Least-squares matrix: full	Hydrogen site location: inferred from neighbouring sites
*R*[*F*^2^ > 2σ(*F*^2^)] = 0.036	H-atom parameters constrained
*wR*(*F*^2^) = 0.078	*w* = 1/[σ^2^(*F*_o_^2^) + (0.0263*P*)^2^ + 17.4212*P*] where *P* = (*F*_o_^2^ + 2*F*_c_^2^)/3
*S* = 1.05	(Δ/σ)_max_ = 0.001
10279 reflections	Δρ_max_ = 1.90 e Å^−^^3^
575 parameters	Δρ_min_ = −1.29 e Å^−^^3^

### (4-Benzyl-1-ethyl-1,2,4-triazol-5-ylidene)[(1,2,5,6-η)-cycloocta-1,5-diene](tricyclohexylphosphane)iridium(I) tetrafluoridoborate dicholoromethane sesquisolvate (6) Special details

**Table:**

Geometry. All esds (except the esd in the dihedral angle between two l.s. planes) are estimated using the full covariance matrix. The cell esds are taken into account individually in the estimation of esds in distances, angles and torsion angles; correlations between esds in cell parameters are only used when they are defined by crystal symmetry. An approximate (isotropic) treatment of cell esds is used for estimating esds involving l.s. planes.

### (4-Benzyl-1-ethyl-1,2,4-triazol-5-ylidene)[(1,2,5,6-η)-cycloocta-1,5-diene](tricyclohexylphosphane)iridium(I) tetrafluoridoborate dicholoromethane sesquisolvate (6) Fractional atomic coordinates and isotropic or equivalent isotropic displacement parameters (Å^2^)

**Table:**

	*x*	*y*	*z*	*U*_iso_*/*U*_eq_	Occ. (<1)
Ir1	0.21581 (2)	0.31646 (2)	0.37651 (2)	0.01583 (5)	
P1	0.33938 (7)	0.18989 (6)	0.38680 (4)	0.01495 (17)	
N1	0.3216 (3)	0.4014 (2)	0.27119 (13)	0.0227 (7)	
N2	0.4035 (3)	0.4605 (2)	0.25436 (14)	0.0301 (8)	
N3	0.4030 (3)	0.4466 (2)	0.34670 (13)	0.0210 (6)	
C1	0.3193 (3)	0.3899 (3)	0.32772 (15)	0.0192 (7)	
C2	0.4509 (4)	0.4867 (3)	0.30157 (17)	0.0293 (9)	
H2	0.511401	0.528432	0.304371	0.035*	
C3	0.2533 (4)	0.3569 (3)	0.22702 (16)	0.0315 (9)	
H3A	0.200399	0.314360	0.244518	0.038*	
H3B	0.300865	0.319735	0.202541	0.038*	
C4	0.1904 (5)	0.4281 (4)	0.19135 (19)	0.0439 (12)	
H4A	0.148177	0.396546	0.161007	0.066*	
H4B	0.242559	0.471392	0.174957	0.066*	
H4C	0.139734	0.462180	0.215080	0.066*	
C5	0.4356 (3)	0.4623 (3)	0.40607 (16)	0.0242 (8)	
H5A	0.514643	0.479434	0.408434	0.029*	
H5B	0.427068	0.403652	0.427060	0.029*	
C6	0.3703 (3)	0.5367 (3)	0.43438 (15)	0.0211 (7)	
C7	0.3432 (3)	0.5250 (3)	0.49038 (17)	0.0279 (9)	
H7	0.362911	0.469157	0.509416	0.033*	
C8	0.2878 (4)	0.5939 (3)	0.51890 (18)	0.0337 (10)	
H8	0.270769	0.585469	0.557370	0.040*	
C9	0.2577 (4)	0.6741 (3)	0.4914 (2)	0.0351 (10)	
H9	0.219403	0.721144	0.510702	0.042*	
C10	0.2832 (4)	0.6865 (3)	0.4353 (2)	0.0336 (9)	
H10	0.262083	0.741927	0.416237	0.040*	
C11	0.3392 (4)	0.6185 (3)	0.40718 (18)	0.0299 (9)	
H11	0.356820	0.627562	0.368838	0.036*	
C12	0.2774 (3)	0.0849 (2)	0.35419 (15)	0.0187 (7)	
H12	0.218136	0.066590	0.380270	0.022*	
C13	0.2167 (3)	0.1003 (3)	0.29744 (16)	0.0230 (8)	
H13A	0.270665	0.113072	0.268042	0.028*	
H13B	0.167113	0.154444	0.299931	0.028*	
C14	0.1491 (4)	0.0138 (3)	0.28133 (19)	0.0327 (10)	
H14A	0.090559	0.004919	0.308866	0.039*	
H14B	0.113123	0.022984	0.243615	0.039*	
C15	0.2215 (4)	−0.0723 (3)	0.28034 (19)	0.0359 (10)	
H15A	0.174738	−0.127276	0.272340	0.043*	
H15B	0.274383	−0.066538	0.249648	0.043*	
C16	0.2850 (4)	−0.0861 (3)	0.33630 (18)	0.0289 (9)	
H16A	0.334453	−0.140276	0.333393	0.035*	
H16B	0.232324	−0.099012	0.366297	0.035*	
C17	0.3532 (3)	−0.0006 (2)	0.35230 (16)	0.0217 (8)	
H17A	0.410576	0.009290	0.324230	0.026*	
H17B	0.390423	−0.010042	0.389696	0.026*	
C18	0.3798 (3)	0.1479 (3)	0.45886 (15)	0.0197 (7)	
H18	0.444398	0.106044	0.453760	0.024*	
C19	0.2941 (4)	0.0891 (3)	0.48899 (16)	0.0261 (8)	
H19A	0.228003	0.127233	0.495834	0.031*	
H19B	0.271095	0.036429	0.464584	0.031*	
C20	0.3420 (4)	0.0525 (3)	0.54528 (17)	0.0311 (9)	
H20A	0.284462	0.017373	0.564769	0.037*	
H20B	0.403590	0.009561	0.538060	0.037*	
C21	0.3837 (4)	0.1310 (3)	0.58314 (17)	0.0330 (10)	
H21A	0.417146	0.104896	0.618368	0.040*	
H21B	0.320824	0.170566	0.593374	0.040*	
C22	0.4689 (4)	0.1896 (3)	0.55380 (17)	0.0313 (9)	
H22A	0.535277	0.151610	0.547376	0.038*	
H22B	0.491169	0.242113	0.578480	0.038*	
C23	0.4230 (3)	0.2269 (3)	0.49704 (16)	0.0249 (8)	
H23A	0.481883	0.260833	0.477804	0.030*	
H23B	0.362329	0.270966	0.503804	0.030*	
C24	0.4754 (3)	0.2124 (3)	0.35529 (17)	0.0219 (8)	
H24	0.491806	0.279085	0.363436	0.026*	
C25	0.5777 (3)	0.1588 (3)	0.3783 (2)	0.0311 (10)	
H25A	0.583308	0.164329	0.419876	0.037*	
H25B	0.569971	0.092312	0.368593	0.037*	
C26	0.6819 (4)	0.1976 (3)	0.3527 (3)	0.0476 (14)	
H26A	0.693091	0.262094	0.365811	0.057*	
H26B	0.746241	0.160650	0.366248	0.057*	
C27	0.6760 (4)	0.1963 (3)	0.2888 (3)	0.0535 (16)	
H27A	0.742022	0.227405	0.274380	0.064*	
H27B	0.676260	0.131309	0.275510	0.064*	
C28	0.5735 (4)	0.2443 (3)	0.2654 (2)	0.0446 (13)	
H28A	0.568697	0.236604	0.223883	0.053*	
H28B	0.578451	0.311400	0.273714	0.053*	
C29	0.4697 (4)	0.2049 (3)	0.29090 (17)	0.0282 (9)	
H29A	0.461360	0.139050	0.279883	0.034*	
H29B	0.404337	0.239018	0.275810	0.034*	
C30	0.0635 (9)	0.3834 (9)	0.3417 (4)	0.0303 (15)	0.5
H30	0.069812	0.405199	0.301934	0.036*	0.5
C30*	0.0852 (9)	0.3941 (10)	0.3353 (4)	0.0307 (14)	0.5
H30*	0.105282	0.423606	0.298908	0.037*	0.5
C31	0.1152 (3)	0.4433 (3)	0.38211 (17)	0.0288 (8)	
H31A	0.158498	0.499667	0.372402	0.035*	0.5
H31	0.148745	0.500774	0.366617	0.035*	0.5
C32	0.0628 (4)	0.4535 (3)	0.43806 (18)	0.0339 (9)	
H32A	0.104181	0.501268	0.460024	0.041*	0.5
H32B	−0.013164	0.477086	0.431268	0.041*	0.5
H32C	−0.018327	0.453879	0.431754	0.041*	0.5
H32D	0.084311	0.514332	0.454281	0.041*	0.5
C33	0.0568 (8)	0.3681 (5)	0.4736 (4)	0.0316 (15)	0.5
H33A	−0.020982	0.347405	0.474243	0.038*	0.5
H33B	0.080735	0.383742	0.512832	0.038*	0.5
C33*	0.0932 (8)	0.3774 (5)	0.4817 (4)	0.0293 (15)	0.5
H33C	0.154532	0.399535	0.506885	0.035*	0.5
H33D	0.028942	0.365115	0.505229	0.035*	0.5
C34	0.1275 (3)	0.2878 (3)	0.45318 (16)	0.0267 (8)	
H34	0.170413	0.253616	0.483341	0.032*	0.5
H34A	0.173816	0.249171	0.479784	0.032*	0.5
C35	0.0816 (3)	0.2343 (3)	0.41109 (18)	0.0321 (10)	
H35A	0.091276	0.165607	0.413710	0.038*	0.5
H35	0.108320	0.169255	0.416568	0.038*	0.5
C36	−0.0251 (7)	0.2316 (7)	0.3774 (4)	0.0376 (16)	0.5
H36A	−0.086799	0.216732	0.402073	0.045*	0.5
H36B	−0.022148	0.183813	0.347527	0.045*	0.5
C36*	−0.0296 (7)	0.2727 (7)	0.3843 (4)	0.0339 (15)	0.5
H36C	−0.077302	0.219499	0.373593	0.041*	0.5
H36D	−0.067682	0.308199	0.413636	0.041*	0.5
C37	−0.0414 (7)	0.3268 (7)	0.3513 (4)	0.0337 (14)	0.5
H37A	−0.089526	0.363200	0.375832	0.040*	0.5
H37B	−0.081411	0.319148	0.314410	0.040*	0.5
C37*	−0.0186 (8)	0.3357 (7)	0.3318 (4)	0.0332 (14)	0.5
H37C	−0.017682	0.296655	0.297552	0.040*	0.5
H37D	−0.083694	0.376985	0.328391	0.040*	0.5
Cl1	−0.0914 (3)	0.1650 (2)	0.20050 (15)	0.0913 (9)	0.7
Cl2	−0.0151 (3)	0.2836 (3)	0.11033 (19)	0.0877 (11)	0.7
C38	−0.0202 (16)	0.1727 (11)	0.1378 (7)	0.0888 (13)	0.7
H38A	0.056181	0.150162	0.144868	0.107*	0.7
H38B	−0.056037	0.131354	0.109325	0.107*	0.7
Cl1*	−0.0146 (9)	0.3008 (7)	0.0811 (4)	0.0856 (17)	0.3
Cl2*	−0.0496 (6)	0.2152 (6)	0.1931 (4)	0.0932 (14)	0.3
C38*	−0.019 (4)	0.192 (3)	0.1232 (17)	0.0886 (14)	0.3
H38C	−0.075391	0.149852	0.106375	0.106*	0.3
H38D	0.053563	0.160375	0.122133	0.106*	0.3
Cl3	0.0397 (15)	0.5163 (8)	0.0662 (5)	0.102 (3)	0.5
Cl4	−0.0172 (15)	0.5042 (9)	−0.0541 (5)	0.097 (3)	0.5
C39	0.0466 (13)	0.5722 (9)	−0.0003 (5)	0.097 (3)	0.5
H39A	0.009374	0.633141	0.001291	0.117*	0.5
H39B	0.124731	0.582964	−0.009141	0.117*	0.5
F1	0.2168 (4)	0.1568 (4)	0.1635 (2)	0.0626 (15)	0.7
F1*	0.2650 (10)	0.1739 (8)	0.1705 (5)	0.064 (3)	0.3
F2	0.3946 (4)	0.1517 (3)	0.1611 (2)	0.0568 (11)	0.7
F2*	0.3989 (6)	0.1141 (8)	0.1179 (5)	0.070 (2)	0.3
F3	0.2972 (6)	0.1329 (4)	0.08020 (18)	0.0702 (15)	0.7
F3*	0.2328 (9)	0.1246 (10)	0.0839 (3)	0.066 (3)	0.3
F4	0.3022 (5)	0.0209 (3)	0.1453 (3)	0.0583 (15)	0.7
F4*	0.2671 (12)	0.0243 (6)	0.1505 (7)	0.064 (3)	0.3
B1	0.2955 (4)	0.1124 (3)	0.1342 (2)	0.0510 (13)	

### (4-Benzyl-1-ethyl-1,2,4-triazol-5-ylidene)[(1,2,5,6-η)-cycloocta-1,5-diene](tricyclohexylphosphane)iridium(I) tetrafluoridoborate dicholoromethane sesquisolvate (6) Atomic displacement parameters (Å^2^)

**Table:**

	*U*^11^	*U*^22^	*U*^33^	*U*^12^	*U*^13^	*U*^23^
Ir1	0.01694 (7)	0.01633 (7)	0.01429 (7)	0.00267 (6)	0.00145 (4)	−0.00031 (6)
P1	0.0168 (4)	0.0139 (4)	0.0144 (4)	0.0006 (3)	0.0026 (3)	−0.0001 (3)
N1	0.0325 (18)	0.0183 (16)	0.0174 (15)	0.0010 (13)	0.0024 (13)	0.0020 (12)
N2	0.042 (2)	0.0244 (18)	0.0245 (17)	−0.0050 (16)	0.0073 (15)	0.0052 (14)
N3	0.0249 (16)	0.0183 (15)	0.0199 (15)	−0.0012 (13)	0.0008 (12)	0.0007 (12)
C1	0.0238 (19)	0.0172 (17)	0.0166 (16)	0.0068 (14)	0.0012 (14)	0.0004 (14)
C2	0.038 (2)	0.021 (2)	0.029 (2)	−0.0030 (17)	0.0089 (18)	0.0047 (16)
C3	0.048 (3)	0.031 (2)	0.0154 (18)	−0.002 (2)	−0.0036 (17)	−0.0027 (16)
C4	0.060 (3)	0.049 (3)	0.022 (2)	0.003 (3)	−0.012 (2)	0.007 (2)
C5	0.030 (2)	0.0214 (19)	0.0207 (18)	0.0020 (16)	−0.0051 (15)	−0.0011 (15)
C6	0.0226 (18)	0.0202 (19)	0.0204 (17)	−0.0049 (15)	−0.0030 (14)	−0.0029 (14)
C7	0.030 (2)	0.029 (2)	0.0241 (19)	−0.0034 (17)	−0.0038 (16)	0.0019 (16)
C8	0.031 (2)	0.045 (3)	0.024 (2)	0.000 (2)	0.0029 (17)	−0.0030 (19)
C9	0.030 (2)	0.035 (2)	0.040 (2)	0.0004 (19)	0.0067 (18)	−0.012 (2)
C10	0.037 (2)	0.021 (2)	0.043 (2)	0.0019 (19)	0.0059 (19)	0.0042 (19)
C11	0.039 (2)	0.025 (2)	0.026 (2)	0.0008 (18)	0.0065 (17)	0.0040 (17)
C12	0.0228 (18)	0.0153 (17)	0.0181 (17)	0.0008 (14)	0.0013 (14)	−0.0014 (13)
C13	0.028 (2)	0.0181 (18)	0.0221 (18)	−0.0021 (15)	−0.0024 (15)	−0.0019 (15)
C14	0.039 (2)	0.027 (2)	0.031 (2)	−0.0071 (19)	−0.0092 (18)	−0.0058 (18)
C15	0.053 (3)	0.021 (2)	0.033 (2)	−0.007 (2)	−0.002 (2)	−0.0096 (18)
C16	0.041 (2)	0.0147 (18)	0.031 (2)	−0.0016 (17)	0.0077 (18)	−0.0027 (16)
C17	0.027 (2)	0.0147 (17)	0.0240 (18)	0.0003 (15)	0.0035 (15)	0.0006 (14)
C18	0.0238 (19)	0.0195 (17)	0.0158 (16)	0.0007 (15)	−0.0011 (14)	0.0022 (14)
C19	0.034 (2)	0.023 (2)	0.0209 (18)	−0.0007 (17)	−0.0041 (16)	0.0064 (15)
C20	0.039 (2)	0.030 (2)	0.024 (2)	0.0061 (19)	0.0007 (17)	0.0106 (17)
C21	0.040 (3)	0.041 (3)	0.0180 (19)	0.010 (2)	−0.0001 (17)	0.0045 (18)
C22	0.032 (2)	0.038 (2)	0.0235 (19)	0.0025 (19)	−0.0063 (16)	−0.0044 (18)
C23	0.026 (2)	0.028 (2)	0.0202 (18)	0.0003 (16)	−0.0022 (15)	−0.0013 (16)
C24	0.0180 (18)	0.0156 (17)	0.033 (2)	−0.0016 (14)	0.0089 (15)	−0.0006 (15)
C25	0.0171 (19)	0.025 (2)	0.052 (3)	−0.0001 (15)	0.0044 (18)	−0.0047 (19)
C26	0.022 (2)	0.032 (3)	0.090 (4)	−0.0070 (19)	0.017 (2)	−0.015 (3)
C27	0.039 (3)	0.029 (3)	0.094 (4)	−0.010 (2)	0.044 (3)	−0.012 (3)
C28	0.057 (3)	0.025 (2)	0.055 (3)	−0.007 (2)	0.036 (3)	−0.002 (2)
C29	0.036 (2)	0.021 (2)	0.029 (2)	−0.0002 (16)	0.0175 (17)	0.0001 (16)
C30	0.023 (3)	0.036 (3)	0.032 (3)	0.010 (2)	0.003 (2)	0.002 (3)
C30*	0.023 (3)	0.038 (3)	0.032 (3)	0.007 (2)	0.005 (2)	0.003 (3)
C31	0.0251 (18)	0.0232 (18)	0.0383 (19)	0.0050 (15)	0.0056 (15)	0.0064 (15)
C32	0.0306 (19)	0.0318 (19)	0.040 (2)	0.0095 (16)	0.0054 (16)	−0.0080 (17)
C33	0.031 (3)	0.031 (3)	0.034 (3)	0.000 (3)	0.015 (3)	−0.006 (2)
C33*	0.026 (3)	0.029 (3)	0.033 (3)	−0.001 (3)	0.014 (3)	−0.004 (2)
C34	0.0274 (19)	0.0266 (18)	0.0269 (18)	0.0019 (15)	0.0127 (15)	0.0060 (15)
C35	0.019 (2)	0.038 (3)	0.040 (2)	−0.0084 (17)	0.0117 (17)	−0.003 (2)
C36	0.025 (3)	0.045 (4)	0.043 (3)	−0.005 (3)	−0.001 (3)	−0.007 (3)
C36*	0.022 (3)	0.037 (4)	0.043 (3)	−0.007 (3)	−0.001 (3)	−0.008 (3)
C37	0.022 (3)	0.041 (3)	0.038 (3)	0.001 (2)	−0.005 (2)	−0.005 (3)
C37*	0.022 (3)	0.041 (3)	0.036 (3)	0.002 (2)	0.000 (2)	−0.004 (3)
Cl1	0.0749 (18)	0.090 (2)	0.110 (2)	0.0134 (14)	0.0146 (16)	−0.0585 (18)
Cl2	0.0568 (13)	0.0572 (16)	0.149 (3)	−0.0152 (12)	0.003 (2)	−0.022 (2)
C38	0.064 (2)	0.072 (2)	0.131 (3)	−0.002 (2)	0.014 (2)	−0.044 (2)
Cl1*	0.057 (2)	0.072 (3)	0.128 (4)	−0.015 (2)	0.010 (3)	−0.047 (3)
Cl2*	0.069 (2)	0.083 (3)	0.129 (3)	0.003 (2)	0.022 (2)	−0.050 (3)
C38*	0.063 (2)	0.071 (3)	0.132 (3)	−0.005 (2)	0.013 (3)	−0.043 (3)
Cl3	0.122 (7)	0.087 (5)	0.097 (5)	0.051 (5)	0.017 (4)	0.021 (4)
Cl4	0.112 (6)	0.086 (5)	0.094 (4)	0.035 (4)	0.019 (4)	−0.003 (3)
C39	0.116 (7)	0.085 (5)	0.092 (5)	0.045 (5)	0.018 (4)	0.006 (4)
F1	0.056 (3)	0.078 (3)	0.054 (3)	0.018 (3)	0.002 (2)	−0.020 (2)
F1*	0.085 (6)	0.058 (5)	0.050 (5)	−0.005 (5)	0.003 (5)	−0.016 (4)
F2	0.062 (3)	0.045 (2)	0.062 (3)	0.012 (2)	−0.012 (2)	−0.001 (2)
F2*	0.076 (5)	0.062 (4)	0.071 (4)	0.008 (4)	0.019 (4)	0.016 (4)
F3	0.125 (4)	0.061 (3)	0.026 (2)	−0.015 (3)	0.020 (3)	−0.001 (2)
F3*	0.081 (6)	0.076 (5)	0.041 (4)	0.019 (5)	0.013 (5)	−0.001 (4)
F4	0.068 (4)	0.053 (3)	0.055 (3)	0.000 (2)	0.016 (3)	0.008 (2)
F4*	0.099 (7)	0.048 (5)	0.048 (5)	−0.017 (5)	0.039 (6)	0.002 (4)
B1	0.067 (3)	0.049 (3)	0.038 (2)	0.012 (3)	0.016 (2)	0.005 (2)

### (4-Benzyl-1-ethyl-1,2,4-triazol-5-ylidene)[(1,2,5,6-η)-cycloocta-1,5-diene](tricyclohexylphosphane)iridium(I) tetrafluoridoborate dicholoromethane sesquisolvate (6) Geometric parameters (Å, º)

**Table:**

Ir1—P1	2.3707 (9)	C24—C29	1.529 (6)
Ir1—C30	2.218 (14)	C25—H25A	0.9900
Ir1—C30*	2.147 (15)	C25—H25B	0.9900
Ir1—C31	2.207 (4)	C25—C26	1.527 (6)
Ir1—C34	2.181 (4)	C26—H26A	0.9900
Ir1—C35	2.196 (4)	C26—H26B	0.9900
P1—C12	1.849 (4)	C26—C27	1.513 (9)
P1—C18	1.862 (4)	C27—H27A	0.9900
P1—C24	1.864 (4)	C27—H27B	0.9900
N1—N2	1.379 (5)	C27—C28	1.512 (8)
N1—C1	1.352 (5)	C28—H28A	0.9900
N1—C3	1.461 (5)	C28—H28B	0.9900
N2—C2	1.296 (6)	C28—C29	1.525 (6)
N3—C1	1.369 (5)	C29—H29A	0.9900
N3—C2	1.364 (5)	C29—H29B	0.9900
N3—C5	1.466 (5)	C30—H30	1.0000
Ir1—C1	2.034 (4)	C30—C31	1.420 (8)
C2—H2	0.9500	C30—C37	1.536 (8)
C3—H3A	0.9900	C30*—H30*	1.0000
C3—H3B	0.9900	C30*—C31	1.356 (8)
C3—C4	1.520 (6)	C30*—C37*	1.515 (8)
C4—H4A	0.9800	C31—H31A	1.0000
C4—H4B	0.9800	C31—H31	1.0000
C4—H4C	0.9800	C31—C32	1.498 (6)
C5—H5A	0.9900	C32—H32A	0.9900
C5—H5B	0.9900	C32—H32B	0.9900
C5—C6	1.506 (5)	C32—H32C	0.9900
C6—C7	1.389 (5)	C32—H32D	0.9900
C6—C11	1.391 (6)	C32—C33	1.498 (8)
C7—H7	0.9500	C32—C33*	1.545 (8)
C7—C8	1.390 (6)	C33—H33A	0.9900
C8—H8	0.9500	C33—H33B	0.9900
C8—C9	1.373 (7)	C33—C34	1.530 (7)
C9—H9	0.9500	C33*—H33C	0.9900
C9—C10	1.386 (6)	C33*—H33D	0.9900
C10—H10	0.9500	C33*—C34	1.524 (7)
C10—C11	1.380 (6)	C34—H34	1.0000
C11—H11	0.9500	C34—H34A	1.0000
C12—H12	1.0000	C34—C35	1.366 (5)
C12—C13	1.527 (5)	C35—H35A	1.0000
C12—C17	1.541 (5)	C35—H35	1.0000
C13—H13A	0.9900	C35—C36	1.497 (8)
C13—H13B	0.9900	C35—C36*	1.572 (7)
C13—C14	1.534 (5)	C36—H36A	0.9900
C14—H14A	0.9900	C36—H36B	0.9900
C14—H14B	0.9900	C36—C37	1.517 (9)
C14—C15	1.523 (6)	C36*—H36C	0.9900
C15—H15A	0.9900	C36*—H36D	0.9900
C15—H15B	0.9900	C36*—C37*	1.551 (8)
C15—C16	1.523 (6)	C37—H37A	0.9900
C16—H16A	0.9900	C37—H37B	0.9900
C16—H16B	0.9900	C37*—H37C	0.9900
C16—C17	1.526 (5)	C37*—H37D	0.9900
C17—H17A	0.9900	Cl1—C38	1.749 (17)
C17—H17B	0.9900	Cl2—C38	1.731 (15)
C18—H18	1.0000	C38—H38A	0.9900
C18—C19	1.538 (5)	C38—H38B	0.9900
C18—C23	1.537 (5)	Cl1*—C38*	1.86 (4)
C19—H19A	0.9900	Cl2*—C38*	1.74 (4)
C19—H19B	0.9900	C38*—H38C	0.9900
C19—C20	1.531 (5)	C38*—H38D	0.9900
C20—H20A	0.9900	Cl3—C39	1.7772
C20—H20B	0.9900	Cl4—C39	1.7657
C20—C21	1.521 (6)	C39—H39A	0.9900
C21—H21A	0.9900	C39—H39B	0.9900
C21—H21B	0.9900	F1—B1	1.360 (6)
C21—C22	1.523 (6)	F1*—B1	1.300 (6)
C22—H22A	0.9900	F2—B1	1.456 (6)
C22—H22B	0.9900	F2*—B1	1.325 (6)
C22—C23	1.536 (5)	F3—B1	1.314 (6)
C23—H23A	0.9900	F3*—B1	1.401 (6)
C23—H23B	0.9900	F4—B1	1.350 (6)
C24—H24	1.0000	F4*—B1	1.378 (7)
C24—C25	1.545 (6)		
			
N1—C1—N3	102.2 (3)	C24—C25—H25A	109.7
C1—Ir1—P1	93.42 (10)	C24—C25—H25B	109.7
C1—Ir1—C30	94.8 (3)	H25A—C25—H25B	108.2
C1—Ir1—C30*	85.9 (3)	C26—C25—C24	109.9 (4)
C1—Ir1—C31	87.44 (15)	C26—C25—H25A	109.7
C1—Ir1—C34	154.59 (15)	C26—C25—H25B	109.7
C1—Ir1—C35	166.87 (15)	C25—C26—H26A	109.1
C30—Ir1—P1	152.8 (2)	C25—C26—H26B	109.1
C30*—Ir1—P1	154.5 (2)	H26A—C26—H26B	107.8
C30*—Ir1—C31	36.2 (2)	C27—C26—C25	112.5 (4)
C30*—Ir1—C34	95.9 (3)	C27—C26—H26A	109.1
C30*—Ir1—C35	84.7 (3)	C27—C26—H26B	109.1
C31—Ir1—P1	169.21 (11)	C26—C27—H27A	109.3
C31—Ir1—C30	37.4 (2)	C26—C27—H27B	109.3
C34—Ir1—P1	95.47 (11)	H27A—C27—H27B	107.9
C34—Ir1—C30	88.0 (3)	C28—C27—C26	111.8 (4)
C34—Ir1—C31	79.54 (15)	C28—C27—H27A	109.3
C34—Ir1—C35	36.35 (14)	C28—C27—H27B	109.3
C35—Ir1—P1	91.09 (12)	C27—C28—H28A	109.4
C35—Ir1—C30	75.9 (3)	C27—C28—H28B	109.4
C35—Ir1—C31	90.43 (16)	C27—C28—C29	111.2 (4)
C12—P1—Ir1	110.05 (12)	H28A—C28—H28B	108.0
C12—P1—C18	101.93 (17)	C29—C28—H28A	109.4
C12—P1—C24	109.16 (17)	C29—C28—H28B	109.4
C18—P1—Ir1	119.37 (13)	C24—C29—H29A	109.4
C18—P1—C24	102.49 (18)	C24—C29—H29B	109.4
C24—P1—Ir1	112.92 (13)	C28—C29—C24	111.1 (4)
N2—N1—C3	117.4 (3)	C28—C29—H29A	109.4
C1—N1—N2	113.8 (3)	C28—C29—H29B	109.4
C1—N1—C3	128.7 (3)	H29A—C29—H29B	108.0
C2—N2—N1	103.5 (3)	Ir1—C30—H30	113.4
C1—N3—C5	125.5 (3)	C31—C30—Ir1	70.9 (5)
C2—N3—C1	109.1 (3)	C31—C30—H30	113.4
C2—N3—C5	125.4 (3)	C31—C30—C37	125.0 (8)
N1—C1—Ir1	131.5 (3)	C37—C30—Ir1	113.4 (8)
N3—C1—Ir1	126.2 (3)	C37—C30—H30	113.4
N2—C2—N3	111.4 (4)	Ir1—C30*—H30*	114.6
N2—C2—H2	124.3	C31—C30*—Ir1	74.3 (6)
N3—C2—H2	124.3	C31—C30*—H30*	114.6
N1—C3—H3A	109.4	C31—C30*—C37*	122.3 (8)
N1—C3—H3B	109.4	C37*—C30*—Ir1	109.4 (8)
N1—C3—C4	111.2 (4)	C37*—C30*—H30*	114.6
H3A—C3—H3B	108.0	Ir1—C31—H31A	111.5
C4—C3—H3A	109.4	Ir1—C31—H31	115.7
C4—C3—H3B	109.4	C30—C31—Ir1	71.7 (6)
C3—C4—H4A	109.5	C30—C31—H31	115.7
C3—C4—H4B	109.5	C30—C31—C32	117.8 (5)
C3—C4—H4C	109.5	C30*—C31—Ir1	69.5 (7)
H4A—C4—H4B	109.5	C30*—C31—H31A	111.5
H4A—C4—H4C	109.5	C30*—C31—C32	131.8 (6)
H4B—C4—H4C	109.5	C32—C31—Ir1	112.8 (3)
N3—C5—H5A	108.7	C32—C31—H31A	111.5
N3—C5—H5B	108.7	C32—C31—H31	115.7
N3—C5—C6	114.2 (3)	C31—C32—H32A	108.1
H5A—C5—H5B	107.6	C31—C32—H32B	108.1
C6—C5—H5A	108.7	C31—C32—H32C	108.5
C6—C5—H5B	108.7	C31—C32—H32D	108.5
C7—C6—C5	119.0 (4)	C31—C32—C33*	115.1 (4)
C7—C6—C11	118.5 (4)	H32A—C32—H32B	107.3
C11—C6—C5	122.4 (3)	H32C—C32—H32D	107.5
C6—C7—H7	119.6	C33—C32—C31	116.6 (4)
C6—C7—C8	120.8 (4)	C33—C32—H32A	108.1
C8—C7—H7	119.6	C33—C32—H32B	108.1
C7—C8—H8	120.1	C33*—C32—H32C	108.5
C9—C8—C7	119.9 (4)	C33*—C32—H32D	108.5
C9—C8—H8	120.1	C32—C33—H33A	108.8
C8—C9—H9	120.0	C32—C33—H33B	108.8
C8—C9—C10	120.0 (4)	C32—C33—C34	114.0 (6)
C10—C9—H9	120.0	H33A—C33—H33B	107.7
C9—C10—H10	119.9	C34—C33—H33A	108.8
C11—C10—C9	120.1 (4)	C34—C33—H33B	108.8
C11—C10—H10	119.9	C32—C33*—H33C	109.3
C6—C11—H11	119.7	C32—C33*—H33D	109.3
C10—C11—C6	120.7 (4)	H33C—C33*—H33D	108.0
C10—C11—H11	119.7	C34—C33*—C32	111.6 (6)
P1—C12—H12	104.4	C34—C33*—H33C	109.3
C13—C12—P1	115.2 (3)	C34—C33*—H33D	109.3
C13—C12—H12	104.4	Ir1—C34—H34	115.5
C13—C12—C17	111.1 (3)	Ir1—C34—H34A	110.5
C17—C12—P1	115.8 (3)	C33—C34—Ir1	114.7 (4)
C17—C12—H12	104.4	C33—C34—H34	115.5
C12—C13—H13A	109.7	C33*—C34—Ir1	111.0 (4)
C12—C13—H13B	109.7	C33*—C34—H34A	110.5
C12—C13—C14	109.8 (3)	C35—C34—Ir1	72.4 (2)
H13A—C13—H13B	108.2	C35—C34—C33	116.2 (5)
C14—C13—H13A	109.7	C35—C34—C33*	134.0 (5)
C14—C13—H13B	109.7	C35—C34—H34	115.5
C13—C14—H14A	109.3	C35—C34—H34A	110.5
C13—C14—H14B	109.3	Ir1—C35—H35A	118.2
H14A—C14—H14B	108.0	Ir1—C35—H35	108.4
C15—C14—C13	111.4 (4)	C34—C35—Ir1	71.2 (2)
C15—C14—H14A	109.3	C34—C35—H35A	118.2
C15—C14—H14B	109.3	C34—C35—H35	108.4
C14—C15—H15A	109.3	C34—C35—C36	136.6 (6)
C14—C15—H15B	109.3	C34—C35—C36*	114.6 (5)
H15A—C15—H15B	108.0	C36—C35—Ir1	116.9 (5)
C16—C15—C14	111.5 (3)	C36—C35—H35	108.4
C16—C15—H15A	109.3	C36*—C35—Ir1	107.2 (4)
C16—C15—H15B	109.3	C36*—C35—H35A	118.2
C15—C16—H16A	109.4	C35—C36—H36A	110.3
C15—C16—H16B	109.4	C35—C36—H36B	110.3
C15—C16—C17	111.3 (3)	C35—C36—C37	107.0 (7)
H16A—C16—H16B	108.0	H36A—C36—H36B	108.6
C17—C16—H16A	109.4	C37—C36—H36A	110.3
C17—C16—H16B	109.4	C37—C36—H36B	110.3
C12—C17—H17A	109.7	C35—C36*—H36C	108.3
C12—C17—H17B	109.7	C35—C36*—H36D	108.3
C16—C17—C12	109.7 (3)	H36C—C36*—H36D	107.4
C16—C17—H17A	109.7	C37*—C36*—C35	115.7 (7)
C16—C17—H17B	109.7	C37*—C36*—H36C	108.3
H17A—C17—H17B	108.2	C37*—C36*—H36D	108.3
P1—C18—H18	105.5	C30—C37—H37A	108.2
C19—C18—P1	116.4 (3)	C30—C37—H37B	108.2
C19—C18—H18	105.5	C36—C37—C30	116.4 (9)
C23—C18—P1	111.8 (3)	C36—C37—H37A	108.2
C23—C18—H18	105.5	C36—C37—H37B	108.2
C23—C18—C19	111.0 (3)	H37A—C37—H37B	107.3
C18—C19—H19A	109.5	C30*—C37*—C36*	112.2 (8)
C18—C19—H19B	109.5	C30*—C37*—H37C	109.2
H19A—C19—H19B	108.1	C30*—C37*—H37D	109.2
C20—C19—C18	110.7 (3)	C36*—C37*—H37C	109.2
C20—C19—H19A	109.5	C36*—C37*—H37D	109.2
C20—C19—H19B	109.5	H37C—C37*—H37D	107.9
C19—C20—H20A	109.4	Cl1—C38—H38A	108.8
C19—C20—H20B	109.4	Cl1—C38—H38B	108.8
H20A—C20—H20B	108.0	Cl2—C38—Cl1	113.8 (9)
C21—C20—C19	111.4 (4)	Cl2—C38—H38A	108.8
C21—C20—H20A	109.4	Cl2—C38—H38B	108.8
C21—C20—H20B	109.4	H38A—C38—H38B	107.7
C20—C21—H21A	109.4	Cl1*—C38*—H38C	109.5
C20—C21—H21B	109.4	Cl1*—C38*—H38D	109.5
C20—C21—C22	111.2 (3)	Cl2*—C38*—Cl1*	111 (2)
H21A—C21—H21B	108.0	Cl2*—C38*—H38C	109.5
C22—C21—H21A	109.4	Cl2*—C38*—H38D	109.5
C22—C21—H21B	109.4	H38C—C38*—H38D	108.0
C21—C22—H22A	109.4	Cl3—C39—H39A	109.5
C21—C22—H22B	109.4	Cl3—C39—H39B	109.5
C21—C22—C23	111.4 (3)	Cl4—C39—Cl3	110.8
H22A—C22—H22B	108.0	Cl4—C39—H39A	109.5
C23—C22—H22A	109.4	Cl4—C39—H39B	109.5
C23—C22—H22B	109.4	H39A—C39—H39B	108.1
C18—C23—H23A	109.4	F1—B1—F2	100.1 (4)
C18—C23—H23B	109.4	F1*—B1—F2*	118.3 (7)
C22—C23—C18	111.1 (3)	F1*—B1—F3*	108.6 (6)
C22—C23—H23A	109.4	F1*—B1—F4*	111.4 (7)
C22—C23—H23B	109.4	F2*—B1—F3*	103.9 (6)
H23A—C23—H23B	108.0	F2*—B1—F4*	110.3 (7)
P1—C24—H24	105.3	F3—B1—F1	115.3 (6)
C25—C24—P1	118.8 (3)	F3—B1—F2	107.3 (5)
C25—C24—H24	105.3	F3—B1—F4	114.1 (5)
C29—C24—P1	112.2 (3)	F4—B1—F1	113.6 (5)
C29—C24—H24	105.3	F4—B1—F2	104.6 (5)
C29—C24—C25	108.9 (3)	F4*—B1—F3*	102.8 (7)
			
Ir1—P1—C12—C13	42.0 (3)	C12—P1—C24—C25	−82.2 (3)
Ir1—P1—C12—C17	174.0 (2)	C12—P1—C24—C29	46.4 (3)
Ir1—P1—C18—C19	76.6 (3)	C12—C13—C14—C15	−56.1 (5)
Ir1—P1—C18—C23	−52.4 (3)	C13—C12—C17—C16	−58.2 (4)
Ir1—P1—C24—C25	155.1 (3)	C13—C14—C15—C16	55.2 (5)
Ir1—P1—C24—C29	−76.4 (3)	C14—C15—C16—C17	−55.6 (5)
Ir1—C30—C31—C32	−106.7 (5)	C15—C16—C17—C12	56.4 (4)
Ir1—C30—C37—C36	−5.5 (11)	C17—C12—C13—C14	57.9 (4)
Ir1—C30*—C31—C32	−102.8 (7)	C18—P1—C12—C13	169.6 (3)
Ir1—C30*—C37*—C36*	35.2 (10)	C18—P1—C12—C17	−58.4 (3)
Ir1—C31—C32—C33	−17.7 (7)	C18—P1—C24—C25	25.4 (3)
Ir1—C31—C32—C33*	3.0 (6)	C18—P1—C24—C29	153.9 (3)
Ir1—C34—C35—C36	−109.7 (8)	C18—C19—C20—C21	56.4 (5)
Ir1—C34—C35—C36*	−100.8 (5)	C19—C18—C23—C22	55.0 (4)
Ir1—C35—C36—C37	−36.4 (10)	C19—C20—C21—C22	−56.5 (5)
Ir1—C35—C36*—C37*	15.3 (9)	C20—C21—C22—C23	55.7 (5)
P1—C12—C13—C14	−168.0 (3)	C21—C22—C23—C18	−55.0 (5)
P1—C12—C17—C16	167.9 (3)	C23—C18—C19—C20	−55.5 (4)
P1—C18—C19—C20	175.0 (3)	C24—P1—C12—C13	−82.5 (3)
P1—C18—C23—C22	−173.2 (3)	C24—P1—C12—C17	49.5 (3)
P1—C24—C25—C26	−171.9 (3)	C24—P1—C18—C19	−157.8 (3)
P1—C24—C29—C28	167.2 (3)	C24—P1—C18—C23	73.2 (3)
N1—N2—C2—N3	0.3 (5)	C24—C25—C26—C27	−56.0 (5)
N2—N1—C1—Ir1	177.5 (3)	C25—C24—C29—C28	−59.2 (4)
N2—N1—C1—N3	1.3 (4)	C25—C26—C27—C28	53.7 (5)
N2—N1—C3—C4	−61.7 (5)	C26—C27—C28—C29	−53.4 (5)
N3—C5—C6—C7	142.8 (4)	C27—C28—C29—C24	57.1 (5)
N3—C5—C6—C11	−39.6 (5)	C29—C24—C25—C26	58.0 (5)
C1—N1—N2—C2	−1.1 (5)	C30—C31—C32—C33	62.9 (10)
C1—N1—C3—C4	121.6 (5)	C30*—C31—C32—C33*	85.2 (11)
C1—N3—C2—N2	0.5 (5)	C31—C30—C37—C36	−87.9 (14)
C1—N3—C5—C6	−85.3 (5)	C31—C30*—C37*—C36*	−48.1 (15)
C2—N3—C1—Ir1	−177.5 (3)	C31—C32—C33—C34	12.7 (10)
C2—N3—C1—N1	−1.1 (4)	C31—C32—C33*—C34	−24.8 (8)
C2—N3—C5—C6	94.2 (5)	C32—C33—C34—Ir1	−1.4 (9)
C3—N1—N2—C2	−178.2 (4)	C32—C33—C34—C35	−83.2 (8)
C3—N1—C1—Ir1	−5.8 (6)	C32—C33*—C34—Ir1	34.8 (7)
C3—N1—C1—N3	178.1 (4)	C32—C33*—C34—C35	−50.7 (9)
C5—N3—C1—Ir1	2.1 (5)	C33—C34—C35—Ir1	109.4 (4)
C5—N3—C1—N1	178.5 (3)	C33—C34—C35—C36	−0.3 (10)
C5—N3—C2—N2	−179.1 (4)	C33*—C34—C35—Ir1	102.5 (6)
C5—C6—C7—C8	176.8 (4)	C33*—C34—C35—C36*	1.7 (9)
C5—C6—C11—C10	−177.4 (4)	C34—C35—C36—C37	54.3 (12)
C6—C7—C8—C9	1.0 (7)	C34—C35—C36*—C37*	92.0 (9)
C7—C6—C11—C10	0.2 (6)	C35—C36—C37—C30	25.9 (12)
C7—C8—C9—C10	−0.4 (7)	C35—C36*—C37*—C30*	−34.3 (12)
C8—C9—C10—C11	−0.3 (7)	C37—C30—C31—Ir1	105.7 (12)
C9—C10—C11—C6	0.4 (7)	C37—C30—C31—C32	−1.0 (15)
C11—C6—C7—C8	−0.9 (6)	C37*—C30*—C31—Ir1	103.3 (12)
C12—P1—C18—C19	−44.8 (3)	C37*—C30*—C31—C32	0.6 (18)
C12—P1—C18—C23	−173.8 (3)		

### (4-Benzyl-1-ethyl-1,2,4-triazol-5-ylidene)[(1,2,5,6-η)-cycloocta-1,5-diene](tricyclohexylphosphane)iridium(I) tetrafluoridoborate dicholoromethane sesquisolvate (6) Hydrogen-bond geometry (Å, º)

**Table:**

*D*—H···*A*	*D*—H	H···*A*	*D*···*A*	*D*—H···*A*
C2—H2···F2^i^	0.95	2.25	3.140 (6)	155
C7—H7···F3^ii^	0.95	2.39	3.182 (7)	141
C8—H8···F4^ii^	0.95	2.61	3.424 (9)	144
C13—H13*A*···F1*	0.99	2.47	3.263 (13)	137
C22—H22*B*···F2*^ii^	0.99	2.55	3.340 (11)	137
C23—H23*B*···F3^ii^	0.99	2.44	3.244 (6)	139
C24—H24···N3	1.00	2.67	3.497 (5)	140
C29—H29*B*···N1	0.99	2.55	3.383 (5)	142
C33*—H33*C*···F3*^ii^	0.99	2.06	2.907 (11)	143
C38—H38*A*···F1	0.99	1.98	2.93 (2)	158
C38*—H38*D*···F3*	0.99	2.44	3.37 (4)	155

Symmetry codes: (i) −*x*+1, *y*+1/2, −*z*+1/2; (ii) *x*, −*y*+1/2, *z*+1/2.

### Selected Geometric Parameters (Å, °) for 2

**Table:**

N1—C1	1.315 (3)
N3—C1	1.339 (3)
	
N1—C1—N3	107.1 (2)

### Selected Geometric Parameters (Å, °) for 3

**Table:**

N1—C1	1.343 (3)
N3—C1	1.367 (3)
Rh1—C1	2.3960 (6)
	
N1—C1—N3	102.7 (2)
C1—Rh1—Cl1	89.14 (7)

### Selected Geometric Parameters (Å, °) for 5

**Table:**

N1—C1	1.336 (8)
N1`—C1`	1.340 (8)
N3—C1	1.354 (8)
N3`—C1`	1.380 (8)
Ir1—C1	2.039 (6)
Ir1`—C1`	2.029 (6)
	
N1—C1—N3	103.8 (5)
N1'—C1'—N3'	102.7 (5)
C1—Ir1—P1	93.14 (17)
C1`—Ir1`—P1`	94.64 (18)

## References

[bb1] Albert, D. R., Gau, M. & Rajaseelan, E. (2025). *IUCrData*, **10**, x250092. 10.1107/S2414314625000926 PMC11904631 40092360

[bb2] Albrecht, M., Miecznikowski, J. R., Samuel, A., Faller, J. W. & Crabtree, R. H. (2002). *Organometallics*, **21**, 3596–3604.

[bb3] Bortenschlager, M., Schütz, J., von Preysing, D., Nuyken, O., Herrmann, W. A. & Weberskirch, R. (2005). *J. Organomet. Chem.* **690**, 6233–6237.

[bb5] Bourissou, D., Guerret, O., Gabbaï, F. P. & Bertrand, G. (2000). *Chem. Rev.* **100**, 39–92. 10.1021/cr940472u 11749234

[bb6] Castaldi, K. T., Astashkin, A. V., Albert, D. R. & Rajaseelan, E. (2021). *IUCrData*, **6**, x211142. 10.1107/S2414314621011421 PMC9462289 36337466

[bb7] Cazin, C. S. J. (2013). *Dalton Trans.* **42**, 7254. 10.1039/c2dt32277c 23235534

[bb8] Chianese, A. R., Kovacevic, A., Zeglis, B. M., Faller, J. W. & Crabtree, R. H. (2004). *Organometallics*, **23**, 2461–2468.

[bb9] Díez-González, S., Marion, N. & Nolan, S. P. (2009). *Chem. Rev.* **109**, 3612–3676. 10.1021/cr900074m 19588961

[bb10] Díez-González, S. & Nolan, S. P. (2007). *Coord. Chem. Rev.* **251**, 874–883.

[bb11] Dolomanov, O. V., Bourhis, L. J., Gildea, R. J., Howard, J. A. K. & Puschmann, H. (2009). *J. Appl. Cryst.* **42**, 339–341.

[bb12] Dwivedi, S., Gupta, S. & Das, S. (2014). *Curr. Organocatalysis*, **1**, 13–39.

[bb13] El Bakri, Y., Harmaoui, A., Sebhaoui, J., Ramli, Y., Essassi, E. M. & Mague, J. T. (2016). *IUCrData*, **1**, x161819.

[bb14] Gnanamgari, D., Moores, A., Rajaseelan, E. & Crabtree, R. H. (2007). *Organometallics*, **26**, 1226–1230.

[bb15] Gražulis, S., Chateigner, D., Downs, R. T., Yokochi, A. F. T., Quirós, M., Lutterotti, L., Manakova, E., Butkus, J., Moeck, P. & Le Bail, A. (2009). *J. Appl. Cryst.* **42**, 726–729. 10.1107/S0021889809016690 PMC3253730 22477773

[bb16] Guino-o, M. A., Talbot, M. O., Slitts, M. M., Pham, T. N., Audi, M. C. & Janzen, D. E. (2015). *Acta Cryst.* E**71**, 628–635. 10.1107/S2056989015009019 PMC4459379 26090137

[bb17] Gusev, D. G. (2009). *Organometallics*, **28**, 6458–6461.

[bb18] Herrmann, W. A. & Köcher, C. (1997). *Angew. Chem. Int. Ed. Engl.* **36**, 2162–2187.

[bb19] Herrmann, W. A., Schütz, J., Frey, G. D. & Herdtweck, E. (2006). *Organometallics*, **25**, 2437–2448.

[bb20] Hillier, A. C., Lee, H. M., Stevens, E. D. & Nolan, S. P. (2001). *Organometallics*, **20**, 4246–4252.

[bb21] Idrees, K. B., Astashkin, A. V. & Rajaseelan, E. (2017*b*). *IUCrData*, **2**, x171081.

[bb22] Idrees, K. B., Rutledge, W. J., Roberts, S. A. & Rajaseelan, E. (2017*a*). *IUCrData*, **2**, x171411.

[bb23] Kumasaki, M., Gontani, S., Mori, K., Matsumoto, S. & Inoue, K. (2021). *Acta Cryst.* C**77**, 197–201. 10.1107/S2053229621003260 33949334

[bb24] Lerch, G. L., Gau, M., Albert, D. R. & Rajaseelan, E. (2024). *IUCrData*, **9**, x240941. 10.1107/S2414314624009416 PMC11451034 39371671

[bb25] Mata, J. A., Chianese, A. R., Miecznikowski, J. R., Poyatos, M., Peris, E., Faller, J. W. & Crabtree, R. H. (2004). *Organometallics*, **23**, 1253–1263.

[bb26] Maynard, A., Keller, T. M., Gau, M., Albert, D. R. & Rajaseelan, E. (2023). *IUCrData*, **8**, x230784. 10.1107/S2414314623007848 PMC10561235 37818465

[bb27] Newman, E. B., Astashkin, A. V., Albert, D. R. & Rajaseelan, E. (2021). *IUCrData*, **6**, x210836. 10.1107/S2414314621008361 PMC9462361 36339453

[bb28] Nichol, G. S., Rajaseelan, J., Anna, L. J. & Rajaseelan, E. (2009). *Eur. J. Inorg. Chem.* pp. 4320–4328.

[bb29] Nichol, G. S., Rajaseelan, J., Walton, D. P. & Rajaseelan, E. (2011). *Acta Cryst.* E**67**, m1860–m1861. 10.1107/S1600536811049890 PMC3238752 22199629

[bb30] Nichol, G. S., Stasiw, D., Anna, L. J. & Rajaseelan, E. (2010). *Acta Cryst.* E**66**, m1114. 10.1107/S1600536810031727 PMC3008037 21588520

[bb31] Nichol, G. S., Walton, D. P., Anna, L. J. & Rajaseelan, E. (2012). *Acta Cryst.* E**68**, m158–m159. 10.1107/S1600536812000992 PMC3274890 22346837

[bb32] Parsons, S., Flack, H. D. & Wagner, T. (2013). *Acta Cryst.* B**69**, 249–259. 10.1107/S2052519213010014 PMC3661305 23719469

[bb43] Rigaku OD (2024). *CrysAlis PRO*. Rigaku Oxford Diffraction, Yarnton, England.

[bb33] Rood, J., Subedi, C. B., Risell, J. P., Astashkin, A. V. & Rajaseelan, E. (2021). *IUCrData*, **6**, x210597. 10.1107/S2414314621005976 PMC9462349 36337330

[bb34] Rovis, T. & Nolan, S. (2013). *Synlett*, **24**, 1188–1189.

[bb35] Ruff, A., Kirby, C., Chan, B. C. & O’Connor, A. R. (2016). *Organometallics*, **35**, 327–335.

[bb36] Rushlow, J., Astashkin, A. V., Albert, D. R. & Rajaseelan, E. (2021). *IUCrData*, **6**, x210811. 10.1107/S2414314621008117 PMC9462365 36339455

[bb37] Sheldrick, G. M. (2015*a*). *Acta Cryst.* A**71**, 3–8.

[bb38] Sheldrick, G. M. (2015*b*). *Acta Cryst.* C**71**, 3–8.

[bb39] Wang, H. M. J. & Lin, I. J. B. (1998). *Organometallics*, **17**, 972–975.

[bb40] Weskamp, T., Böhm, V. P. W. & Herrmann, W. A. (2000). *J. Organo­met. Chem.* **600**, 12–22.

[bb41] Westrip, S. P. (2010). *J. Appl. Cryst.* **43**, 920–925.

[bb42] Zuo, W., Tauer, S., Prokopchuk, D. E. & Morris, R. H. (2014). *Organometallics*, **33**, 5791–5801.

